# Effect of Short-term Integrated Palliative Care on Patient-Reported Outcomes Among Patients Severely Affected With Long-term Neurological Conditions: A Randomized Clinical Trial

**DOI:** 10.1001/jamanetworkopen.2020.15061

**Published:** 2020-08-28

**Authors:** Wei Gao, Rebecca Wilson, Nilay Hepgul, Deokhee Yi, Catherine Evans, Sabrina Bajwah, Vincent Crosby, Andrew Wilcock, Fiona Lindsay, Anthony Byrne, Carolyn Young, Karen Groves, Clare Smith, Rachel Burman, K. Ray Chaudhuri, Eli Silber, Irene J. Higginson

**Affiliations:** 1 Cicely Saunders Institute of Palliative Care, Policy and Rehabilitation, King's College London, London, United Kingdom; 2 Sussex Community NHS Foundation Trust, United Kingdom; 3 Department of Palliative Medicine, Nottingham University Hospitals NHS Trust, Nottingham, United Kingdom; 4 Faculty of Medicine & Health Sciences, University of Nottingham, Nottingham, United Kingdom; 5 Marlets Hospice, Hove, United Kingdom; 6 Marie Curie Palliative Care Research Centre, Cardiff University, Cardiff, United Kingdom; 7 The Walton Centre NHS Foundation Trust and University of Liverpool, Liverpool, United Kingdom; 8 Queenscourt Hospice, Southport, United Kingdom; 9 Department of Palliative Care, Ashford and St Peter’s Hospitals NHS Foundation Trust, Surrey, United Kingdom; 10 Department of Palliative Care, King’s College Hospital, London, United Kingdom; 11 Parkinson Foundation International Centre of Excellence, Kings College Hospital and Kings College London, London, United Kingdom; 12 Department of Neurology, King’s College Hospital NHS Trust, London, United Kingdom

## Abstract

**Importance:**

Palliative care has shown benefits in reducing symptom intensity and quality of life in patients with advanced cancer. However, high-quality evidence to support palliative care policy and service developments for patients with long-term neurological conditions (LTNCs) is lacking.

**Objective:**

To determine the effectiveness of a short-term integrated palliative care (SIPC) intervention for people with LTNCs.

**Design, Setting, and Participants:**

Multicenter, phase 3, randomized clinical trial conducted from April 1, 2015, to November 30, 2017, with a last follow-up date of May 31, 2018, in 7 UK hospitals with both neurology and palliative care services. A total of 535 patients with LTNC were assessed for eligibility and 350 were randomized. Inclusion criteria were patients 18 years or older with any advanced stage of multiple sclerosis, motor neuron disease, idiopathic Parkinson disease multiple system atrophy, or progressive supranuclear palsy. Data were analyzed from November 2018 to March 2019.

**Interventions:**

Patients were randomized 1:1 using minimization method to receive SIPC (intervention, n = 176) or standard care (control, n = 174).

**Main Outcomes and Measures:**

Primary outcome was change in 8 key palliative care symptoms from baseline to 12-weeks, measured by the Integrated Palliative care Outcome Scale for neurological conditions. Secondary outcomes included change in the burden of other symptoms, health-related quality of life, caregiver burden, and costs. Data were collected and analyzed blindly by intention to treat.

**Results:**

A total of 350 patients (mean [SD] age 67 [12] years; years since diagnosis, 12 [range, 0-56]; 51% men; 49% requiring considerable assistance) with an advanced stage of LTNC were recruited, along with informal caregivers (n = 229). There were no between-group differences in primary outcome (effect size, −0.16; 95% CI, −0.37 to 0.05), any other patient-reported outcomes, adverse events, or survival. Although there was more symptom reduction in the SIPC group in relation to mean change in primary outcome, the difference between the groups was not statistically significant (−0.78; 95% CI, −1.29 to −0.26 vs −0.28; 95% CI, −0.82 to 0.26; *P* = .14). There was a decrease in mean health and social care costs from baseline to 12 weeks −$1367 (95% CI, −$2450 to −$282) in the SIPC group and −653 (95% CI, −$1839 to −$532) in the control group, but this difference was not statistically significant (*P* = .12). SIPC was perceived by patients and caregivers as building resilience, attending to function and deficits, and enabling caregivers.

**Conclusions and Relevance:**

In this study, SIPC was not statistically significantly different from standard care for the patient-reported outcomes. However, SIPC was associated with lower cost, and in qualitative analysis was well-received by patients and caregivers, and there were no safety concerns. Further research is warranted.

**Trial Registration:**

isrctn.org Identifier: ISRCTN18337380

## Key Points

**Question:**

Can short-term integrated palliative care (SIPC) improve patient-reported outcomes among patients with long-term neurological conditions?

**Findings:**

In this randomized clinical trial of 350 patients and 229 informal caregivers, no group difference was found in any of the evaluated outcomes, adverse events, or survival.

**Meaning:**

No differences were found between SIPC and standard care for the patient-reported outcomes.

## Teaser

This randomized clinical trial evaluates the effect of short-term integrated palliative care vs standard care among patients severely affected by long-term neurological conditions.

## Introduction

Palliative care, which focuses on improving quality of life (QoL) through a multidisciplinary and holistic care approach, may offer an additional layer of support for those affected by chronic life-limiting illnesses. Palliative care has shown benefits in symptom intensity and QoL in patients with advanced cancer, and possible survival improvement.^1,2,3,4^ Despite experiencing problems and care challenges similar to patients with advanced cancer, patients with chronic non-cancer conditions including long-term neurological conditions (LTNCs) are less likely to receive palliative care.^5,6^

Neurological disorders are a major health burden, accounting globally for 10% of disability-adjusted life-years and 17% of deaths.^7^ The LTNCs are a range of progressive neurodegenerative and other neurological disorders that affect an individual and their family for the rest of their lives. These conditions lead to substantial deterioration in QoL, require lifelong support from health and social care services, and often are an immense strain physically and emotionally on informal caregivers and family members.^6,8,9^ Health care costs increase in advanced disease and are higher in those most severely affected.^10^

There is a lack of robust evidence to support service and policy developments that improve palliative care provision for people with LTNCs.^11,12^ To date, there are 3 published small-scale pilot or phase 2 randomized clinical trials of palliative care interventions in neurological conditions.^13,14,15^ Findings of these trials reported slight improvements in symptom burden without harmful effects; however, they reported inconsistent effects on other outcomes (eg, QoL, caregiver burden). They also differed in key trial components, such as study population, intervention, outcome measures, and economic perspective.

Building on the phase 2 trial in multiple sclerosis (MS)^13^ and a longitudinal observational study in advanced atypical parkinsonism,^16^ we undertook this phase 3 trial to evaluate the clinical effectiveness and cost-effectiveness of short-term integrated palliative care (SIPC) among people severely affected by LTNCs, for improving symptoms and other patient and caregiver outcomes. Our primary null hypothesis was that there was no difference between study arms in their clinical effectiveness.

## Methods

### Study Design and Setting

Pragmatic phase 3, multicenter, randomized clinical trial of the clinical effectiveness and cost-effectiveness of SIPC for people with advanced LTNCs. Patients were recruited from 7 national hospitals with both neurology services and multiprofessional palliative care teams in the UK. Within study sites, a broad range of services were offered, including voluntary and National Health Services hospices, hospital and community based multidisciplinary palliative care, as well as tertiary and secondary neurological services.^5^ This trial and its protocol were approved by the London South East Research Ethics Committee. Patients and caregivers gave written informed consent. The trial protocol and intervention manual are available in Supplement 1. This study followed the Consolidated Standards of Reporting Trials (CONSORT) reporting guideline for randomized clinical trials..

### Participants

Patients severely affected by LTNCs and their caregivers were identified by a neurologist or a clinical nurse specialist and referred to the trial. The recruitment period was from April 1, 2015, to November 30, 2017, with a last follow-up date of May 31, 2018. Data were analyzed from November 2018 to March 2019.

For patients, inclusion criteria included (1) adults (aged ≥18 years) severely affected by advanced or progressive stages of 1 of the following: MS (usually Expanded Disability Status Scale score ≥7.5 [range, 0 indicates no disability in any functional system and 10 indicates death due to MS), all stages of motor neuron disease (MND), idiopathic Parkinson disease (IPD, Hoehn and Yahr stages 4-5), progressive supranuclear palsy (adapted Hoehn and Yahr stages 3-5) and multiple system atrophy (adapted Hoehn and Yahr stages 3-5); (2) an unresolved symptom which had not responded to standard care; and (3) at least 1 of the following: an unresolved other symptom; cognitive problems or complex psychological issues; communication or information problems or complex social need. Exclusion criteria included already receiving specialist palliative care, lacking mental capacity, and having no one available to advise on their behalf to provide proxy data.

For informal caregivers, inclusion criteria were adults identified by the patient as a person close to them who was able and willing to complete questionnaires. If the caregiver was unavailable or declined participation, only the patient was enrolled.

### Randomization and Masking

Randomization was undertaken independently by the UK Clinical Research Collaboration–registered King’s Clinical Trials Unit. Following patient consent and baseline data collection, randomization was performed in a 1:1 ratio, at the patient level with minimization for center, primary diagnosis (MS vs IPD vs progressive supranuclear palsy, multiple system atrophy and MND), and cognitive impairment (capacity vs impaired or lacking capacity). The data collectors (W.G. and I.J.H.) and the statistical and health economic analysis team (W.G., R.W., and D.Y.) were blinded to the group allocation until after the main analyses were completed and reported to the study steering committee.

### SIPC Intervention

SIPC focused on a comprehensive assessment, personalized care planning, case management and care coordination, and advising existing care providers. It was developed and evaluated using the Medical Research Council framework for evaluating complex interventions. SIPC was delivered by existing multiprofessional palliative care teams, linked with local neurology services. All staff involved in the delivery of the intervention were provided with a standard manual (Supplement 1) and face-to-face training in advance of the trial commencing. The intervention manual described the core elements to be covered when assessing patients as part of SIPC as well as the minimum standards for capturing and reporting delivery of SIPC.

SIPC usually lasted from 6 to 8 weeks from referral. Following referral, a key worker contacted the patient within 2 working days to arrange a visit within the next 5 working days. At this initial visit, a comprehensive palliative care assessment was undertaken considering both patient and caregiver and family needs. A problem list was generated, and a proposed care plan was developed to which the patient and their family agreed. The second contact (face-to-face or telephone) normally occurred within 2 weeks of the first visit to review progress with the care plan. The final contact involved a review of outcomes from actions already taken before discharge to local services as appropriate. The control arm continued to receive usual care services until after 12-weeks at which point they were referred to SIPC. A summary of the intervention is presented in Table 1.

**Table 1.** Details of SIPC and Standard Care

Timeline	SIPC	Standard care
Consent and baseline interview	Baseline research interview and consent before randomization	Baseline research interview and consent before randomization
Randomization		
2 working days from receiving referral	Palliative care assessment within 2 working days	NA
Weeks		
1-6	Palliative care, including assessment, treatment, referral, review	NA
6	Research interview 6 weeks post randomization.	Research interview 6 weeks postrandomization
6-8	Palliative care continues lasting 6-8 weeks, with referral on for those needing long-term care	NA
12-weeks (primary end point)	Research interview 12 weeks postrandomization	Research interview 12 weeks postrandomization
2 working days from receiving referral (following completion of 12-week research interview)	NA	Standard care group now offered palliative care within 2 working days of receiving referral
Weeks		
12-18	Discharge from palliative care team (if referred to community team, this continues from this point)	Palliative care, including assessment, treatment, referral, review
18	Research interview 18 weeks postrandomization	Research interview 18 weeks post randomization
18-20	NA	Palliative care continues lasting 6 to 8 weeks, with referral on for those needing long-term care
24	Final research interview 24 weeks postrandomization	Final research interview 24 weeks postrandomization
24-26	NA	Discharge from palliative care team (if referred to community team, this continues from this point)

Abbreviations: NA, not applicable; SIPC, short-term integrated palliative care.

### Data Collection

Data were collected at baseline and then 6-weekly until 24-weeks post randomization. Trained research nurses/researchers assisted as required in self-completion of patient and caregiver questionnaires according to the standardized schedule during their face-to-face visits. Ethnicity was self-defined by participants. We collected this data as it is a known confounder for accessing health care services. Caregivers usually self-completed their questionnaires during the patient interview. For adults lacking capacity, baseline and outcome measures were obtained from the informal caregiver. The qualitative study comprised interviews with patients and caregivers after SIPC was completed.

### Outcomes

The outcome measures used in this study are presented in eTable 1 in Supplement 2. The primary outcome was the change score between baseline and at 12-weeks in 8 symptoms (pain, shortness of breath, nausea, vomiting, constipation, spasms, difficulty sleeping, and mouth problems) as measured by the Integrated Palliative care Outcome Scale for Neurological conditions (IPOS Neuro-S8; each item was rated on a 5-point Likert scale in which 0 indicates no problem and 4 indicates an overwhelming problem; total score ranges from 0-32).^17^ Secondary outcomes for patients were changes in other palliative care symptoms, palliative care needs, psychological stress, health-related QoL and satisfaction. For caregivers, outcomes included caregiver burden and positivity as well as satisfaction. Patient-reported health service use was collected using the Client Services Receipt Inventory.^18^ For patients unable to convey outcomes, we collected caregiver’s assessment of patient’s problems and service use. As safety measures, we monitored serious adverse events, adverse events, and survival.

### Data Analysis

Given 80% power and 2-tailed significance of 5%, it was estimated that we needed to recruit 356 patients (178 per arm) to detect a small to medium effect size of 0.3 in the primary outcome, equivalent to a score change of 1.0 in the IPOS Neuro-S8 from baseline to 12 weeks postrandomization. The minimal clinically important difference in IPOS Neuro-S8 as estimated by a third of the standard deviation was 1.1.^17^ This estimation assumed a correlation coefficient of 0.4 between baseline and 12-week scores and 17% attrition. The parameters for sample size estimation were from previous studies.^13,16^

This paper reports baseline and at 12-week data only. Missing data were summarized according to the Methods of Researching End of Life Care classification.^19^ The mechanism of missing was assumed missing at random. Multiple imputation using chained equations was used to impute missing data. Twenty copies of imputed values were generated for each variable with missing data.

For baseline and outcome data, observations with complete data at both times were reported. Continuous variables were summarized using mean (standard deviation) and median (range) as appropriate. Categorical variables were reported as frequency counts and percentage.

Intention-to-treat analysis was carried out using generalized linear mixed model with center modeled as a random effect, adjusting for baseline score. The mean change scores from baseline to 12 weeks, effect sizes and their 95% CIs were reported. Effect sizes were calculated from model-based point estimates (95% CIs) of effect for individual outcomes divided by respective SDs, derived from standard errors using the method described previously.^20^ Survival was compared using the 2-sample *t* test and adverse events were compared using the χ^2^ test.

Six sensitivity analyses were conducted to assess the robustness of the findings from the main analysis: (1) the comparison between arms also accounted for the difference in ethnicity as there were more White individuals in SIPC than in the control arm (94.3% vs 86.2%; *P* = .009); (2) the 2 participants who were deemed ineligible postrandomization were excluded, thus the sample size for this analysis was 348; (3) we assessed differences in change scores between trial arms in complete patient data; (4) we assessed differences in change scores between trial arms in caregiver data; (5) we used complete patient data, if available at both baseline and week 12, and imputed proxy caregiver data if not; and (6) we evaluated the primary and secondary outcomes for patients with MS only to compare the effects of SIPC in this trial with that of the Phase 2 MS trial.^13^

The interaction effect between treatment and trial center on outcomes was explored by including a product term in the generalized linear mixed models. The *P* values were examined.

To determine the cost of SIPC, a base case incremental analysis from an NHS perspective was conducted. We valued health and social care use from the Client Services Receipt Inventory for the past 3 months at baseline and at 12 weeks by multiplying use by specific unit cost data from standard sources (eTable 9 in Supplement 2).

All significance tests were 2-sided at the level of .05 (primary and cost outcomes) or 0.0045 (secondary outcomes, Bonferroni correction to control for multiple testing 0.05 divided by 11 [total number of tests on the secondary outcomes]). Statistical analyses were performed in parallel using SAS, version 9.4 (SAS Institute Inc) and Stata, version 14 (StataCorp Inc).

Qualitative interviews were digitally recorded, transcribed verbatim, and anonymized prior to analysis. The data were analyzed thematically using the Coffey and Atkinson iterative approach.^21^

## Results

A total of 535 participants were assessed for eligibility. Among the 492 participants who met eligibility criteria, we recruited and randomized 350 patients and 229 caregivers across 7 centers, with 176 patients randomized to SIPC and 174 patients randomized to standard care (Figure). Patients’ mean (SD) age was 67 (12), and 179 (51%) were men (Table 2). Multiple sclerosis (N = 148) and IPD (N = 140) were the 2 most common diagnosed conditions. Patients had been living with their conditions for a median of 12 years (range, 0-56 years), 39 patients (11%) had cognitive impairment and approximately 60% of patients required either considerable assistance or total care for daily living.

**Figure.**
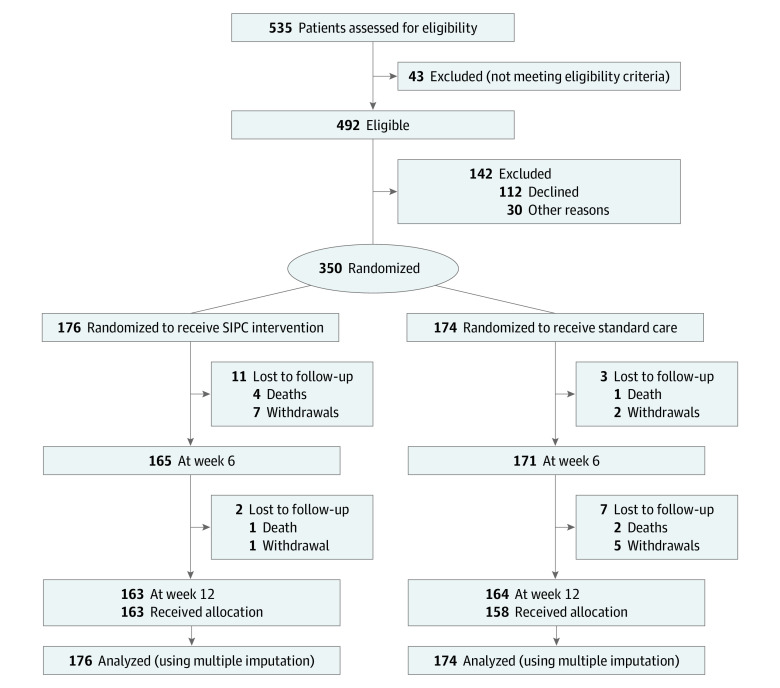
Consort Diagram Showing the Flow of Patients in OPTCARE Neuro Trial

**Table 2.** Demographic and Clinical Characteristics of Patients and Caregivers

Variable	No. (%)			
	Patients		Caregivers	
	SIPC	Standard C	SIPC	Standard C
No.	176	174	121	108
Age, mean (SD), y	67.3 (10.9)	66.4 (12.6)	63.3 (13.3)	65.3 (13.4)
Sex				
Male	86 (48.9)	93 (53.5)	41 (33.9)	40 (37.0)
Female	90 (51.1)	81 (46.6)	80 (66.1)	68 (63.0)
Marital status				
Single	16 (9.1)	19 (10.9)	7 (5.8)	7 (6.5)
Widowed	19 (10.8)	19 (10.9)	4 (3.3)	6 (5.6)
Married/civil partner	114 (64.8)	117 (67.2)	109 (90.1)	91 (84.3)
Divorced/separated	26 (14.8)	18 (10.3)	1 (0.8)	4 (3.7)
Not done/unknown	1 (0.6)	1 (0.6)	NA	NA
Living status				
Alone	35 (19.9)	30 (17.2)	4 (3.3)	5 (4.6)
Spouse/partner and/or children	125 (71.0)	119 (68.4)	109 (90.1)	91 (84.3)
Friend(s)/with others	16 (9.1)	25 (14.4)	8 (6.6)	12 (11.1)
Education				
No formal education	67 (38.1)	72 (41.4)	51 (42.2)	45 (41.7)
Upper secondary to postsecondary vocational qualification	53 (30.1)	63 (36.2)	37 (30.6)	29 (26.9)
Tertiary education	55 (31.3)	36 (20.7)	30 (24.8)	32 (29.6)
Not done/missing	1 (0.6)	3 (1.7)	3 (2.5)	2 (1.9)
Race/ethnicity				
White	166 (94.3)	150 (86.2)	113 (93.4)	98 (90.7)
Other ethnic group^a^	9 (5.1)	23 (13.2)	8 (6.6)	10 (9.3)
Employment				
No	173 (98.3)	167 (96.0)	86 (71.1)	76 (70.4)
Yes	3 (1.7)	7 (4.0)	35 (28.9)	32 (29.6)
Relationship to patient				
Spouse/partner	NA	NA	97 (80.2)	80 (74.1)
Son/daughter	NA	NA	17 (14.1)	12 (11.1)
Other	NA	NA	0	4 (3.7)
Having illness				
Yes	NA	NA	70 (57.9)	70 (64.8)
No	NA	NA	41 (33.9)	36 (33.3)
Feelings on present income				
Living comfortably	58 (33.0)	60 (34.5)	NA	NA
Coping	85 (48.3)	77 (44.3)	NA	NA
Difficult	12 (6.8)	12 (6.9)	NA	NA
Very difficult	7 (4.0)	7 (4.0)	NA	NA
Not done/unknown	14 (8.0)	18 (10.3)	NA	NA
Diagnosis				
Multiple sclerosis	74 (42.1)	74 (42.5)	NA	NA
Idiopathic Parkinson disease	71 (40.3)	69 (39.7)	NA	NA
Multiple system atrophy	7 (4.0)	5 (2.9)	NA	NA
Progressive supranuclear palsy^b^	13 (7.4)	14 (8.1)	NA	NA
Motor neuron disease	11 (6.3)	12 (6.9)	NA	NA
Time since diagnosis, median (range), y	12.3 (0-56)	12.4 (0-46)	NA	NA
Comorbidities				
Yes	134 (76.1)	117 (67.2)	NA	NA
No	42 (23.9)	57 (32.8)	NA	NA
Consent				
Patient consent	157 (89.2)	154 (88.5)	NA	NA
Personal consultee assent	19 (10.8)	20 (11.5)	NA	NA
Functional status as measured by AKPS^c^				
Totally bedfast	3 (1.7)	4 (2.3)	NA	NA
Almost completely bedfast	5 (2.8)	5 (2.9)	NA	NA
In bed >50% of the time	10 (5.7)	11 (6.3)	NA	NA
Requires considerable assistance	77 (43.8)	93 (53.5)	NA	NA
Requires occasional assistance	54 (30.7)	44 (25.3)	NA	NA
Cares for self	19 (10.8)	14 (8.1)	NA	NA
Normal activity with effort	7 (4.0)	2 (1.2)	NA	NA
Not available/applicable/not done	1 (0.6)	1 (0.6)	NA	NA

Abbreviations: NA, not applicable; SIPC, short-term integrated palliative care.

^a^ Other includes mixed/multiple ethnic groups, Asian/Asian British, Black/African/Caribbean/Black British, other ethnic group, don't know, prefer not to say, not available or not applicable, not done, or unknown.

^b^ Includes N = 2 patients with Corticobasal Degeneration.

^c^ The Australia-modified Karnofsky Performance Scale (100 – Normal to 0 – Dead).

### Primary and Secondary Clinical Outcomes

There were no statistically significant differences between trial arms for the primary outcome (effect size, −0.16; 95% CI, −0.37 to 0.05) or any of the secondary outcomes (effect size range, −0.20 to 0.12; *P* value range, 0.06 - 0.90). There was a small but statistically significant reduction in symptom burden at 12 weeks in the SIPC group (IPOS Neuro-S8, −0.78; 95%CI, −1.29 to −0.26) (Table 3). Most other patient outcomes were consistent: score changes in the control arm had either a smaller but statistically insignificant improvement or a greater decrease than the corresponding figures in the SIPC group. Results of the sensitivity analyses confirmed the robustness of the findings (eTables 2-7 in Supplement 2). None of the interaction between treatment and site on primary outcome or secondary outcomes was statistically significant. Neither adverse events nor survival was statistically different between the 2 groups

**Table 3.** Results of Primary and Secondary^**a**^ Outcomes Using Multiply Imputed Data From All Recruited Patients

Measure	SEM (95% CI)			*P* value^b^
	SIPC (N = 176)	Standard care (N = 174)	Effect size	
Primary outcome				
IPOS Neuro-S8				
Baseline	6.89 (6.24 to 7.54)	6.96 (6.34 to 7.58)	NA	NA
12-wk	6.11 (5.46 to 6.77)	6.68 (6.02 to 7.34)	NA	NA
Change score	−0.78 (−1.29 to −0.26)	−0.28 (−0.82 to 0.26)	−0.16 (−0.37 to 0.05)	.14
Secondary patient outcome				
IPOS Neuro-S24				
Baseline	26.69 (24.23 to 29.15)	27.16 (24.57 to 29.75)	NA	NA
12-wk	24.74 (22.10 to 27.37)	26.27 (23.58 to 28.96)	NA	NA
Change score	−1.95 (−4.38 to 0.48)	−0.89 (−3.15 to 1.36)	−0.13 (−0.34 to 0.08)	.22
IPOS Neuro 8				
Baseline	11.43 (10.07 to 12.79)	11.58 (10.09 to 13.08)	NA	NA
12-wk	10.59 (9.09 to 12.09)	11.80 (10.34 to 13.26)	NA	NA
Change score	−0.84 (−2.09 to 0.40)	0.21 (−1.25 to 1.68)	−0.20 (−0.41 to 0.01)	.06
IPOS Neuro				
Baseline	47.36 (41.94 to 52.78)	46.72 (40.93 to 52.51)	NA	NA
12-wk	43.14 (35.28 to 51.00)	44.22 (37.55 to 50.89)	NA	NA
Change score	−4.22 (−10.87 to 2.43)	−2.50 (−8.37 to 3.37)	−0.07 (−0.28 to 0.15)	.53
HADS anxiety				
Baseline	7.78 (6.78 to 8.77)	7.51 (6.52 to 8.50)	NA	NA
12-wk	7.43 (6.28 to 8.58)	7.59 (6.53 to 8.66)	NA	NA
Change score	−0.35 (−1.12 to 0.43)	0.08 (−0.65 to 0.81)	−0.12 (−0.33 to 0.09)	.27
HADS depression				
Baseline	8.13 (7.29 to 8.97)	8.31 (7.47 to 9.16)	NA	NA
12-wk	7.96 (7.03 to 8.88)	8.22 (7.35 to 9.09)	NA	NA
Change score	−0.17 (−0.79 to 0.45)	−0.09 (−0.78 to 0.59)	−0.04 (−0.25 to 0.17)	.69
EQ-5D VAS				
Baseline	52.72 (47.91 to 57.53)	52.25 (47.01 to 57.49)	NA	NA
12-wk	53.69 (48.03 to 59.34)	50.75 (45.36 to 56.14)	NA	NA
Change score	0.97 (−5.01 to 6.94)	−1.50 (−8.05 to 5.05)	0.12 (−0.09 to 0.33)	.27
SEMCD				
Baseline	5.39 (4.89 to 5.89)	5.13 (4.63 to 5.64)	NA	NA
12-wk	5.28 (4.75 to 5.82)	4.94 (4.41 to 5.47)	NA	NA
Change score	−0.10 (−0.60 to 0.40)	−0.19 (−0.70 to 0.31)	0.10 (−0.11 to 0.31)	.37
FAMCARE P16 (patient version)				
Baseline	50.33 (46.66 to 54.00)	50.30 (47.08 to 53.53)	NA	NA
12-wk	48.08 (43.75 to 52.41)	47.41 (43.52 to 51.31)	NA	NA
Change score	−2.26 (−6.05 to 1.53)	−2.89 (−6.23 to 0.45)	0.04 (−0.17 to 0.25)	.70
Secondary caregiver outcome				
ZBI 12				
Baseline	18.25 (15.59 to 20.90)	18.68 (16.28 to 21.08)	NA	NA
12-wk	18.60 (15.93 to 21.27)	18.92 (16.28 to 21.55)	NA	NA
Change score	0.35 (−0.98 to 1.68)	0.24 (−1.15 to 1.64)	0.01 (−0.20 to 0.22)	.90
ZBI Positivity				
Baseline	18.97 (17.36 to 20.59)	18.72 (17.05 to 20.38)	NA	NA
12-wk	18.87 (17.08 to 20.67)	18.12 (16.15 to 20.10)	NA	NA
Change score	−0.10 (−1.43 to 1.23)	−0.59 (−1.98 to 0.79)	0.09 (−0.12 to 0.30)	.40
FAMCARE 2 (carer version)				
Baseline	53.81 (49.64 to 57.97)	53.98 (49.93 to 58.02)	NA	NA
12-wk	53.99 (48.92 to 59.07)	53.23 (48.38 to 58.07)	NA	NA
Change score	0.19 (−4.86 to 5.23)	−0.75 (−4.64 to 3.14)	0.05 (−0.17 to 0.26)	.67

Abbreviations: EQ-5D VAS, EuroQoL 5-dimension visual analogue scale; HADS, Hospital Anxiety and Depression Scale; IPOS Neuro-S8, Integrated Palliative care Outcome Scale for Neurological conditions (contains 8 key symptoms); IPOS Neuro-S24, Integrated Palliative care Outcome Scale for Neurological conditions (contains 24 physical symptoms); NA, not applicable; SEM, standard error of the mean; SEMCD, Self-efficacy for Managing Chronic Disease; and ZBI, Zarit Burden Interview.

^a^ 95% confidence interval for primary outcome; 99.55% confidence intervals for secondary outcomes, Bonferroni correction to control for multiple testing (adjusted α = 0.0045,0.05/11).

^b^ *P* value for 2 group comparisons using generalized linear mixed model, adjusting for baseline score with center modeled as a random effect.

### Adverse Events and Survival Outcome

There were 5 deaths, 13 hospitalizations, and 2 emergency department visits up to 12 weeks in the SIPC group. The corresponding figures for the control arm were 3 deaths, 12 hospitalizations, and 5 emergency department visits up to 12 weeks. Survival between the 2 groups was comparable (11.6 vs 11.8 weeks). Neither adverse events (*P* = .61) nor survival (*P* = .28) was statistically different between the 2 groups.

### Health Economic Outcomes

There was a decrease in mean (95% CI) health and social care costs from baseline to 12 weeks −$1367 (95% CI, −$2450 to −$282) in the SIPC group and −653 (95% CI, −$1839 to −$532) in the control group (eTable 10 in Supplement 2). No significant differences were found between groups for the change scores of EuroQoL 5-dimension index score (0.04; 95% CI, −0.02 to 0.09; *P* = .08).

### Results of Qualitative Analysis

Patients (N = 26) and caregivers (N = 16) participated in 26 qualitative interviews from 3 trial centers (London, Brighton, Ashford-Surrey). The characteristics of participants are shown in eTable 11-12 in Supplement 2. Similar to the main sample, patients mostly had MS (69%) and had lived with their condition a median of 11 years. The themes of SIPC assessed by patients and caregivers included adapting to losses and building resilience, attending to function, deficits and maintaining stability, and enabling carers to care (eTable 13 in Supplement 2).

## Discussion

In this randomized clinical trial of palliative care in people with LTNCs, none of the evaluated outcomes were significantly different between the 2 groups, nor were adverse events, survival, or withdrawals. However, we found a small and statistically significant reduction in both the primary outcome and care costs in the intervention group. Health economic analyses suggested that SIPC may deliver better outcomes at a lower cost than standard care.

The heterogeneity of the disorders studied may have been factors in the non-significant results of the trial. IPD, MND, and MS differ in pathophysiology, clinical profiles, natural history as well as endophenotypes. Many consider IPD itself to be a syndrome^23^ and subtypes within MND and MS are also well recognized. Aspects related to non-motor symptoms of these disorders, particularly IPD, may also be substantially different from MS and MND, and may lead to the data being difficult to capture.^24,25^ Variations across centers, for example how the eligibility criteria were applied to recruit patients (eTable 8 in Supplement 2), the way the intervention was organized and delivered,^5^ may also have played a role in the intervention effect. Although the sample size was inflated to account for the heterogeneity when planning the study and training had been provided to ensure the consistency of key trial elements, it appeared that the heterogeneity was largely underestimated. The observed effect of SIPC in this trial was overall much smaller than that of the phase 2 trial (−0.14 vs −0.80).^26^

Outcome measures may be another factor in the interpretation of the intervention effects. The primary outcome was selected based on the 5 symptoms that were most responsive to the palliative care intervention in the phase 2 trial and additional symptoms from a longitudinal observational study of advanced Parkinson disease and atypical parkinsonism.^13,16^ Preliminary psychometric evaluations found that the measure exhibited good psychometric properties.^17,27^ In this trial, several items of the IPOS Neuro-S8 as well as the IPOS Neuro-S24 showed floor effects at baseline. However, per the eligibility criteria, the patients recruited should have unresolved symptoms, which the referring clinicians deemed to require input from specialist palliative care, especially in non-motor symptoms. Not all symptoms may be adequately captured by our outcome measures; therefore, further refinement of the outcome measures may be necessary.

### Limitations

This study has limitations. The sample was largely composed of patients with MS and IPD who tend to have a longer disease course. It is possible that the baseline symptom profiles and therefore the subsequent experience of SIPC are different for patients with LTNCs with a more rapid progression. Although every effort was made to standardize SIPC and our fidelity data showed that the intervention overall managed well (eTable 14 in Supplement 2), there were differences across centers. The intraclass correlation coefficient of 0.12 for IPOS Neuro-S8 was high but was not accounted for in our sample size estimation. The intervention teams were a mix of hospice based and hospital based, which led to differences in the make-up of their multidisciplinary teams as well as the services they were able to offer. There may have been contamination whereby participants in the control arm received components of SIPC.

## Conclusions

In this randomized clinical trial of SIPC vs standard care, there were no differences in patient reported outcomes or adverse events. Refining referral criteria to better match patients to SIPC and intervention optimization may help to support wider implementation of this new care model in practice.

## Notes

Supplement 1. **Trial Protocol and Intervention Manual**

Supplement 2. **eTable 1.** Outcome measures used in OPTCARE Neuro trial **eTable 2.** Means and Change Scores of Primary (95% CIs) and Secondary Outcomes (99.55% CIs) at Baseline and 12-Weeks Post Randomisation by Trial Arm, Using Multiply Imputed Data From All Recruited Patients **eTable 3.** Means and Change Scores of Primary (95% CIs) and Secondary Outcomes (99.55% CIs) at Baseline and 12-Weeks Post Randomisation, by Trial Arm, Using Multiply Imputed Patient Data Excluding Two Ineligible Patients **eTable 4.** Means and Change Scores of Primary (95% CIs) and Secondary Outcomes (99·55% CI) at Baseline and 12-Weeks Post Randomisation, by Trial Arm, Using Complete Patient Data at Both Baseline and 12-Weeks **eTable 5.** Means and Change Scores of Primary (95% CIs) and Secondary Outcomes (99·55% CI) at Baseline and 12-Weeks post Randomisation, by Trial Arm, Using Carers With Complete Data at Both Baseline and 12-Weeks **eTable 6.** Means and Change Scores of Primary (95% CIs) and Secondary Outcomes (99·55% CI) at Baseline and 12-Weeks Post Randomisation, by Trial Arm, Using Patients With Complete Data at Both Baseline and 12-Weeks Plus Imputed Proxy Data if Available at Both Baseline and 12-Weeks **eTable 7.** Means and Change Scores of Primary (95% CIs) and Secondary Outcomes (99·55% CI) at Baseline and 12-Weeks Post Randomisation by Trial Arm, Using Multiply Imputed Data From Patients With Multiple Sclerosis Only **eTable 8.** Participant Characteristics by Study Site **eTable 9.** Unit Costs of Health and Social Care Services in the Analysis **eTable 10.** Average Care Costs per Person (95% Confidence Intervals) at Baseline and 12 Weeks Post Randomisation, and Changes in Health and Social Care Costs by Trial **eTable 11.** Patient Demographics for Qualitative Interview Participants **eTable 12.** Carer Demographics for Qualitative Interview Participants **eTable 13.** Analytic Framework for the Qualitative Data on the Value and Impact of SIPC **eTable 14.** Analysis of Fidelity Data

Supplement 3. **Data Sharing Statement**
