# Supplementary material for: Effect of Short-term Integrated Palliative Care on Patient-Reported Outcomes Among Patients Severely Affected With Long-term Neurological Conditions: A Randomized Clinical Trial
Source: JAMA Netw Open. 2020 Aug 28;3(8):e2015061. doi: 10.1001/jamanetworkopen.2020.15061 (PMC7455856; doi:10.1001/jamanetworkopen.2020.15061)
Supplement: Supplement 1. — Trial Protocol and Intervention Manual [file jamanetwopen-3-e2015061-s001.pdf]

1. PROTOCOL FULL TITLE

Evaluation of the clinical and cost-effectiveness of Short-term Integrated Palliative Care Services (SIPC) to OPTimise CARE for people with advanced long-term Neurological conditions

Protocol Short Title/ Acronym: OPTCARE Neuro

Trial Identifiers

ISRCTN:	ISRCTN18337380		
REC Number:	14/LO/1765		
UKCRN Number:	18030		
Project Number	NIHR HS & DR - 12/130/47		
Protocol Version Number:	V3.0	Date:	28/08/2018

Sponsor

Name:	Professor Reza Razavi<br>Vice President & Vice-Principal (Research & Innovation)
	0207 188 4557
Email:	reza.razavi@kcl.ac.uk

Co-Sponsor

**Name:**	King's College Hospital
**Address:**	Research and Development Department, King's College Hospital NHS Foundation Trust, 161 Denmark Hill, London SE5 8EF
**Telephone:**	020 3299 1980
**Email:**	kch-tr.research@nhs.net

Confidentiality and Copyright: This document is confidential and property of *Cicely Saunders Institute, King's College London.* This document may not be copied, disclosed, distributed used or destroyed in whole or in part without the prior written consent of *Cicely Saunders Institute, King's College London*

2. Table of Contents

1. PROTOCOL FULL TITLE 1
2. TABLE OF CONTENTS 2
3. CHIEF INVESTIGATORS, CO-INVESTIGATORS, AND TRIAL STATISTICIAN 4
4. SITE PRINCIPAL INVESTIGATORS 5
5. COLLABORATORS 5
6. RESEARCH TEAM 6
7. PARTICIPATING SITES 7
8. TRIAL FLOWCHART 8
9. STUDY SYNOPSIS 9
10. PROTOCOL REVISION HISTORY 12
11. GLOSSARY OF TERMS 13
12. BACKGROUND & RATIONALE 14
13. TRIAL OBJECTIVES AND DESIGN 16
13.1 Trial Objectives 16
13.2 Trial Design 16
14. TRIAL INTERVENTION 17
14.1 Intervention Details 17
14.2 Frequency and duration of intervention 20
14.3 Intervention records 20
14.4 Subject Compliance & Adherence 20
14.5 Concomitant Medication 20
15. RESEARCH ENVIRONMENT 21
16. SELECTION AND WITHDRAWAL OF SUBJECTS 21
16.1 Inclusion Criteria 21
16.2 Exclusion Criteria 22
16.3 Selection of Participants 22
16.3.1 Raise awareness 22
16.3.2 The Mental Capacity Act 2005 23
16.3.3 Process of consent and assent for adults lacking capacity 24
16.3.4 Consent in the moment for participants with impaired capacity 24
16.3.5 Advance consent and assent for participants who lose capacity 24
16.3.6 Assent for adults lacking capacity 24
16.3.7 Documentation of study participation 24
16.4 Randomisation Procedure / Code Break 25
16.5 Withdrawal of Subjects 26
16.6 Expected Duration of Trial 26
17. TRIAL PROCEDURES AND DATA COLLECTION 26
17.1 Main data collection by visit 26
17.2 Research interviews 26
17.3 Distress Protocol 26
17.4 Qualitative component 27
17.5 Survey of health professionals 27
18. ASSESSMENT OF EFFICACY 29

18.1 PRIMARY EFFICACY PARAMETERS 29
18.2 SECONDARY EFFICACY PARAMETERS 29

19. ASSESSMENT OF SAFETY 32

19.1 SPECIFICATION, TIMING AND RECORDING OF SAFETY PARAMETERS. 32
19.2 PROCEDURES FOR RECORDING AND REPORTING ADVERSE EVENTS 32
19.3 STOPPING RULES 32

20. STATISTICS 32

20.1 SAMPLE SIZE 32
20.2 ANALYSIS 32
20.2.1 Quantitative analysis 32
20.2.2 Qualitative analysis, survey and integration of all data 33

21. STUDY STEERING COMMITTEE 34

22. DATA MONITORING AND ETHICS COMMITTEE 34

23. DIRECT ACCESS TO SOURCE DATA AND DOCUMENTS 34

24. ETHICS & REGULATORY APPROVALS 34

25. QUALITY ASSURANCE 34

26. DATA HANDLING AND MANAGEMENT 34

26.1 DATA HANDLING: 34
26.2 DATA MANAGEMENT: 35

27. PUBLICATION POLICY 36

28. INSURANCE / INDEMNITY 37

29. CONFIDENTIALITY AGREEMENT 37

30. REFERENCES 38

31. FINANCIAL ASPECTS 42

32. DECLARATION OF INTEREST 42

33. SIGNATURES 42

34. APPENDICES 43

34.1 MANAGEMENT STRUCTURE FOR THE PROJECT 43
34.2 PUBLICATIONS FROM PHASE II TRIAL AND RELATED STUDIES 44

3. Chief Investigators, Co-Investigators, and Trial Statistician

Name	Role on project	Expertise
Prof Irene J Higginson[1]	Chief Investigator	Palliative care evaluation, recruitment, complex interventions, public health, HSR, quality of life, project management
Dr Wei Gao[1]	Co-CI, Trial Statistician	Medical statistics and epidemiology in palliative and supportive care, project management, trial and healthcare service evaluations
Prof Ammar Al-Chalabi[11]	Co-applicant	Neurology, expertise in MND
Dr Cynthia Benz	Co-applicant, Patient Representative	A person with MS, with experience of the pilot trial
Dr Rachel Burman[3]	Co-applicant	Pain and symptom control, advance care planning, specialist palliative care assessments particularly neurodegenerative disorders, service design and patient recruitment
Dr Anthony Byrne[7]	Co-applicant	Expertise in the design and management of palliative care studies, recruitment
Prof Ray Chaudhuri[11, 12]	Co-applicant	Neurology, expertise in Parkinson’s disease
Dr Vincent Crosby[5]	Co-applicant, Co-lead intervention modelling, training and quality assurance	Clinical palliative care, interest in neurological conditions
Dr Catherine Evans[1, 13]	Co-applicant, Lead qualitative component	Nursing, mixed methods, synthesis of qualitative with quantitative outcome data
Prof Matthew Hotopf[9]	Co-applicant	Epidemiological psychiatry, psychiatry in advanced disease, trials of complex interventions
Dr Diana Jackson[1]	Co-applicant, Lead qualitative component	Caregiver support, rehabilitation, outcome measures, professions allied to medicine, LTNCs
Prof P Nigel Leigh[8]	Co-applicant	Neurological conditions, in particular MND and PP
Prof Paul McCrone[4]	Co-applicant	Health economics in neurological conditions, mental health and palliative care
Dr Fliss Murtagh[1]	Co-applicant	Palliative care, project management, outcome measurement, clinical trials, health psychology, managing psychological and spiritual distress
Prof Andrew Pickles[2]	Co-applicant	Biostatistics, psychological measurement, Director of the fully accredited King’s College CTU

Dr Eli Silber[3]	Co-applicant	Developed MS service, general neurology, clinical trials, developing services in MS and palliative care and mental health
Dr Andrew Wilcock[6]	Co-applicant	Trials, palliative care, long-term conditions beyond cancer
Prof Carolyn A Young[10]	Co-applicant	Neurology and clinical trials

4. Site Principal Investigators

Name	Site	Expertise
Dr Sabrina Bajwah	London	Consultant palliative medicine
Dr Anthony Byrne[7]	Cardiff	Design and management of palliative care studies, recruitment
Dr Vincent Crosby[5]	Nottingham	Clinical palliative care, interest in neurological conditions
Dr Fiona Lindsay[13]	Brighton	Consultant palliative medicine
Prof Carolyn A Young[10]	Liverpool	Neurology and clinical trials
Dr Clare Smith[16]	Ashford	Consultant palliative medicine
Dr Ellie Smith[17]	Sheffield	Consultant palliative medicine

5. Collaborators

Name	Role on project	Expertise
Ms Caroline Murphy[15]	Collaborator	Trial management, trial coordination, palliative care nurse
Prof Lynne Turner-Stokes[14]	Collaborator	Rehabilitation, long-term neurologocial conditions

6. Research team

Name	Role on project	Expertise
Dr Nilay Hepgul[1]	Project Manager / Research Associate (as of April 2015)	Project management, quantitative research, predictors of outcomes, mental health
Caty Pannell[3]	Senior Research nurse (until November 2016)	Training of research nurses, patient recruitment
Debbie Tonkin[1]	Project Administrator	Administrative support throughout the project
Dr Liesbeth van Vliet[1]	Project Manager / Research Associate (until April 2015)	Qualitative research, communication, outcome measures, patient perspective

Affliations:

1 King's College London, Cicely Saunders Institute, Department of Palliative Care, Policy and Rehabilitation, London, UK
2 King's College London, Institute of Psychiatry, Psychology & Neuroscience, Biostatistics Department, London, UK
3 King's College Hospital NHS Foundation Trust, London, UK
4 King's College London, Institute of Psychiatry, Psychology & Neuroscience, Health Services and Population Research Department, London, UK
5 Nottingham University Hospitals NHS Foundation Trust, Notthigham, UK
6 The University of Nottingham, Nottingham, UK
7 Cardiff and Vale University Health Board, Cardiff, UK
8 University of Sussex, Brighton and Sussex Medical School, Trafford Centre for Biomedical Research, Brighton, UK
9 King's College London, Institute of Psychiatry, Psychology & Neuroscience, Department of Psychological Medicine, London, UK
10 The Walton Centre NHS Foundation Trust, Liverpool, UK
11 King's College London, Institute of Psychiatry, Psychology & Neuroscience, Department of Clinical Neuroscience, London, UK
12 King's College London, National Parkinson Foundation International Centre of Excellence, London, UK
13 Sussex Community NHS Foundation trust
14 Regional Rehabilitation Unit, Northwick Park Hospital, London, UK
15 King's College London, Institute of Psychiatry, Psychology & Neuroscience, Clinical Trials Unit, London, UK
16 Ashford & St. Peter's Hospitals NHS Foundation Trust, Chertsey, UK
17 Sheffield Teaching Hospitals NHS Foundation Trust, Sheffield, UK

Contact: Nilay Hepgul, **Tel**: 020 7848 5805, **Email**: optcareneuro@kcl.ac.uk

7. Participating sites

We have been listed on the NIHR CRN portfolio. For a limited period we will make the trial ‘open to new sites’, who may wish to take part. We will consider such sites if they can ensure sufficient local infrastructure to take part in the central training, deliver the intervention and ensure recruitment and robust data collection. We have already had interest from such sites. We will conduct the prior mapping in these sites to ensure baseline information and to help assess whether they can be included in the study.

**Site**	**Number**
King’s College Hospital NHS Foundation Trust, London	01
The Walton Centre NHS Foundation Trust, Liverpool	02
Cardiff & Vale University Health Board, Wales	03
Nottingham University Hospitals NHS Trust / University of Nottingham	04
Brighton & Sussex University Hospitals NHS Trust / Sussex Community Foundation NHS Trust	05
Ashford & St. Peter’s Hospitals NHS Foundation Trust	06
Sheffield Teaching Hospital NHS Foundation Trust	07

8. Trial Flowchart

Title: Evaluation of the clinical and cost-effectiveness of Short-term Integrated Palliative Care Services (SIPC) to OPTimise CARE for people with advanced long-term Neurological conditions (LTNCs)

Development, Set up and Trial commencement with assessment of feasibility
Month 1-9: mapping, feasibility, qualitative exploration. Month 12: trial commences (monthly review). Month 14: formal review.

Setting
Five + centres with neurology, palliative care and rehabilitation services in South London, Brighton, Cardiff, Liverpool and Nottingham

Population-recruitment
Patients severely affected by long-term neurological condition (MS, PRDs, MND) and deemed (by clinicians) to be optimally managed yet have an unresolved symptom, and at least one of the following: unresolved other symptom, cognitive problems, complex psychological or social needs. Living in catchment area of SIPC. The nearest caregiver.

Excluded
Not meeting inclusion criteria (patient receiving palliative care currently or in the last 6 months; patient lacking capacity and no caregiver to complete questionnaires)

Baseline interview

- Participant baseline demographics, clinical details, cognitive impairment and neurological disabilities
- Participant completed questionnaires – e.g. IPOS Neuro-S8, EQ5D, ICECAP-A, HADS, CSRI
- Caregiver details and caregiver questionnaires – e.g. VR-12, ZBI-12 + positivity

Randomisation with minimization (with random element) stratified by: centre, disease and cognitive impairment

Standard Care (Control/Delayed intervention arm, n=178)
After randomisation, patients will continue their usual services and treatments.
After the second follow-up interview (approximately 12 weeks), they will be offered the SIPC and followed up to the end of the study.

Standard Care + SIPC (Fast-track Intervention arm, n=178)
SIPC will be offered to patients immediately after randomisation in addition to their usual services and treatments (Standard Care). SIPC will be delivered through existing Multiprofessional Palliative Care Teams (MPCTs), linked with local neurology and rehabilitation services. Following referral, a key worker will undertake a comprehensive palliative care assessment on the patient within 5 working days. In keeping with the multi-disciplinary ethos of palliative care, the detailed assessment is discussed with the MPCT who: suggest ways to improve management of physical, emotional, social and other problems, provide specialist welfare benefits advice, help with advance care planning, liaises with and acts as a catalyst with local health services (primary and specialist teams, and social care).

Follow-up at 6, 12*, 18 and 24 Weeks (* patients in control arm are offered SIPC)
Primary Outcome
Symptom reduction at 12 weeks measured by the Integrated Palliative care Outcome Scale for neurological conditions (IPOS Neuro-S8)

Main secondary Outcomes
1) Patients' palliative needs and symptoms: measured by the IPOS Neuro;
2) Patients' health-related quality of life and well-being: measured by the EQ-5D and ICECAP-A;
3) Patients' psychological distress: measured by the 14 item Hospital Anxiety and Depression Scale (HADS);
4) Patients' satisfaction, self-efficacy and other aspects: measured by the modified FAMCARE scale (FAMCARE-P16), the Self-Efficacy to Manage Chronic Disease Scale (SEMCD), an advance care planning scale additional questions covering patients' experiences of the study;
5) Hospital and service use, recorded using an adapted version of the Client Service Receipt Inventory (CSRI), and patient survival, as assessed in days from consent;
6) Caregiver burden and quality of life: measured by the 12 item Zarit Burden Inventory (ZBI-12), the VR-12 and the modified FAMCARE scale (FAMCARE 2);
7) Economic evaluations (cost-effectiveness): as measured by quality adjusted life years using the EQ-5D and ICECAP-A;
8) Comparison of effects to test timing of referral and how the effect changes over time

- Continue recruitment in trial (to month 33)
- Continue follow up interviews (to month 36 for primary outcome analysis)
- Continue follow up interviews and extraction from clinical records (to month 39 for secondary outcome analysis)
- Qualitative interviews with 6 patients and caregivers per centre who received SIPC

9. Study Synopsis

**TITLE OF CLINICAL TRIAL:**	Evaluation of the clinical and cost-effectiveness of Short-term Integrated Palliative Care Services (SIPC) to OPTimise CARE for people with advanced long-term Neurological conditions
**Protocol Short Title/ Acronym:**	OPTCARE Neuro
**Study Phase If Not Mentioned In Title:**	Phase III
**Sponsor Name:**	King’s College London
**Chief Investigator:**	Professor Irene J Higginson
**UKCRN Number:**	18030
**REC Number:**	14/LO/1765
**Medical Condition Or Disease Under Investigation:**	Long-term neurological conditions
**Purpose Of Clinical Trial:**	To determine the effectiveness and cost-effectiveness of Short-term Integrated Palliative Care Services (SIPC) in improving symptoms, selected patient and caregiver reported outcomes and in reducing hospital utilisation for people severely affected by long-term neurological conditions (LTNCs)
**Primary Objective:**	To determine the effectiveness of SIPC for people severely affected by LTNCs compared to standard care according to the primary outcome of reduction in key symptoms
**Secondary Objective(s):**	1. To map current practice and document the services available (and common care pathways) for patients with LTNCs and their caregivers/families in the areas of the study, to better understand variations in normal practice experienced by the control group;<br>2. To test the feasibility of offering SIPC and the trial methods across five centres for people severely affected by LTNCs and to modify the intervention and trial methods accordingly;<br>3. To determine the effectiveness of SIPC for people severely affected by LTNCs compared to standard care in the secondary outcomes: palliative care needs and other symptoms, patient psychological well-being and quality of life, caregiver burden/positivity and quality of life, patients and carers’ satisfaction and communication;<br>4. To determine the effects of SIPC for people severely affected by LTNCs on hospital admissions, length of hospital stay, emergency attendance and other service use over the trial period, patient survival;<br>5. To determine the cost-effectiveness of SIPC for people severely affected by LTNCs;<br>6. To understand how the change process may work and to identify components of the SIPC that are most valued by patients, their families/caregivers and other healthcare professionals;<br>7. To determine how the effects change over time, whether earlier referral to palliative care affects the subsequent

	response to palliative care, and when assessment or re-referral might be beneficial
**Trial Design:**	A randomised Phase III multicentre pragmatic fast-track controlled trial of a complex intervention, with an embedded qualitative component
**Endpoints:**	<u>Primary endpoint</u><br>A combined score of eight key symptoms, measured by symptom subscale of the Integrated Palliative care Outcome Scale for neurological conditions (IPOS Neuro-S8) at 12 weeks post randomisation (primary objective)<br><br><u>Secondary endpoints</u><br>1. Patients' other symptoms and palliative care needs: measured by the relevant symptom component of the IPOS Neuro<br>2. Patients' health-related quality of life and well-being: measured by the EQ-5D and ICECAP-A<br>3. Patients' psychological distress: measured by the 14 item Hospital Anxiety and Depression Scale (HADS), patient reported<br>4. Patients' satisfaction, self-efficacy and other aspects: measured by the modified FAMCARE scale (FAMCARE-P16), the Self-Efficacy to Manage Chronic Disease Scale (SEMCD), an advance care planning scale and some questions covering patients' experiences of the study<br>5. Hospital admissions, length of hospital stay, emergency attendance and other service use during the course of the study: measured by an adapted version of the Client Service Receipt Inventory (CSRI), and survival from consent (days)<br>6. Caregiver burden, positivity and quality of life: measured by the Zarit Burden Inventory (ZBI-12) (12 item version+8 positive items), the VR-12 and the modified FAMCARE scale (FAMCARE 2), as self-assessed by caregiver<br>7. Caregiver assessment of patients' outcomes: using the same measures (IPOS Neuro and CSRI) and used to aid imputation of missing patient data)<br>8. Observer (completed by the researcher) assessment of the patients' problems: measured by the Support Team Assessment Schedule (STAS)<br>The main endpoint is at 12 weeks before the standard care arm receiving the intervention.
**Sample Size:**	356, 178 in each arm (short-term integrated palliative care vs standard care)
**Summary Of Eligibility Criteria:**	Inclusion criteria:<br><u>Patients</u><br>1) Adults (aged 18 years or over) severely affected by advanced or progressive stages of the long-term neurological conditions (LTNCs) of either*:<br>• Multiple Sclerosis (MS) - patients with either aggressive relapsing disease with rapid development of fixed disability or those with advanced primary or

	secondary progressive disease, often with limitation in a number of areas including gait and upper limb function. We do not define referral based on disability but would expect most patients to have an Expanded Disability Status Scale (EDSS) of at least 7.5<br>• Parkinsonism & related disorders (PRDs) i.e.<br>o Idiopathic Parkinson's Disease (IPD) - Hoehn and Yahr (H&Y) stages 4-5 OR<br>o Progressive Supranuclear Palsy (PSP) - Hoehn and Yahr (H&Y) stages 3-5 OR<br>o Multiple System Atrophy (MSA) - Hoehn and Yahr (H&Y) stages 3-5<br>• Motor Neurone Disease (MND) all stages<br>AND<br>2) who are deemed (by referring/usual care clinicians) to have:<br>• an unresolved symptom (e.g. pain or another symptom) which has not responded to usual care<br>• AND at least one of the following: unresolved other symptom (e.g. breathlessness, nausea / vomiting, spasticity, fatigue); cognitive problems; complex psychological (depression, anxiety, loss, family concerns), communication/information problems and/or complex social needs<br>AND<br>3) who are able to give informed consent^ OR where their capacity can be enhanced^ (e.g. with information) so they can give informed consent OR where a personal consultee^ can be identified and approached to give a opinion on whether or not the patient would have wished to participate in the study<br><br>AND<br>4) are living in the catchment area of the Short-term Integrated Palliative Care Service (SIPC)<br><br>* Diagnosis must have been established by a specialist neurological assessment.<br><br>^ When a person lacks capacity to consent for themselves the procedures detailed in the Mental Capacity Act (2005) are adhered to<br><br>We expect patients to be in the advanced or progressive stages of disease. They may be living at home (most common), in a nursing home or in hospital at the time of recruitment. We will develop a proforma for referring clinicians to complete (covering contact and clinical information and important reasons for referral/selection).<br><br>Caregivers<br>1) Adults (aged 18 years or over) identified by the patient as the person closest to them, usually a family member, close friend, informal caregiver or neighbour<br>2) able to give informed consent to complete the questionnaires

	Exclusion criteria:<br>Patients who meet the inclusion criteria but:<br>1) are already receiving specialist palliative care currently or have done so in the last 6 months *(please note: seeing any palliative care specialist counts as exclusion criteria)*;<br><br>2) lack capacity and have no family member, friend or informal caregiver who is willing and available to complete questionnaires about their own and the patient's symptoms and circumstances
**Intervention (Description, frequency, details of delivery)**	Short-term Integrated Palliative Care (SIPC) offered to patients severely affected by long-term neurological conditions, lasting for 6-8 weeks from referral. SIPC will be delivered by existing multiprofessional palliative care teams (MPCT), linked with local neurology and rehabilitation services.
**Comparator Intervention:**	Standard care, the control arm is offered SIPC after the second follow-up interview is completed and reviewed
**Maximum Duration Of Treatment Of A Subject:**	6-8 weeks from referral. The trial is of a new service. The service visits patients usually on three occasions over a period of up to 6-8 weeks from first contact. The service would not usually stay involved longer than 12 weeks. However, up to 10% of patients may want and need on-going palliative care in which case the services will provide this.

10. Protocol Revision History

Version Number	Date
V1.0	16/09/2014
V1.1	21/11/2014
V2.0	06/05/2015
V3.0	28/08/2018

11. Glossary of terms

DMEC - Data Monitoring and Ethics Committee

IPD - Idiopathic Parkinson's Disease

IPOS - Integrated Palliative care Outcome Scale

LTNCs - Long-term Neurological Conditions

MCA - Mental Capacity Act

MND - Motor Neurone Disease

MPCT - Multidisciplinary Palliative Care Team

MRC - Medical Research Council

MS - Multiple Sclerosis

MSA - Multiple System Atrophy

NIHR - National Institute for Health Research

NSF - National Service Framework

PD - Parkinson's Disease

PRDs - Parkinsonism and related disorders (i.e. IPD, PSP, or MSA)

PSP - Progressive Supranuclear Palsy

QUALYs - Quality Adjusted Life Years

SIPC - Short-term Integrated Palliative Care

SSC - Study Steering Committee

12. Background & Rationale

Long-term conditions and long-term neurological conditions (LTNCs)

Over 15 million people have a long-term condition, and these account for about 70% of NHS spend; about half of all general practice activity; and two thirds of hospital outpatient activity [7]. However, the Department of Health has raised concerns that current services may not be sufficiently well organised to promote independence and provide the best quality care for patients. The current system is often characterised by silo working in primary and secondary care and reactive services. The trend towards greater specialisation and organisation differentiation may further disadvantage people with long-term conditions. Neurology has been overlooked within policy initiatives in England [8 9] despite the publication of a National Service Framework for long-term neurological conditions (LTNCs) [10]. In the UK, at any one time there are 130,000 people with Idiopathic Parkinson's Disease (IPD); a further 100,000 with Multiple Sclerosis (MS); 4,000 with Motor Neurone Disease (MND) and 4,000 with Progressive Supranuclear Palsy (PSP) or Multiple System Atrophy (MSA) [11 12]. More than one person in 50 over the age of 80 is affected by IPD, making it a very important disease for future ageing populations. MS is the most common form of disability for younger adults. All of these conditions lead to substantial deterioration in quality of life and require lifelong support from health and social care services [9 12-14].

Current NHS policy and practice

In 2005, the Department of Health published the National Service Framework (NSF) for long-term conditions which set out quality requirements for health and social services to improve the quality of life of people with long-term conditions and their carers [10]. The NSF was a key tool for delivering the government's strategy to support people with LTNCs. Each of the 11 quality requirements represented in the NSF has a specific aim and rationale for LTNCs. A key group of individuals for this strategic plan are individuals with long-term progressive neurological conditions such as MND, MS and Parkinsonism and related disorders (PRDs), comprising IPD, MSA and PSP. Affecting over 200,000 individuals in the UK, these conditions have in common patterns of impact on quality of life arising from wide-ranging physical deterioration and resulting disabilities [9]. Despite the progressive nature of these conditions, the scope for improving services to improve quality of life for individuals with these conditions may be substantial.

As with many other health systems, healthcare in the UK is based in general terms on a historical division between general practitioners, staff working in the community, and hospital-based specialists [15 16]. It is increasingly recognised that a hard separation of these functions does not meet the needs of those with chronic conditions. Attempts have been made to better coordinate care through integrative processes such as joint budgets, governance, information systems, flows of data or case management [17 18]. These may be brought together more formally, through different kinds of vertical integration, where agencies involved at different stages of the care pathway form part of a single organisation or function as well as horizontal integration of community-based services in examples such as health and social care teams for the frail elderly [19]. The short-term integrated palliative care (SIPC) proposed in this study could be seen as one example of this. Another issue is that multi-morbidity is the norm for people with chronic conditions. This is the case for the patient groups included in this study, where patients with PRDs, MND and MS may have other conditions which also affect their health and quality of life. Equally, their spouses or family caregivers may have health conditions that affect their ability to care, and the burden of caring may affect the health of caregivers [13 20]. Palliative care is person rather than disease focussed. Different forms of integrated care have been developed to shape services better for people with long-term conditions, and SIPC builds on these approaches.

Models of care to improve care for those severely affected by long-term neurological conditions

Systematic reviews on the evidence of palliative home care [21], support for caregivers [22], services for older people [23] and end of life care pathways [24] all identify a wealth of observational studies demonstrating need and little on effectiveness. A search of Pub Med, the Cochrane database, trial registers and the NIHR register has identified no phase III trials and only one phase II trial of palliative care (our study) [25] for patients with any neurological conditions during the more advanced stages of illness. There are only a handful of randomised trials of multidisciplinary palliative care teams (MPCTs) and rarely are health economic aspects included. These trials are from the USA where service configurations may be different. No trial considered patients with neurological diseases. A review of multidisciplinary care in MND concluded that "the 'best' evidence to date is based on three 'low' and two 'very low quality' observational studies" [26].

How this project will add to the body of knowledge

The target of this project is to improve care for those patients who are severely affected by long-term conditions, who are at the highest levels of need, as depicted using the 'Kaiser pyramid' or the NHS generic model of long-term conditions, i.e. the top segment (tier 1) with complex needs [[7]]. The Department of Health outlines the following interventions that can be used according to patient needs:

- Case management - dedicated one to one support from a highly skilled health professional (e.g. a community matron) with regular face to face contact.
- Personalised care planning - placing the person at the centre of decision making about their care and agreeing a plan of how that care will be delivered.
- Support people to self-care - providing people with information and skills to make day to day decisions about the way they manage their health. This has included developing the Expert Patients Programme.
- Assistive technology - using the emerging telecare and telehealth technology and telephone coaching arrangements to support people to remain independent and self-care for as long as possible.

SIPC uses a combination of these approaches and will add to the wider knowledge regarding new and innovative models of care for people with long-term conditions. This research will add to the evidence base by providing a successful randomised trial of the cost effectiveness of SIPC for people with neurological conditions (a neglected group).

Why this research is needed now

The UK Service Framework for LTNCs proposes that palliative care is considered for non-cancer patients earlier in the disease trajectory [[10]], but provides little information on how to do this. People affected by these neurological conditions, identified through relevant organisations, including the MS Society, and the DenDRon network, identified that palliative care needs are important and services should be developed to address unmet needs for symptom support. Non-motor symptoms of Parkinson’s Disease are the key determinant of quality of life and the main cause of mortality, hospitalisation and institutionalisation, yet remain under-treated, under-reported and a key unmet need [[27]]. SIPC in all these conditions would aim to address this unmet need. It is vital to undertake our trial now; otherwise there is a risk that interventions will be developed in response to this guidance and need, without appropriate evaluation and possibly not in the most cost effective way.

Following the Medical Research Council (MRC) guidance for the development and evaluation of complex interventions, modelling work showed that patients severely affected by LTNCs had symptom problems, psychosocial needs and their caregivers needed support, both emotional and in care co-ordination [[2 3]]. The results of our Phase II trial of SIPC among 52 people severely affected by MS, found the benefit of the new service in reducing symptoms and informal caregiving burden at a lower cost with no harmful effect, compared with the best available service [[28]]. We recently reported findings from a longitudinal observational study on PRDs which demonstrated the profound and complex mix of non-motor and motor symptoms in patients with late stage disease. Symptoms are not resolved and half of the patients deteriorate. Palliative problems are predictive of future symptoms, suggesting that an early palliative assessment might help screen for those in need of earlier intervention [[29]].

Since the completion of these studies, interest has grown in how the new SIPC service will perform when it is rolled out to more settings and more conditions. Whether more people living with LTNCs can benefit from the SIPC, and whether it can be routinely used in practice to improve the care quality for people severely affected by LTNCs, particularly in the current financial environment is of interest. If found effective, the new SIPC service has the potential to be beneficial for a wider range ofconditions and in more diverse care settings for patients and their families. This could result in better symptom control and improved quality of life for patients, as well as improved co-ordination of care, more efficient and appropriate use of services, and a reduction in the number of unnecessary emergency admissions at the end of life. This is also in line with other palliative care NHS initiatives, which are seeking to move palliative and supportive care, and discussions about preferences and priorities, further upstream – encouraging patients to think about care preferences earlier in their disease trajectory [[30]]. Understanding whether SIPC is clinically and cost effective, and its potential mechanism of action, will help to develop studies in these initiatives. Equally, if the SIPC is not cost effective in more conditions and in wider settings, the findings will prompt development of customised improvement and modifications in specific LTNCs [[31-34]]. Health care costs in the last year of life are high; 18-30% of healthcare spending, with resource use increasing in the last months of life [[31-33]]. In long-term conditions, including neurological conditions, costs rise with increased disability and as the disease advances [[34]]. These costs can be unpredictable and affect caregivers and patients, as well as health and social services [[34]]. Therefore, it is important to evaluate proposed service models in patients with advanced

disease to see if they can optimise symptom control and care, and reduce distress and costs. Evaluating a new service model for LTNCs, as well as addressing these diseases, develops a potential model of service provision for other long-term diseases in advanced stages.

SIPC could be developed with only small adaptions to existing health care services. It is much more likely to be possible than other proposed alternatives, such as developing long-term palliative care models. The latter would be difficult to achieve without considerably expanding the number of palliative care specialists, beds and services. In contrast, SIPC builds on and integrates with existing services across the UK, and seeks to empower patients, improve symptom control and integrate with existing services, improving their expertise. With the ageing of the population, the predicted rise in the annual number of deaths by 17% by 2030, and the increasing prevalence of long-term conditions, it is both highly relevant and timely to robustly test new service models to improve care for this group. This project answers this need and tests an intervention that could be implemented by the current workforce and services.

13. Trial Objectives and Design

13.1 Trial Objectives

Aim: To determine the effectiveness and cost-effectiveness of Short-term Integrated Palliative Care Services (SIPC) in improving symptoms, selected patient and caregiver reported outcomes and reducing hospital utilisation for people severely affected by long-term neurological conditions (LTNCs)

Primary objective:

To determine the effectiveness of SIPC for people severely affected by LTNCs compared to standard care according to the primary outcome of reduction in key symptoms at 12 weeks.

Secondary objectives

1. To map current practice and document the services available (and common care pathways) for patients with LTNCs and their caregivers/families in the areas of the study, to better understand variations in normal practice experienced by the control group;
2. To test the feasibility of offering SIPC and the trial methods across five centres for people severely affected by LTNCs and to modify the intervention and trial methods accordingly;
3. To determine the effectiveness of SIPC for people severely affected by LTNCs compared to standard care in the secondary outcomes: palliative care needs and other symptoms, patient psychological well-being and quality of life, caregiver burden/positivity and quality of life, improvement on patients and carers' satisfaction and communication;
4. To determine the effects of SIPC for people severely affected by LTNCs on hospital admissions, length of hospital stay, emergency attendance and other service use over the trial period;
5. To determine the cost-effectiveness of SIPC for people severely affected by LTNCs;
6. To understand how the change process may work and to identify components of the SIPC that are most valued by patients, their families/caregivers and other healthcare professionals;
7. To determine how the effects change over time, whether earlier referral to the palliative care affects the subsequent response to palliative care, and when assessment or re-referral might be beneficial.

Our study follows the MRC guidance for the development and evaluation of complex interventions. We have completed development, modelling, feasibility, piloting and preliminary evaluation in one centre of a new SIPC and neurology service to improve symptom management and care for people severely affected by multiple sclerosis. In this study we wish to test this new service in a wider range of settings and for a wider range of neurological conditions. We will conduct a randomised pragmatic trial of SIPC offered by a Multiprofessional Palliative Care Team (MPCT) compared to standard care.

13.2 Trial Design

This is a randomised Phase III, fast-track controlled trial. It is a multicentre evaluation of a complex intervention, following the MRC guidance for the development and evaluation of complex interventions [[35]] This study incorporates:

(i) a set up and feasibility phase to refine recruitment and methods;
(ii) mapping usual care for patients with LTNCs across the different centres (by prior work collecting information about the services and during the study recording services received at baseline and in the standard care group);

(iii) a randomised controlled trial of SIPC (the intervention) offered from a MPCT compared to best usual care;
(iv) a qualitative component, to explore the ways that the SIPC affects patients and caregivers, how the change process may work, how SIPC may be improved and to interpret quantitative results;
(v) a survey of health professionals; and
(vi) economic modelling to estimate the NHS and societal resources required for and longer term impacts of SIPC.

14. Trial Intervention

14.1 Intervention Details

The conceptual model of SIPC builds on that developed by Wagner and colleagues in the US to describe a structured framework for chronic care [[36]] and that of the NHS long-term conditions model. There is a focus on personalised care planning, case management and supporting existing case managers (e.g. community matrons, existing and specialist nurses) [[18]]. SIPC is offered when patients are severely affected by their illness and problems, are highly complex and are at high risk of hospital admissions with a high need for care. Patients do not have to be actively dying and the aim is to reach patients before this stage, in the view that earlier intervention to personalise their care and engage them in care planning will improve outcomes and enable existing services to then provide better care. It will be offered by existing MPCTs, linked with local neurology and rehabilitation services. The service follows a standard procedure, mirrored from the evaluated SIPC in MS [[5 37]]. All staff involved in the study will be provided with a standard manual and trained in advance of the study commencing. These aspects will be refined during the feasibility and set-up phase. The training comprises individual training with the different palliative care teams and bringing representatives from the teams together. This will be repeated regularly throughout the study as staff change. Time spent on training will be logged for the purposes of estimating the intervention costs.

The MPCT comprises individuals specifically trained in palliative care from (at least) backgrounds in medicine, nursing and psychosocial care or social work. The MPCT has regular multidisciplinary meetings to review patients in their care and is able to visit patients at home in the community. We will ensure that the MPCPs are integrated into local MS, PRDs, and MND care teams to ensure coordination of care. For the purpose of this study, the MPCT will operate a key worker process where a specialist team member will take initial responsibility for a patient referred, although the key worker may change during the course of the intervention. This involves key features of case management, training of health professionals to support self-care ('guided care') and planning levels of specialist input depending on the needs of individuals. The key worker liaises regularly and integrates care with neurologists, nurses, rehabilitation, primary and hospice services to discuss patients of concern. Based on their visits and assessments the key worker generates a problem list with the patient and outlines a proposed action plan, agreed with the patient and family (which is thus individually tailored). This might involve a change in symptom management (e.g. drug change), contact with other services, and/or psychosocial support or counselling during the visit. The key worker reviews and revises, at a multiprofessional team meeting, the problem list, their assessment and the action plan to optimise the management of the patient and caregiver and plan future visits and liaises with other professionals, e.g. to agree medication or service change.

In MPCTs the nurses and allied health professionals do not usually have prescribing powers, they advise regarding medications, although this may change over the time of the project. The palliative care teams all have doctors who can prescribe, but would do so in liaison with the GP and neurologist. If the nurses began to prescribe we would record this, but they would do so in liaison with other professionals.

The theory of the intervention

People severely affected by long-term conditions, including neurological conditions, have many similar problems and concerns as those affected by advanced cancer, including symptoms, psychological needs and family/caregiver concern [[38]]. Specialist MPCTs successfully improve these problems for cancer patients, and are now available widely across the globe [[37 39]]. The Cochrane Handbook outlines [[40]] that if there is empirical evidence that similar interventions have an impact, or identical interventions on other populations, these are quite likely to have effectiveness. Thus, as a starting point it is reasonable to hypothesise that input from an MPCT will help people with long-term conditions. It has been advocated in the NSF for Long-term Conditions [[41]]. To explore this hypothesis further in our modelling stage, we found that people severely affected by MS, PRDs and MND had many similar symptoms to those affected by advanced cancer [[4]], with additional problems of loss and care co-ordination [[1-3]]. These needs are within the remit of MPCTs which offer a holistic approach attending to symptoms, psychological needs, and better co-ordination of care [[42]].

People severely affected by LTNCs often have a longer trajectory of illness than those with advanced cancer – and so our modelling found that staff, patient and caregiver groups favoured the idea of MPCT input for a short term, working in a way that was well integrated with existing neurology and rehabilitation services, in particular disease specific nurses (such as MS or IPD nurses), who are often the mainstay for the many patients in the community. Figure 1 shows our model of how we anticipate the intervention to work. An intervention and training manual has been developed for use in this trial.

Conceptual Framework for the intervention

SIPC is modelled on our work to date following the MRC guidance which included: literature reviews [43] and qualitative studies [2 3] to determine need and to develop the theoretical underpinning of the service, appraisal of trial methods [4 5], service modelling and a successful phase II trial randomising 52 patients [25 28 37]. SIPC is a complex intervention [35] in that it:

- contains several components (assessment, symptom management, future care planning, follow up visits)
- aims to change behaviours by those staff delivering the intervention, those providing usual care to this patient group, and some changes on the part of patients and families
- targets patients, families and staff in primary, hospital and voluntary care, thus including different groups and organisational levels
- has several complex outcomes, including change in symptom management and hospital admissions
- is tailored to individual patient need and circumstances by those delivering SIPC and
- operates in a context where there may be some variability between patient groups and settings in the usual care provided to patients with long-term neurological conditions. Usual care is offered to patients in the intervention and control arms of the trial.

Control arm: Receives standard care services, details of which are recorded within the economic assessment for individual patient. Details of services available and usual practice are mapped for each centre during the set-up and feasibility phase. At the cross over point patients are offered referral to SIPC, as for the fast-track group, as if they had been on a waiting list. Patients and caregivers in both arms will receive usual care, including support from specialist nurses (most patients in the group will have these [28], although they are rarely able to see patients at home and are usually in contact every 3 months), neurology services (outpatient and in-patient), rehabilitation services, alongside community services, including general practitioners, district nursing services, and social services (for around 25%). A few patients will have physiotherapy and/or speech therapy as their needs determine. Patients with MND may be cared for in specialist MND centres, patients with PRDs and MS are usually under the care of neurologists with special interests in these conditions, as well as their primary care services. Some patients with more advanced disease will be in nursing or residential homes, and they will be offered the opportunity to participate in the study as well. Some patients may be identified on admission to hospital or through voluntary sector or social service.

Figure 1: Model of the Short-term Integrated Palliative Care intervention for people severely affected by Long-term Neurological Conditions

Need for the intervention [1-5]

Symptom burden
Loss of function
Pain
Nausea and vomiting
Feeling sleepy
Mouth problems
Constipation
Poor appetite
Spasticity
Fatigue
Depression
Bedsores
Incontinence
Communication difficulties
Cognitive problems

Lack of continuity & co-ordination in care

Lack of information about services

End of Life issues

Complex treatment choices

Informal caregiver burden

High health care costs

Repeated hospital admissions with concerns regarding care while in hospital

Short-term Integrated Palliative Care - to support personalised care planning, to empower patients with information and to support in management of symptoms and future decisions regarding care

Palliative Care
Multi-professional teams trained to deal with complex problems, including pain and symptoms
Holistic assessment
Patients and their families/close friends
Short-term - Usually 3 visits, over 6-8 weeks

1. Visit from key worker
Comprehensive palliative care assessment

- Symptom control and management
- Continuity and co-ordination of care, access to services
- Psychosocial needs. Information / communication needs

Need for advance care planning supported and improved
Assessment of caregiver
Agreed personalised problem list and action plan
Review problem list/plan with multi-professional team & liaison with relevant health, social and voluntary sector professions

2. Visit from key worker or other relevant team member
Review outcomes from actions already taken.
Reassessment. If immediate issues resolved, advance care planning. Revise personalised plan and actions. Plan for discharge.
Re-review personalised problem list/plan with multi-professional team & liaison with relevant health professions

3. Visit from key worker or other relevant team member
Review outcomes from actions already taken
Reassessment, revise plan. Reassessment repeated as required.

Discharge. Final Review with multi-professional team & liaison with relevant health professions

Primary outcome
Key symptoms assessed at 12 weeks
Pain
Nausea
Vomiting
Sleeping difficulty
Mouth problems

Secondary outcomes
Palliative care needs & other symptoms

Health-related quality of life & well-being

Psychological distress, anxiety & depression

Patient satisfaction, self-efficacy and other outcomes

Service use (hospital admission, length of stay & emergency attendance, other service)

Caregiver burden, positivity & quality of life

Costs

14.2 Frequency and duration of intervention

The length of intervention is usually 6-8 weeks from referral. This is broken downs as follows:

- 2 days to first telephone call (from receiving information about patient)
- Up to 5 days to first visit (i.e. end week 1),
- 2-3 weeks second visit (ie end week 3 – 4)
- 3-4 weeks third visit (i.e. end weeks 6 – 8)

Following referral, a key worker (usually a specialist palliative care nurse) from the MPCT contacts the patient within 2 working days (faster if needed) to arrange a visit within the next five working days to undertake a comprehensive palliative care assessment. The second face to face contact normally occurs within 2 weeks of the first face to face contact. There are normally 3 (2-4) follow-up visits to action, review and evaluate the proposed plan of care, and then discharge to local services as appropriate. There needs to be some flexibility to adjust to the individual needs and requirements of patient and their families. A small number (based on previous experience, 10%) will need continuing palliative care support and this will be provided on a needs basis. Otherwise, usually the teams will put in place the systems for improved care and have resolved immediate issues.

14.3 Intervention records

The palliative care team will keep a standardised proforma(s) for each patient (and family). This will record the main activities and services provided to patients, families and other professionals. This will include: for intervention visits who has contact, contact duration, clinical details and severity of main problems using aspects of the staff version of IPOS Neuro (using the standard methods as used in the NIHR C-Change project), any recommended changes to medications, what kind of activities were performed during contact, plan of care and referrals to other services. In addition at each visit, staff will record assessment of phase of illness (stable, unstable, etc) and functional status. A record of contacts outside the intervention visits will also be kept. To ensure fidelity of the SIPC we will undertake quality assurance and observation of activity in the centres throughout the trial.

14.4 Subject Compliance & Adherence

We will record and classify the level of adherence (compliance) in both intervention and control arms. This will be kept under review by the trial co-ordinator (as the research nurses will be blinded to the intervention). We estimate that we will classify compliance as follows (using a standard nomenclature), based on a combination of the research (patient interviews about particular services received, trial co-ordinator log, research nurse log) and clinical records (log kept by clinicians), and contact with clinical services:

- Complier (receives full intervention as planned)
- Partial complier / erratic user (receives some of intervention but not all, or recommendations not followed)
- Overuser (in frequent contact with the service)
- Contamination (when the control group access the service before due – cause will be recorded)
- Dropout –cause recorded using the MORECARE classification of reason for attrition [[44]]

Definitions of compliance and ways to assess these will be refined during the intervention development and mapping phase of the project. We will also monitor and review the recruitment rate and the adherence to the protocol and respond appropriately if necessary.

14.5 Concomitant Medication

This intervention is not a drug trial, but a service trial. Therefore, patients in both groups will continue their normal medications for the management of their underlying conditions. Their medications will be prescribed by the clinician responsible for their care (normally the GP and/or neurologist). In addition, the palliative care team may recommend medication to alleviate a symptom which is refractory despite optimal treatment of the underlying conditions. In this instance prescribing practice will follow regional and national best practice guidance (e.g. the Palliative Care Formulary (http://www.palliativedrugs.com/index.html) recommendations) and will be recommended in liaison with the patients GP and/or neurologist as appropriate depending on setting. In these instances in the team will record the change in their log of activity.

It is not possible to record all the normal medications patients for this range of conditions will be receiving, nor is it possible to receive all the possible recommended medications by the palliative care team as both lists are extensive. Patients have different neurological conditions, and will be under the care of expert neurologists for their treatment. A criteria for referral is that patients have symptoms which are refractory despite optimal treatment of the condition. Research has shown these patients, at this stage of illness, have on average 13 different symptoms which require expert assessment and treatment, and so the potential list of considered treatments is long and is available within the formulary listed above.

15. Research environment

The study will recruit patients from five areas of the UK (South London, Brighton and Hove/Sussex, Cardiff, Liverpool and Nottingham) all with MPCTs and neurology/rehabilitation services and sufficient catchment population. Additional sites may be added throughout the trial. We will seek adoption of the trial onto the NIHR UKCRN portfolio database, which will raise awareness of the study. The included sites and any potential additional sites are generally regional centres for neurological/rehabilitation services and have wide catchment areas. The services are consultant led and employ clinical nurse specialists for the relevant conditions. The majority of patient contacts are hospital based, with variable community outreach work. The sites' respective local areas have networks of palliative care services including in-patient hospices, community services and hospital support teams, coordinated by local and regionally based Palliative Care Networks. The sites encompass urban, suburban and rural areas with varying levels of deprivation and ethnic diversity.

16.Selection and Withdrawal of Subjects

16.1 Inclusion Criteria

<u>Patients</u>

1) Adults (aged 18 years or over) severely affected by advanced or progressive stages of the long-term neurological conditions (LTNCs) of either*:
   - Multiple Sclerosis (MS) - patients with either aggressive relapsing disease with rapid development of fixed disability or those with advanced primary or secondary progressive disease, often with limitation in a number of areas including gait and upper limb function. We do not define referral based on disability but would expect most patients to have an Expanded Disability Status Scale (EDSS) [[5]] of at least 7.5.
   - Parkinsonism & related disorders (PRDs) i.e.
     - Idiopathic Parkinson's Disease (IPD) Hoehn and Yahr (H&Y) [[45]] stages 4-5 OR
     - Progressive Supranuclear Palsy (PSP) Hoehn and Yahr (H&Y) [[45]] stages 3-5 OR
     - Multiple System Atrophy (MSA) Hoehn and Yahr (H&Y) [[45]] stages 3-5
   - Motor Neurone Disease (MND) all stages

AND

2) who are deemed (by referring/usual care clinicians) to have:
   - an unresolved symptom (e.g. pain or another symptom) which has not responded to usual care
   - AND at least one of the following: unresolved other symptom (e.g. breathlessness, nausea / vomiting, spasticity, fatigue); cognitive problems; complex psychological (depression, anxiety, loss, family concerns), communication/information problems and/or complex social needs.

AND

3) who are able to give informed consent^ OR where their capacity can be enhanced^ (e.g. with information) so they can give informed consent OR where a personal consultee^ can be identified and approached to give a opinion on whether or not the patient would have wished to participate in the study.

AND

4) are living in the catchment area of the Short-term Integrated Palliative Care (SIPC) Service

* Diagnosis must have been established by a specialist neurological assessment.
^ When a person lacks capacity to consent for themselves the procedures detailed in the Mental Capacity Act (2005) are adhered to.

We expect patients to be in the advanced or progressive stages of disease. They may be living at home (most common), in a nursing home or in hospital at the time of recruitment. We will develop a proforma for referring clinicians to complete (covering contact and clinical information and important reasons for referral/selection).

Caregivers

1) Adults (aged 18 years or over) identified by the patient as the person closest to them, usually a family member, close friend, informal caregiver or neighbour
   AND
2) able to give informed consent and to complete the questionnaires

Professionals/commissioners

1) professionals/commissioners who are involved in the care of patients with LTNCs

   OR

2) professionals (of neurology or palliative care services) who are part of a team which is involved in the delivery of the OPTCARE Neuro intervention

16.2 Exclusion Criteria

Patients

Patients who meet the inclusion criteria but:

1) are already receiving specialist palliative care currently or have done so in the last 6 months (please note: seeing any palliative care specialist counts as exclusion criteria)
2) lack capacity and have no family member, friend or informal caregiver who is willing and available to complete questionnaires about their own and the patient's symptoms and circumstances

We will log all referrals to the trial and map what data we ethically are able to on those who are not included, including reasons for exclusion, where patients who live outside the catchment area are based, those are already receiving palliative care, refusals and those excluded because they lack capacity and have no identifiable personal consultee or informal caregiver.

We will test these inclusion and exclusion criteria in the initial feasibility stage and piloting and will propose modifications if required.

16.3 Selection of Participants

16.3.1 Raise awareness

These build on our successful experiences in our phase II study [5 37]. We raise awareness by:

- Some weeks prior to the trial opening we will commence a programme of raising awareness and workshop sessions in the study sites, (e.g. lunchtime seminars) which continue throughout recruitment. This programme will include information about the trial, why it is being conducted, equipoise, how to identify and refer patients, general information on palliative care needs, focussing especially on those most severely affected by long-term neurological conditions. These steps to raise awareness will be completed by neurology, voluntary and community services, service user groups, primary care, hospital services and palliative care.
- Developing posters and flyers detailing the trial, the research personnel and lead clinicians, to be displayed in appropriate places.
- Developing a website about the trial which will be linked to the relevant patient led society web sites and DenDRon Websites.
- We will also discuss with staff in centres the inclusion and exclusion criteria for our trial. Feedback on trial recruitment will also be presented regularly to enhance motivation [46].
- Working with the PPI groups.

To ensure appropriate identification and recruitment we will train researchers and local clinicians in this. In addition we will display information about the study on posters in neurology clinics and local relevant services. The recruitment will be through the neurology teams, particularly outpatient clinics, but also from the inpatient setting. Research nurses will liaise directly with these teams. This will ensure the accuracy of

disease diagnosis. We do not plan to recruit through primary care owing to the large number of GP practices required to achieve a high yield, although we will include primary care in our awareness raising and will accept referrals if they come from this route.

1. Patient recruitment (when patients have capacity)

The identifying clinician will give written information to the patient about the study. If the patient is interested and agrees for their details to be sent to the researchers to learn more about the study, the clinician will complete a standard referral screening form to check that patients meet the inclusion criteria and send this to the local research team. The research team will contact the patient, explain the study, send out written information and arrange to visit (after a minimum of 24 hours unless the potential participant wishes to waive this period, in which case the researcher will record the participant's stated reason). The visit can be at their home or anywhere else the participant feels would suit them. At the visit the researcher answers any questions, takes consent and administers the baseline questionnaire with the patient. As part of the consenting process the researcher will discuss the need for the patient to nominate a consultee in case their capacity fluctuates during the course of the study (see below for Mental Capacity Act information).

2. Patient recruitment (when patients lack capacity, or capacity has become insufficient since the initial identification, also after attempts to improve capacity have not succeeded)

If a patient meets the inclusion criteria but the clinical team or visiting research nurse assess them (using clinical judgement, in line with local/policy guidance at site) to have reduced capacity (see below Mental Capacity Act information) they would discuss inclusion with the informal caregivers, family members or close friends, and the patient, if appropriate, to determine who is the most appropriate person to act as the personal consultee. They would then send relevant information to make contact with the personal consultee. The research team will contact the personal consultee, explain the study, send out written information and arrange to visit (after a minimum of 24 hours unless the personal consultee wishes to waive this period). The visit can be at the patient's home, nursing home, hospital or elsewhere (within reasonable travelling distance) that the personal consultee feels would best suit them. The researcher would reassess the patient and if capacity continues to be insufficient the researcher would obtain the opinion of the consultee as to whether the patient would have liked to be included in the study.

3. Caregiver recruitment

We also wish to gather the views of the informal caregiver. The identifying clinician or the researcher will give written information to the person that the patient identifies as nearest to him or her (such as a family member or informal carer if present) about the study. If the informal carer is interested and meets the inclusion criteria they can be approached and consented (following the minimum of 24 hours unless they decide to waive this period).They may consent at the same time as the patient consents, or separately. The caregiver will complete the questionnaire themselves and return to the researcher at the visit or post it back in a prepaid envelope. Other alternatives, e.g. face-to-face assistance can be discussed. There will be a slightly extended questionnaire for the carergiver to complete if the patient lacks capacity from the start of the study so that we are able to capture some demographic details about the patient too.

16.3.2 The Mental Capacity Act 2005

We apply the principles of the Mental Capacity Act (MCA) 2005. We include patients who might lack capacity to consent themselves, as cognitive impairment is not uncommon in individuals at advanced stages in the disease trajectory. The feasibility components of our phase II trial indicated that exclusion of those with cognitive impairment excluded those with the highest disease burden, who are likely to benefit most from the intervention. Exclusion hampers the generalisibility of the study for the population it seeks to benefit. We apply the principles of the Act, in other words the work is justifiable because the patients stand a chance of benefiting from inclusion, the research is likely to benefit people like those included in the study, and we cannot obtain information about the effects of SIPC on patients who are most severely affected and lack capacity, without including such patients in the study. Assent is sought from personal consultees (usually close caregivers/family members) who know the patient sufficiently well to give an opinion as to whether or not the individual would have wanted to participate. This approach was tested in the phase II trial, proved successful and was strongly supported by the MS Society and other patient groups. Data are collected from both patients and caregivers, so where patients have low levels of cognitive impairment, we are able to analyse the secondary outcomes, and caregivers assessment of the patient outcomes (separately from patients' self assessments). Incapacitous patients who show distress (verbally or non-verbally) apparently as a result of participation, or voice any objection to the study can be withdrawn at any stage and that withdrawal will not affect their usual care.

16.3.3 Process of consent and assent for adults lacking capacity

The process of consent and assent for adults lacking capacity is shown in Figure 2. The commonality of cognitive impairment in advanced and end of life care requires the inclusion of people with impaired mental capacity in the study. The MCA informs the process of consent protocol and recent studies involving adults lacking capacity [47-49]. All participants are considered to have capacity unless established otherwise and all practicable steps are taken to enable individuals to decide for themselves if they wish to participate. A potential participant's level of capacity is discussed with the referring clinician to identify participants with possible impaired capacity and to anticipate the likely consent procedure. Capacity is established when meeting the individual using the MCA four step process: 1) the individual is able to understand the information about the study; 2) retain the information (even for a short time); 3) use or weigh up that information and 4) communicate their decision [50]. Potential participants' mental capacity is anticipated as ranging from able to give informed consent to lacking capacity to give informed consent. We have developed processes of consent and assent that are tailored to an individual's level of capacity, incorporating varying levels of capacity and anticipating that some participants may lose capacity during the study because. Incorporating different processes of consent and assent is used in research studies on end of life care [47-49]. This intends to enable individuals with varying levels of capacity to decide for themselves if they wish to participate, and to incorporate a process of assent for adults lacking capacity.

16.3.4 Consent in the moment for participants with impaired capacity

For adults with impaired capacity, who are able to understand, retain and weigh-up information in the moment, a process of consent in the moment is used with ongoing consent whereby informed consent to participate is reaffirmed prior to each data collection point [6]. The approach of consent in the moment was developed and used in studies involving adults with dementia and/or cognitive impairment [6 51]. If a participant's capacity declines so that they are no longer able to give informed consent in the moment, the researchers follow the procedure for adults lacking capacity detailed below.

16.3.5 Advance consent and assent for participants who lose capacity

An advance consent is incorporated in anticipation that some participants may lose capacity and may no longer have capacity to indicate their right to withdraw from the study. The process of advance consent is informed by previous studies with older people [47] and on end of life care [52]. Participants able to give informed consent are asked to indicate should they lose capacity in the future if they would wish to continue to be involved in the study, and if yes, then they are asked to nominate a personal consultee (e.g. next of kin). The personal consultee is approached if the participant loses capacity to such an extent that they are no longer able to indicate their right to withdraw from the study, or to complete patient reported outcome measures, requiring instead a proxy informant (e.g. informal or formal carer). The procedure of assent for adults lacking capacity is followed to ascertain the personal consultee's opinion on the individual's continued participation (see below). There will be instances in which a patient's capacity has to be enhanced, e.g. by providing only essential information about the project, breaking down complicated information into smaller points [53]. We already aim to make the information sheet easily accessible and to give patients time to reach their decision and pose questions. If patients' capacity can be enhanced they will be able to consent. If not, a personal consultee will be sought.

16.3.6 Assent for adults lacking capacity

When an adult lacks capacity a personal consultee is sought to give an opinion as to whether in his/her knowledge of the potential participant they would have wanted to participate in the study had they had capacity to indicate this, and that participation would not cause undue distress [47 50]. A personal consultee comprises next of kin, immediate carer or attorney with Lasting Power of Attorney. Identified consultees are given an information leaflet about the study, a letter detailing why they have been chosen as a consultee and their responsibilities as a consultee. The consultee documents are informed by research with elderly people [47], the MCA [50] and MCA guidance [54].

16.3.7 Documentation of study participation

All participants who give written consent to participate will be given a copy of the information sheet to retain and keep, and all consultees giving written assent. Participants are offered a copy of their signed consent form to keep if they wish, and consultees a copy of their signed assent form. A copy of the signed consent/assent form will be filed in the participant's medical notes. The research team will retain the original

signed consent form. For those people who give consent /assent for their general practitioner (GP) to be informed about their participation in the study (on the consent form), the GP will be sent a letter.

Figure 2: Process of consent for mental capacity

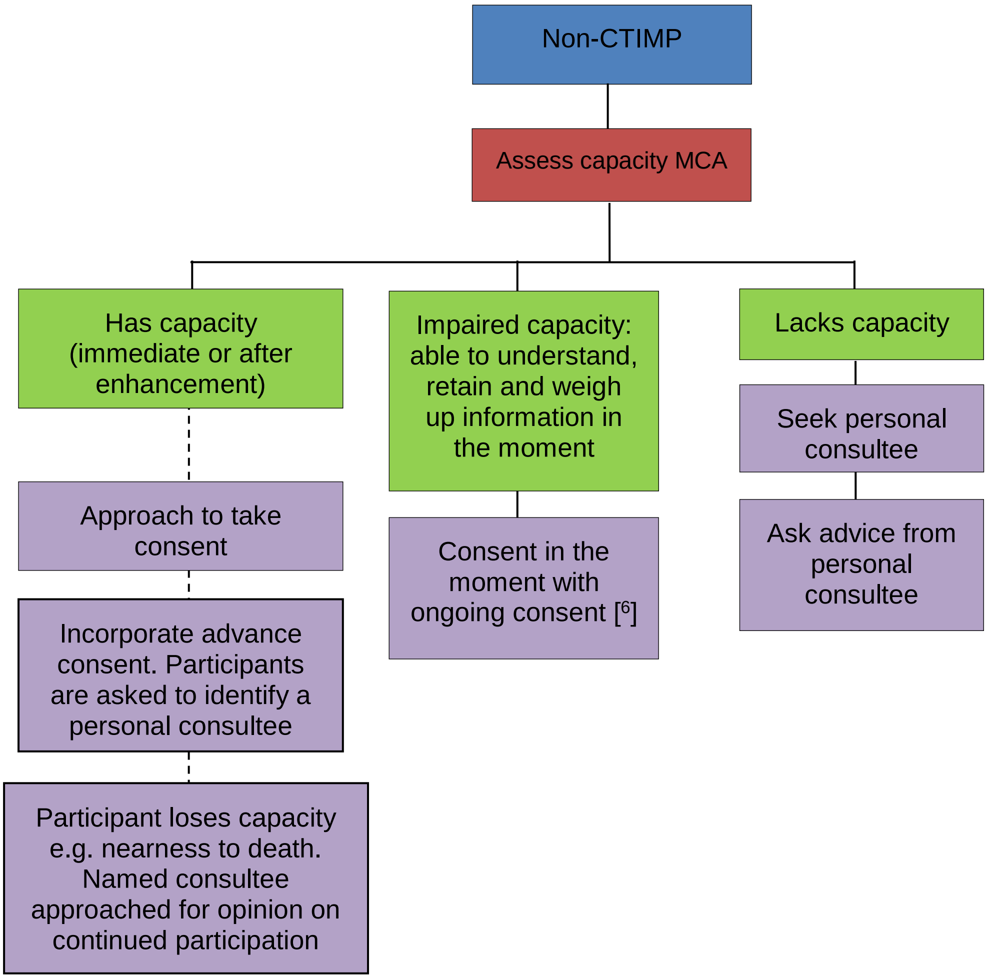

16.4 Randomisation Procedure / Code Break

Following consent, baseline interview and registration of patient on the MACRO eCRF, the patient will be allocated a unique PIN which will identify them throughout the course of the trial. The individuals will be randomised to one of the trial arms using minimisation method, stratified by center, primary diagnosis (MS, IPD, PSP & MSA, and MND) and cognitive impairment. Randomisation will be done in a 1:1 ratio and will be at the patient level. Randomisation will be performed using an online randomisation system based at King's Clinical Trials Unit (King's CTU), which can be accessed at www.ctu.co.uk by clicking 'randomisation – advanced' on the lower right hand side of the page. The trial coordinator (or someone supervised by the TM) will be notified of the trial arm allocation of the patients and arrange intervention and/or follow-up visits. The research nurses who will conduct the interviews and the trial statistician will be blinded to the trial arm allocation. Overall, we do not expect to need to unblind (code break) to the research nurses or to the trial statistician during the periods of trial and primary analysis. After the primary endpoint of 12 weeks, in a small number of cases research nurses are needed to conduct a qualitative interview with a patient who has received the intervention, where we cannot find another researcher to do this interview, which will effectively unblind the research nurses. We will keep this to a minimum and ensure it is after the primary endpoint of the study.

Any problems with the online randomisation system should be reported to the Trial Manager or the King's CTU at ctu@kcl.ac.uk or on 0207 848 0532.

16.5 Withdrawal of Subjects

Patients can withdraw from the the study at any time for any reasons without affecting their usual care. Should a patient decide to withdraw from the study, all efforts ethically appropriate will be made to report the reason for withdrawal as thoroughly as possible. Should a patient withdraw from receiving intervention only, efforts will be made to continue to obtain follow-up data, with the permission of the patient or family as appropriate.

16.6 Expected Duration of Trial

The trial will run for 40 months, which includes trial set up, recruitment and analysis of data. Patients will receive their intervention for approximately 6-8 weeks and will be followed up for 24-26 weeks (from start of trial, not necessary start of intervention). End of trial is defined as the last patient’s last study visit.

17. Trial Procedures and Data Collection

17.1 Main data collection by visit

Following referral, the key worker (usually a specialist palliative care nurse) from the MPCT contacts the patient within 2 working days to arrange a visit within the next five working days to undertake a comprehensive palliative care assessment. In keeping with the multi-disciplinary ethos of palliative care, the detailed assessment is discussed with the MPCT who suggest ways to improve management of physical, emotional, social and other problems, provide specialist welfare benefits advice, liaises with and acts as a catalyst with local health services, both primary and specialist teams, and social care (See Figures 1 and 3). There are two follow-up visits to action, review and evaluate the proposed plan of care, and then discharge to local services as appropriate. The key worker liaises regularly and integrates care with neurologists, nurses, and rehabilitation, primary and hospice services to discuss patients of concern. The key worker records the nature of assessments and services provided.

17.2 Research interviews

Interviewers undertake face-to-face interviews with patients (at the patient's location of choice (e.g. home or at a clinic)) and assist as required in self completion of caregiver questionnaires. These are conducted according to a standardised schedule, using trained experienced interviewers who follow a manual for researchers, who are blind to treatment allocation and accommodated separately from the intervention team. Assessments from the nearest caregiver, family member or friend are planned to coincide with the patient assessments (ie at baseline, 6,12,18,24 weeks post randomisation). Usually they will self-complete the follow up questionnaires and return these by post to the project team at King’s College London. If this is not possible telephone assistance or face-to-face interview will be offered. In some instances caregivers may need a visit, or will choose to self-complete the questionnaire during the patient interview. We have used this method successfully in studies with people with MS and PRDs. For adults lacking capacity baseline and outcome measures are obtained from the informal caregiver interviewer as above. The use of a proxy informant is common in research on palliative care associated with patients’ advancing illness and deteriorating condition, and the importance of capturing data at points of deterioration when a patient may most benefit from palliative care, notably the last days of life [55]. Research on proxy informants indicates higher agreement between patient and caregiver dyads than in patient and health care provider dyads [56]. In addition the informal caregiver provides the baseline information about the patients demographic circumstances and clinical history (e.g. age, educational level, diagnosis, time since diagnosis) as would normally be collected in the patient interview.

17.3 Distress Protocol

There may be some distress and burden in completing the questionnaires and taking part in the study. However, it is also found that many patients and families value taking part in research and want to have the opportunity to give their view. We have found that including the opportunity for open comments, not only gives rich supplementary research data, but is highly valued by patients and families as they feel ‘heard’ by those researchers [57-60]. Therefore we have incorporated this approach into our study. All research nurses and those conducting interviews will be centrally trained and supported in the work and we will ensure that all have appropriate prior experience (such as in palliative or neurological care). All of the research team will have completed Good Clinical Practice training, and specific training on addressing distress in palliative care. Research nurses will follow a distress protocol if patients or families become upset during the interviews or are found to be upset or in distress on arrival. Should this be the case they will first offer to pause, postpone

or stop the interview and advise again that participation is voluntary. In the case of severe distress the patient will be encouraged to share his or her feelings with a member of their healthcare team or the nurse offers to contact the neurology team. We anticipate distress caused by the research will be very infrequent, given the general nature of the questions within the measures used, and is likely to reflect advanced disease and not the questionnaires themselves. If participants disclose any ideation of self-harm or other risk to themselves or others, this will be dealt with as an urgent matter and clinical help will be sought. This will be in agreement with the participant however, if the research team believes the participant is at imminent risk and refuses to allow voluntary disclosure, the research team will breach confidentiality. Based on prior experience, we anticipate this will be a very rare occurence. Provision will be made to ensure the researchers have PI or senior back up available by phone whenever they are undertaking data collection. We make it clear that patients and families can omit any questions they don't want to answer and can withdraw at any time from the service and the study with no effect on their usual services. We have ensured that we do not include terms that might be distressing, such as "end of life" or "prognosis", on the information leaflets or questionnaires. The palliative care service likewise takes a listening and individual approach, so that patients who may not want to talk about issues in relation to care, but want symptom support, can receive those elements of the service they want.

17.4 Qualitative component

Integrated within the trial is a qualitative component, to explore: (A) how the intervention is delivered in practice and (B) how the change process of SIPC may be working and which aspects of SIPC are most valued or impact most on patients and caregivers experiences of care and enhance the service model of SIPC. The methods build on those tested in our phase II trial [5 28 37 61]. We select a maximum variation sample of patients and caregivers who received SIPC (at week 12 for the intervention group and week 24 for the standard group) and health professionals in the study sites (e.g. community matrons, specialist nurses, primary care, neurologists). Case selection is based on socio-demographic variables (e.g. age, gender, educational level), neurological condition, severity of symptom presentation and change over time (baseline to 6 weeks or 12 to 18 weeks) in the primary outcome measure (IPOS Neuro-S8) to encompass patients with e.g. reduction symptom burden, no change, and increase. The qualitative interview schedule is informed by our phase II development work and previous literature [62 63] and explores the experiences of receiving SIPC, deductively (e.g. relevance of outcome measure change on daily life), and inductively (e.g. patient reported experiences of SIPC)[64]. All research nurses will be trained to conduct these interviews (after the 12 week data collection point for the immediate intervention and 24 week data collection point in the control group). If possible, we try to find a different interviewer to minimalise the unblinding of research nurses. Research nurses will be trained in conducting qualitative interviewing. The interviews are digitally recorded and transcribed and anonymised prior to analysis [65]. We will pilot the qualitative interview during the set-up phase.

Non-patient focus groups are conductedwith providers and commissioners from different sites to explore the perceptions of SIPC, the local contexts and processes of SIPC delivery and implementation. They are identified by research teams from different sites, who will provide the research team at King's College London with a list. The research team will approach professionals via email. The focus groups are incorporated in to project workshops with providers and commissioners, for example, training days, project updates. This minimises participants' time away from practice, and maximises opportunities to understand the context and description of practice and integration with SIPC. Each group comprises representatives from the respective sites and disciplines involved in the care provision. Where attendance at a focus group is not possible, individual interviews are conducted (either face-to-face or by telephone) in order to accommodate the busy schedules of healthcare professionals and allow representation from all sites. The groups are facilitated by a single researcher experienced in qualitative research methods and an observer (applicants Evans and Jackson are both experienced in qualitative methods) to document, for example, group processes and interactions. The groups are digitally recorded, transcribed and anonymised prior to analysis [65].

17.5 Survey of health professionals

The survey of health professionals will be a brief questionnaire distributed both at the beginning and at the end of the intervention, focussed on their expecations/views of SIPC, how it has or has not affected the process of care, how it might be improved and ways to best meet the needs of patients and families with LTNCs. It will be offered via e-mail, with data collected using a web based tool (e.g. Keypoint), and space for open comments. Research teams at the different sites will identify the neurology and palliative care team members involved in the intervention and provide an email list to the research team at King's College

London. The researcher will be sending out the survey, with up to 2 reminders. If surveys are completed, informed consent is assumed. This will be detailed on the information sheet.

Figure 3: Summary schedule of study

Timeline	Fast track (immediate referral to palliative care service)	Standard care (waiting list control)
Consent and Baseline interview	a	a
Randomisation		
2 working days from receiving information about patient	b	
1-6 weeks	c	
6 weeks	d	d
6-8 weeks	e	
12 weeks	f	f
2 working days after receiving research nurse data from week 12 interview		g
12-18 weeks	h	i
18 weeks	j	j
18-20 weeks		k
24 weeks	l	l
24-26 weeks		h

a	Baseline research interview and consent on entry to the study before randomisation
b	Palliative care assessment within 2 working days
c	Palliative care team care, including assessment, treatment, referral
d	Research interview at 6 weeks after randomisation
e	Palliative care team care continues usually ending by 6-8 weeks, with referral on for those patients needing long-term care

f	Research interview at 12 weeks after randomisation
g	Standard care group now offered palliative care team who see person within 2 working days of receiving information
h	Patients now discharged from palliative care team, if have been referred on some under care of community team, which continues from this point
i	Palliative care team now providing care in standard care group, following their 12 week wait
j	Research interview at 18 weeks
k	Palliative care team care continues usually ending by 6-8 weeks, with referral on for those patients needing long-term care
l	Final research interview at 24 weeks after randomisation

18. Assessment of Efficacy

18.1 Primary Efficacy Parameters

Primary efficacy parameter is the combined score of eight key symptoms as measured by the IPOS Neuro-S8 at 12 weeks. This is (based on) a validated scale used among people with LTNCs that is shown to be responsive to change. We have chosen this primary outcome based on the results of the phase II trial and our modelling work: patients consider these are all important symptoms in neurological conditions; the SIPC aims to improve several complex symptoms which interact; these symptoms are often overlooked by existing services but impact on quality of life; this measure was more responsive to change than the quality of life measure. Psychometric properties of the five items of the IPOS Neuro-S (IPOS Neuro-S5) tested in the control group of MS patients in our phase II trial found that it correlated well at baseline with the much longer MSIS-29 (Spearman rho=0.74), although the correlation reduced over time, as scores on the MSIS deteriorated or were missed. Internal consistency, indicated by Cronbach's alpha, for baseline assessments was 0.82, a good value for a 5-item scale. Test-retest reliability of scores over two assessments (where patients did not receive the intervention and were assumed to be more stable) showed an Interclass Correlation Co-efficient (ICC) of 0.81. Similar properties are found when the IPOS Neuro-S5 is tested in patients with PD. In a sample of 82 patients, ICC over two assessments was 0.72. We have used the IPOS Neuro-S5 among people severely affected by many conditions (cancer, renal, MS, PD, PSP, MSA, and in both randomised controlled trial and longitudinal observational study design [28 29 61 66]. The IPOS Neuro-S5 exhibited promising psychometric properties in our study samples, though it has not yet been validated in larger populations or in people affected by MND. However, this brief measure is so far the best we can use, given the floor effects of many measures among people severely affected by a long-term neurological conditions [66].

18.2 Secondary Efficacy Parameters

1) Patients' palliative needs and symptoms: Integrated Palliative care Outcome Scale for neurological conditions (IPOS Neuro) (based on a commonly used and well validated measure in palliative care research and clinical practice) [4 67-70];
2) Patients' health-related quality of life and well-being: EQ-5D and ICEpop CAPability measure for Adults (ICECAP-A) [71]. EQ-5D is a 5 item measure (mobility, self-care, usual activities, pain/discomfort, anxiety/depression) used to generate quality adjusted life years (QALYs). It has been used in numerous economic evaluations including those for neurological conditions. It will be the main measure in the cost-effectiveness analysis. The ICECAP-A is a new capability/well-being measure for use in economic evaluations. It has 5 items (feeling settled and secure; love, friendship and support; being independent; achievement and progress; enjoyment and pleasure) each scored 1-4 [72]. The possible states derived from the ICECAP-A are currently being valued in a way appropriate for QALY calculations (this will be completed before our analyses). QALYs generated will be used in secondary cost-effectiveness analyses; or the Integrated Palliative Outcome Scale for neurological conditions (IPOS Neuro);

3) Patients' psychological distress: Hospital Anxiety and Depression Scale (HADS) (patient reported);
4) Patients' satisfaction, self-efficacy and other aspects: patient satisfaction scale (modified FAMCARE-P16) [73], Self-Efficacy to Manage Chronic Disease Scale (SEMCD), an advance care planning scale (as used by Tuck et al 2013 [74]) and some questions covering patients' experiences of the study;
5) Hospital, service use and survival: hospital admissions, length of hospital stay and emergency attendances during the study and other health service use extracted from clinical records and also recorded using an adapted version of the Client Service Receipt Inventory (CSRI) at 0, 12, and 24 weeks [75]. The CSRI has been used in around 300 studies internationally. Services will include specialist palliative care, other primary and secondary healthcare, medication, social care, and care from family members. Where possible we will supplement CSRI data with data from hospital-based information systems. Employment status and welfare benefits will also be recorded. Survival (days from consent to death) for those patients who die irrespective of cause, will also be evaluated;
6) Caregiver assessment of patients' problems and service use: IPOS Neuro and CSRI (due to cognitive impairment for some patients, to be analysed separately from patient data);
7) Caregiver burden, positivity, quality of life and satisfaction: 12-item Zarit Burden Inventory (ZBI-12) and positivity [76], quality of life (VR12) [77] and the carer satisfaction scale (modified FAMCARE 2) (self-assessed by caregivers) [73 78];
8) Observer (completed by the researcher) assessment of the patients problems: Support Team Assessment Schedule (STAS), an observer rated assessment of palliative care problems – which has several items overlapping with the IPOS Neuro, and is designed for use through independent assessment [67].

Table 1: Candidate questionnaires that may be used in the study

Reporter	Domains assessed	Questionnaire
Patient	Symptoms & Palliative care Outcome	1. Integrated Palliative care Outcome Scale for neurological conditions (IPOS Neuro S8)
	Quality of life & Well-being	2. EuroQoL (EQ-5D)
		3. ICEpop Capability measure for Adult (ICECAP-A)
	Psychological distress	4. Hospital Anxiety and Depression Scale (HADS)
	Hospital & Healthcare service use & costs	5. Client Service Recipient Inventory (CSRI)
	Satisfaction & other aspects	6. Modified 16-item measure of patient satisfaction (FAMCARE-P16)
		7. Self-Efficacy to Manage Chronic Disease Scale (SEMCD)
		8. Advance Care Planning
Carer	Proxy patient symptoms & palliative care outcome	9. IPOS Neuro S8 – Carer
	Caregiver burden & positivity	10. 12-item Zarit Burden Inventory (ZBI-12) + positivity – Carer
		11. Veterans Rand 12 item Health survey (VR-12) – Carer
	Hospital & Healthcare service use & costs	12. CSRI – Carer
	Satisfaction & other aspects	13. Modified 17-item measure of carer satisfaction (FAMCARE2)
Professional	Cognitive impairment	14. 6 item Cognitive Impairment Test (6CIT)
	Functional/Neurological disability	15. Barthel Index (BI)
		16. Modified Fried's Frailty Criteria
		17. Northwick Park Dependency Scale (NPDS)
		18. Expanded Disability Status Scale (EDSS) – for MS
		19. Hoehn & Yahr Scale (H & Y) – for PRDs
		20. Bulbar or limb – for MND
		21. Australian modified Karnofsky Performance Status
	Patient symptoms & PC outcome	22. Support Team Assessment Schedule (STAS)
	Adverse Events	23. Adverse Events

*MS: Multiple Sclerosis; PRDs: Parkinsonism & Related Disorders; MND: Motor Neurone Disease

In choosing these outcome measures, we have carefully selected scales to avoid significant floor and celing effects in a very disabled population. We have chosen measures that are simple and easy top use, and are not too lengthly, as the population cannot complete very long questionnaires. An observer rated measure is included to collect data among patients with cognitive impairment.

19. Assessment of Safety

19.1 Specification, Timing and Recording of Safety Parameters.

Number of deaths, emergency attendances, hospital admissions and length of hospital stay in each arm in total and by disease will be monitored. Any noticeable increase in these parameters will be subject to full investigation which will be acted upon.

19.2 Procedures for Recording and Reporting Adverse Events

We did not observe any harmful effect in our Phase II trial of SIPC among people severely affected by multiple sclerosis (MS). We are not expecting to see any harmful effect in this trial. However, we will monitor deaths, survival, hospitalisation, length of hospital stay and emergency attendance by trial arm and by diagnosis, and six-monthly report these statistics to the Data Monitoring Committee (DMEC) and Study Steering Committee (SSC).

19.3 Stopping Rules

Any one of the following:

- If the study cannot achieve a minimum of 25 recruited at 6 months from the start of recruitment to the study;
- If SIPC cannot be delivered to at least 80% of patients in the SIPC arm within 3 weeks of randomisation; more than 10% of patients in the control arm received specialist palliative care within 11 weeks of randomisation;
- If the DMEC and/or SSC raise any new safety or ethics concerns over the trial process and make recommendations to discontinue the trial;

These rules will be reviewed and approved by SSC and DMEC once these committees are formed. If the study is prematurely discontinued, active participants will be informed and no further participant data will be collected.

20. Statistics

20.1 Sample Size

Based on the data from our Phase II MS trial, the five centres altogether need to recruit 356 patients. In view of the advanced illness in this patient group we have allowed 20% attrition (phase II trial attrition from death or illness: 3/52) to the primary 12-week outcome, giving 296 patients, or 148 in each arm with both baseline and 12-week outcome data. The correlation between baseline and the 12-week outcome in the pilot study was 0.55. Using generalised linear model to adjust for the baseline score, with two-sided alpha=0.05 and correlation of 0.40, the study will have 80% power for a medium effect size of 0.30. To allow for heterogeneity across conditions and centres, we used conservative figures (eg. correlation 0.4 rather than 0.55; 20% attrition) to estimate the sample size. For the qualitative study, we estimate a sample size in each study site of seven patients/caregivers, total 35 patients/ caregivers in qualitative sample and six service providers/service commissioners from each site, total of 30 providers/commissioners. For the pilot phase we aim to recruit 15-25 patients/caregivers. The survey of health professionals will be primarily descriptive. It will be sent to a random sample of 200 health professionals, 40 in each area, with a mix of primary and secondary care staff identified as those referring to or interacting with SIPC. We estimate a response rate of 50-60% with 2 reminders giving at least 100 completed questionnaires.

20.2 Analysis

20.2.1 Quantitative analysis

Formal review of recruitment rates will take place at month 15. Criteria for continuation are: recruitment of at least (minimum) 25 patients, SIPC commenced within 3 weeks of randomisation in at least 80% of patients in the SIPC arm, Specialist palliative care received within 11 weeks of randomisation in less than 10% of patients in the control arm. Recruitment rates will continue to be monitored throughout the trial. The analysis will focus on assessing the criteria for continuation and any modifications needed. Analysis will be undertaken according to CONSORT guidelines by our Clinical Trials Unit with the trial statistician being blind to treatment assignment. A flow chart will present the follow-up rate for each group, with the reason for non-completion of the primary outcome. In view of the short duration of the trial no interim analysis is planned.

Attrition will be classified according to the MORECARE classification as to whether it is Attrition Due to Death (ADD), Attrition Due to Illness (ADI) or Attrition at Random (AaR) [79]. We will explore and report reasons for missing data, and any factors associated witih missing data, in particular differences between study arms. As data are more likely to be missing when patients are ill, we expect missing data to be missing not at random.

Analysis of primary outcome (IPOS Neuro-S8): The primary analysis will be on an intention-to-treat (ITT) basis using generalised linear mixed model with centre as a fixed effect, adjusting for baseline score of IPOS Neuro-S8 and for variables from the baseline assessment for which treatment-group imbalance is found. The intervention effects and 95% confidence intervals will be estimated. We will test for intervention by center interaction. The missing data will be explored and dealt with using appropriate techniques. The robustness of the findings will be assessed and further uncertainties will be addressed by further sensitivity analysis. Except where described below secondary outcomes (e.g. IPOS Neuro, EQ-5D, HADS, ZBI-12) will be analysed in similar fashion to primary outcome. The Wilcoxon (Breslow) test, which is more sensitive to early survival differences as is appropriate in a fast-track or wait list trial design, will be used to test for differences in survival between study arms. Where regression diagnostic checks suggest model distributional assumptions are not met, confidence-intervals and test statistics will be calculated using boot-strap methods.

Tests of homogeneity of treatment effect across the items (symptom profile) of the IPOS Neuro-S8 will be undertaken within the differential-item functioning framework of Item-Response Theory. Bar charts of mean/median data (as deemed appropriate) and box plots showing means and 95% CI for each treatment group at each time point will also be produced. For describing patient and caregiver reported outcomes we will use descriptive analysis to summarise the data by trial arm. We will monitor adverse events. Numbers of deaths in each arm in total and by disease will be reported together with the relative risk and confidence intervals estimated using Cox regression, stratified by disease. An analysis joint with QoL ratings will also be undertaken. We will use latent growth curve model to evaluate how the intervention effects on primary and secondary outcomes change over time.

Cost-effectiveness will be assessed by linking data on service cost differences and two outcome measurements (IPOS Neuro-S8 and ICECAP-A) differences. If post SIPC has significantly lower costs and significantly better outcomes then SIPC will be deemed to be more cost-effective. If costs are significantly higher and outcomes significantly better or if there is uncertainty in these findings then we will use the net benefit approach and cost-effectiveness acceptability curves to assess cost-effectiveness. Cost-utility analysis will be conducted in the same way as the cost-effectiveness analysis but will use QALYs (derived from the EQ-5D-5L) as the outcome measure.

20.2.2 Qualitative analysis, survey and integration of all data

Data analysis draws on Coffey and Atkinson's [80] iterative approach of coding and describing the data, generating categories, through to forming hypotheses and generating theory about the delivery and impacts of SIPC and the interpretation of quantitative outcomes. This analysis approach emphasises theory generation through asking questions about the data and developing emergent lines of thinking to form and question emergent hypotheses. Software for qualitative analysis will facilitate data storage, coding, searching both within and across sites, and participant groups, retrieving data and recording analytical thinking (e.g. NVivo 8). The data are linked with the quantitative data to interpret the change in patients/caregivers of the quantitative outcome measures, their clinical significance, and the impact of SIPC at the three main levels (people and context; processes and tasks; underpinning theory) [81], and to identify ways to enhance SIPC and the processes for wider implementation. Quality appraisal is addressed through procedures to ensure systematic and rigorous attention to analysis and reporting, for example, expert research supervisory review meetings of the data analysis and emerging findings (i.e. qualitative researcher, applicant Evans), attention to deviant case inclusion and analysis at all stages of the research process. Qualitative research software assist comprehensive reporting, auditability, and transparency of the findings.

The views of health professionals in the survey will be analysed using descriptive statistics, comparing the views of different professional groups, sites, expectations and experiences of SIPC. Open comments will be collated and contrasted. Expectations of the service at the start will be compared with the aspects that SIPC affected later. Survey results will be integrated with the trial and qualitative findings to provide greater context about the effects of SIPC on the processes of care, and how it might be working. The findings from qualitative analysis and survey will be integrated with the outcomes analysis to aid better understanding of the intervention and interpretations.

21. Study Steering Committee

A Study Steering Committee (SSC) will be established and appointed in accordance with NIHR guidelines. The SSC will meet at least twice a year and will be responsible for the following:

- overseeing the progress of the study according to KCL and NHS research governance procedures;
- patient safety;
- monitoring adherence to the protocol;
- to review, at regular intervals, relevant information from other sources;
- considering recommendations from the DMEC;
- informing and advising the Trial Management Group on all aspects of the trial.

22. Data Monitoring and Ethics Committee

A Data Monitoring and Ethics Committee (DMEC) will be appointed to monitor the patient safety. The DMEC will consist of independent experts with relevant clinical research experience in palliative care and neurological conditions and a statistician. The DMEC will review on a regular basis accumulating data from the ongoing trial and advise the SSC regarding the safety of the patients, the efficacy endpoints, and whether to continue, modify, or stop the trial. The DMEC will meet at least twice a year.

23. Direct Access to Source Data and Documents

The Investigators will permit trial-related monitoring, audits and REC review by providing the Sponsor(s), and REC direct access to source data and other documents. The investigators will also monitor the intervention in the different sites, using both remote monitoring and site visits. The procedures of this monitoring process are described. In the case of changes to the protocol, the Trial Management Group (TMG) and DMEC will be informed and Ethical approval will be sought. If needed, the funder and sponsor will be informed.

24. Ethics & Regulatory Approvals

The trial will be conducted in compliance with the principles of the Declaration of Helsinki (1996), the principles of GCP and in accordance with all applicable regulatory requirements including but not limited to the Research Governance Framework and the Mental Capacity Act 2005.The protocol and related documents will be submitted to IRAS and considered by ethical committees that can consider vulnerable adults. This protocol and related documents will be submitted for review to King's College London Research Ethics Committee (REC). The Chief Investigator will submit a final report at conclusion of the trial to the funder, the REC and the Sponsor.

25.Quality Assurance

Monitoring of this trial will be to ensure compliance with Good Clinical Practice and scientific integrity will be managed by the study team. We measure the intervention compliance by analysis of the standardised clinical records of the multidisciplinary palliative care teams (MPCTs), and direct observation by the trial co-ordinator. To improve compliance to the intervention and standardisation at centres, we develop a manual of Standard Operating Procedures (SOP) for the intervention and trial conduct, organise regular teleconference calls and visits to the centres, and suggest amendments where required.

26. Data Handling and Management

26.1 Data handling:

The Chief Investigator will act as custodian for the trial data. Patient data will be anonymised and stored in line with the Data Protection Act; the data will be archived in line with Sponsor requirements. Investigators will act to preserve patient confidentiality and will not disclose or reproduce any information by which participants could be identified or traced. Each site in which patients are recruited (and data will be collected) will be approved by ethics. A site agreement will be set up for each site to set out Sponsor/Site/PI responsibilities and a will include data protection/confidentiality clauses.

Data will be managed using the InferMed MACRO database system. An electronic Case Report Form (eCRF) will be created using the InferMed Macro system. This system is regulatory compliant (GCP, 21CRF11, EC

Clinical Trial Directive). The eCRF will be created in collaboration with the trial statisticians and the CI and maintained by the King's Clinical Trials Unit. It will be hosted on a dedicated secure server within KCL. Source data will be entered by authorised staff onto the eCRF with a full audit trail. Study sites will aim to enter eCRFs within 7 days of data collection. Over the course of the trial, the Trial Manager will conduct on-site/central monitoring. The Data Manager/Statistician may identify data fields that should be checked against the source data during site monitoring visits. Data will be single entered and a proportion of the data will be double-checked from source by the research team. Range checks and validations to prevent data entry errors will be programmed into the data entry system. Where there are data queries raised the recruiting centre staff will be responsible for resolving the queries. The Trial Manager will review responses before closing queries.

26.2 Data management:

Database Website Address:
Go to www.ctu.co.uk and click the link to MACRO EDC V4 on the lower right hand side of the screen.

Database passwords:
Database access will be strictly restricted through passwords to the authorised research team. The Trial Manager or delegate will request usernames and passwords from the KCTU. It is a legal requirement that passwords to the eCRF are not shared, and that only those authorised to access the system are allowed to do so. If new staff members join the study, a personalised username and password should be requested via the CI or delegate (e.g Trial Manger) from the KCTU administrator.

Data Handling & Confidentiality/Format of Records:
Data will be handled, computerised and stored in accordance with the Data Protection Act, 1998. Participants will be identified on the study database using a unique code and initials. The investigator will maintain accurate patient records/results detailing observations on each patient enrolled.

Identifiable Data:
All participant contact information data will be stored on spreadsheets within the recruiting site, which will have restricted access from password protected computers. Accrual data uploaded to the UKCRN portfolio database will be anonymised and collated by the CI or delegate to the CLRN. No identifiable data will be entered on the eCRF or transferred to the KCTU.

Data check:
We will monitor the validity of the data that is being collected by research nurses. We will insert some questions in the patient and caregiver questionnaire about whether patients are willing to be contacted about the conduct of the research and the research interview (we will ask them to provide a telephone number for this). Following this, the Trial Manager / Research Associate or Research Nurse at KCL (for the other sites) will telephone a random sample to check how the research went. On data entry all data will also be checked for missing data and feedback will be given to research nurses (including an attempt to try to still collect this data). We will send monthly feedback to research nurses and their managers on the quality of the data.

Main Database:
SAE data will be collected on paper SAE report forms and faxed to the Trial Manager. Summary details of SAEs will be transcribed to adverse event section of the eCRF. For all other data collected, source data worksheets will be prepared for each patient and data will be entered onto the eCRF database. Source data worksheets will be reconciled at the end of the trial with the patient's medical notes in the recruiting centre. Data on the intervention, which may unblind outcome assessors, will be kept separately from the medical notes until the end of the study. Trial related clinical letters will be copied to the medical notes during the trial. The Principal Investigator will provide an electronic signature for each patient Case Record Form once all queries are resolved and immediately prior to database lock. At the end of the study, essential documentation will be archived in accordance with sponsor and local requirements. The retention of study data will be the responsibility of the Chief Investigator. The eCRF will become the formal record of the trial dataset and will be retained for 20 years by the study team as part of the Trial Master File, in the form of a disk with extracts of all data.

Assessments/Data Collection:
Written informed consent must be obtained prior to baseline interview and any other study specific procedures taking place.

Database lock:

The final checking of data and data cleaning will be undertaken by the trial manager, in collaboration with the investigators and trial statistician. After completion of all follow-ups and prompt entry of data, the Trial Manager will review the data and issue queries as necessary. The study site must then answer these queries before the participant's data are locked within the database. After that time, changes will not be made to the database by the research site unless specifically requested by the coordinating site in response to statistician data checks.

At the end of the trial, the site PI will review all the data for each participant and provide electronic sign-off to verify that all the data are complete and correct. At this point, all data will be formally locked for analysis. At the end of the trial, each centre will be supplied with a CD-ROM containing the eCRF data for their centre. This will be filed locally for any future audit.

Data sharing:
Anonymous research data will be stored securely and kept for future analysis. Because this is a unique trial and the first of its kind in the world, it may be that the data will be valuable for many years to come. It might be important to make it available for analysis in Cochrane or other reviews. The data will be kept anonymous on secure access computers, and access will be via written confidentiality and use agreement with Professor Higginson (or her appointed nominee), supervised by or with the involvement of Prof Higginson, or members of the research team. A data sharing plan will be developed. Individual centres will also be able to request the data for analysis, signing the use agreement, providing it is kept on secure locked computers, and they provide verified details of this in advance. The person applying for use of the data will be scrutinized for appropriate eligibility by members of the research team. We will develop a data sharing policy, under guidance of the SSC.

Envisaged data sharing policy:
• The study is registered prior to recruitment on the ISRCTN registry.
• Data are collected, managed and analysed according to the principles of GCP and patients are fully informed of all plans for data sharing within the trial. Participants are asked to consent to sharing of the data (link anonymised) for future prospective research purposes. Archiving is for at least 15 years.
• Analysis is conducted according to a pre-agreed Statistical Analysis Plan.
• Our dissemination plan includes publication in high quality peer reviewed journals, presentation at clinical and research conferences, and production of executive summaries for commissioners, clinicians, policy makers and patients and their carers.
• Data are not released prior to analyses for purposes that might detrimentally affect the trial integrity.
• All requests for data release outside of the planned analyses are considered by the TSC (and when requested following publication by contacting the corresponding author).Any request approved is covered by a written Data Transfer Agreement, detailing limitations of use, transfer to 3rd parties, data storage and acknowledgements.
• The trial protocol and contracts include a publication policy agreed by all collaborators.
• Safety / adverse events data are released to relevant bodies where appropriate to improve patient care
• The results of the trial are notified to participants.

27. Publication Policy

It is intended that the results of the trial will be reported and disseminated at national and international conferences, and in peer-reviewed scientific journals. Data from all centres will be analysed together and published as soon as possible. Co-investigators may not publish data concerning their participants that are directly relevant to questions posed by the trial until the main trial publication has arisen. The main publication and subsequent publications should include the principal investigator and all co-investigators. All authors (including these but also others) should fulfil the criteria as set out by the ICMJE (http://www.icmje.org/recommendations/browse/roles-and-responsibilities/defining-the-role-of-authors-and-contributors.html).

We aim to publish the protocol in a (peer-reviewed) journal and to make it available in accordance with NIHR guidance. Efforts will be made to send a summary of results to participants once they become available. Wider public dissemination will be facilitated by patient and service user representatives, who will form part of the SSC and we will form a separate PPI committee. To maintain the independence of the SSC, DMEC and PPI to the trial, the three committees will be acknowledged but will not be co-author(s) on publications from the trial. Feedback to all local participating sites with a presentation at appropriate research meetings. We will send the funding body progress reports every 6 months in accordance with their guidelines.

28. Insurance / Indemnity

Insurance and indemnity for trial participants and trial staff is covered within the NHS Indemnity Arrangements for clinical negligence claims in the NHS, issued under cover of HSG (96). There are no special compensation arrangements, but trial participants may have recourse through the NHS complaints procedures. The trial will be sponsored by Kings College London, who has taken out an insurance policy to provide indemnity in the event of a successful litigious claim for proven non-negligent harm.

29. Confidentiality agreement

All information disclosed or provided by the Sponsor (or any company/institution acting on their behalf), or produced during the Clinical Trial, including, but not limited to, the Clinical Trial Protocol, the CRFs, the Investigator's Brochure and the results obtained during the course of the Clinical Trial, is confidential. The Investigator or any person under his/her authority agrees to undertake to keep confidential and not to disclose the information to any third party without the prior written approval of the Sponsor. However, the submission of this Clinical Trial Protocol and other necessary documentation to the Ethics Committee (IRB/EC) is expressly permitted, the IRB/EC members having the same obligation of confidentiality. The Sub-Investigators shall be bound by the same obligation as the Investigator. The Investigator shall inform the Sub-Investigators of the confidential nature of the Clinical Trial. The Investigator and the Sub-Investigators shall use the information solely for the purposes of the Clinical Trial, to the exclusion of any use for their own or for a third party's account.

30. References

1. Voltz R, Borasio GD. Palliative therapy in the terminal stage of neurological disease. J Neurol 1997;**244**:S2-S10.

2. Edmonds P, Vivat B, Burman R, et al. 'Fighting for everything': service experiences of people severely affected by multiple sclerosis. Multiple sclerosis 2007;**13**(5):660-7.

3. Edmonds P, Vivat B, Burman R, et al. Loss and change: experiences of people severely affected by multiple sclerosis. Palliative medicine 2007;**21**(2):101-7.

4. Higginson IJ, Hart S, Silber E, et al. Symptom prevalence and severity in people severely affected by multiple sclerosis. Journal of palliative care 2006;**22**(3):158-65.

5. Higginson IJ, Vivat B, Silber E, et al. Study protocol: delayed intervention randomised controlled trial within the Medical Research Council (MRC) Framework to assess the effectiveness of a new palliative care service. BMC palliative care 2006;**5**:7.

6. Dewing J. Participatory research: A method for process consent with persons who have dementia. Dementia 2007;**6**(1):11-25.

7. Department of Health. *Raising the profile of Long Term Conditions Care: a compendium of information.* London: Department of Health, 2008.

8. Bernard S, Aspinal F, Gridley K, et al. Integrated policy making in England for adults with long-term neurological conditions (LTNCs): some preliminary findings from a scoping study. International journal of integrated care 2008;**8**:e60.

9. Fitzpatrick R, Peters M, Doll H, et al. *The needs and experiences of services by individuals with long-term progressive neurological conditions, and their carers. A benchmarking study.* London: Queen's Printer and Controller of HMSO 2010, 2010.

10. Department of Health. *The National Service Framework for Long-term Conditions.* London: Department of Health, 2005.

11. Neurological Alliance. *Neuro numbers*. London: Neurological Alliance in conjunction with the Association of British Neurologists (ABN), Society of British Neurological Surgeons and the Royal College of Nursing (RCN), 2003.

12. Peters M, Fitzpatrick R, Doll H, et al. Patients' experiences of health and social care in long-term neurological conditions in England: a cross-sectional survey. Journal of health services research & policy 2013;**18**(1):28-33.

13. Peters M, Fitzpatrick R, Doll H, et al. The impact of perceived lack of support provided by health and social care services to caregivers of people with motor neuron disease. Amyotrophic lateral sclerosis : official publication of the World Federation of Neurology Research Group on Motor Neuron Diseases 2012;**13**(2):223-8.

14. Morley D, Dummett S, Peters M, et al. Factors influencing quality of life in caregivers of people with Parkinson's disease and implications for clinical guidelines. Parkinson's disease 2012;**2012**:190901.

15. Schoen C, Osborn R, How SK, et al. In chronic condition: experiences of patients with complex health care needs, in eight countries, 2008. Health affairs 2009;**28**(1):w1-16.

16. Schoen C, Osborn R, Doty MM, et al. Toward higher-performance health systems: adults' health care experiences in seven countries, 2007. Health affairs 2007;**26**(6):w717-34.

17. Reilly S, Abell J, Brand C, et al. Case management for people with long-term conditions: impact upon emergency admissions and associated length of stay. Primary health care research & development 2011;**12**(3):223-36.

18. Challis D, Hughes J, Berzins K, et al. Implementation of case management in long-term conditions in England: survey and case studies. Journal of health services research & policy 2011;**16 Suppl 1**:8-13.

19. Salisbury C. Multimorbidity: redesigning health care for people who use it. Lancet 2012;**380**(9836):7-9.

20. Aoun S, McConigley R, Abernethy A, et al. Caregivers of people with neurodegenerative diseases: profile and unmet needs from a population-based survey in South Australia. Journal of palliative medicine 2010;**13**(6):653-61.

21. Higginson IJ, Finlay IG, Goodwin DM, et al. Is there evidence that palliative care teams alter end-of-life experiences of patients and their caregivers? Journal of pain and symptom management 2003;**25**(2):150-68.

22. Harding R, List S, Epiphaniou E, et al. How can informal caregivers in cancer and palliative care be supported? An updated systematic literature review of interventions and their effectiveness. Palliative medicine 2012;**26**(1):7-22.
23. Davies E. *What are the palliative care needs of older people and how might they be met?* Copenhagen: WHO Regional Office for Europe (Health Evidence Network report);, 2004.
24. Chan R, Webster J. End-of-life care pathways for improving outcomes in caring for the dying. Cochrane database of systematic reviews 2010(1):CD008006.
25. Edmonds P, Hart S, Wei G, et al. Palliative care for people severely affected by multiple sclerosis: evaluation of a novel palliative care service. Multiple sclerosis 2010;**16**(5):627-36.
26. Khan F, Ng L, Turner-Stokes L. Effectiveness of vocational rehabilitation intervention on the return to work and employment of persons with multiple sclerosis. Cochrane database of systematic reviews 2009(1):CD007256.
27. Chaudhuri KR, Healy DG, Schapira AH. Non-motor symptoms of Parkinson's disease: diagnosis and management. Lancet neurology 2006;**5**(3):235-45.
28. Higginson IJ, McCrone P, Hart SR, et al. Is short-term palliative care cost-effective in multiple sclerosis? A randomized phase II trial. Journal of pain and symptom management 2009;**38**(6):816-26.
29. Higginson IJ, Gao W, Saleem TZ, et al. Symptoms and quality of life in late stage Parkinson syndromes: a longitudinal community study of predictive factors. PloS one 2012;**7**(11):e46327.
30. Department of Health. *End of Life Care Strategy - promoting high quality care for all adults at the end of life*. London: Department of Health, 2008.
31. Walker H, Anderson M, Farahati F, et al. Resource use and costs of end-of-Life/palliative care: Ontario adult cancer patients dying during 2002 and 2003. Journal of palliative care 2011;**27**(2):79-88.
32. Bergman J, Brook RH, Litwin MS. A call to action: improving value by emphasizing patient-centered care at the end of life. JAMA surgery 2013;**148**(3):215-6.
33. Emanuel EJ, Ash A, Yu W, et al. Managed care, hospice use, site of death, and medical expenditures in the last year of life. Archives of internal medicine 2002;**162**(15):1722-8.
34. McCrone P, Payan CAM, Knapp M, et al. The Economic Costs of Progressive Supranuclear Palsy and Multiple System Atrophy in France, Germany and the United Kingdom. PLoS ONE 2011;**6**(9):e24369.
35. Anderson R. New MRC guidance on evaluating complex interventions. BMJ 2008;**337**:a1937.
36. Epping-Jordan JE, Pruitt SD, Bengoa R, et al. Improving the quality of health care for chronic conditions. Quality & safety in health care 2004;**13**(4):299-305.
37. Higginson IJ, Hart S, Burman R, et al. Randomised controlled trial of a new palliative care service: Compliance, recruitment and completeness of follow-up. BMC palliative care 2008;**7**:7.
38. Solano JP, Gomes B, Higginson IJ. A comparison of symptom prevalence in far advanced cancer, AIDS, heart disease, chronic obstructive pulmonary disease and renal disease. Journal of pain and symptom management 2006;**31**(1):58-69.
39. Higginson IJ, Evans CJ. What is the evidence that palliative care teams improve outcomes for cancer patients and their families? Cancer journal 2010;**16**(5):423-35.
40. Higginson IJ, Costantini M. Dying with cancer, living well with advanced cancer. European journal of cancer 2008;**44**(10):1414-24.
41. Turner-Stokes L, Whitworth D. The National Service Framework for Long Term Conditions: the challenges ahead. Clinical Medicine 2005;**5**:203-06.
42. Temel JS, Greer JA, Muzikansky A, et al. Early palliative care for patients with metastatic non-small-cell lung cancer. NEnglJ Med 2010;**363**(8):733-42.
43. Gruenewald DA, Higginson IJ, Vivat B, et al. Quality of life measures for the palliative care of people severely affected by multiple sclerosis: a systematic review. Multiple sclerosis 2004;**10**(6):690-704.
44. Gomes B, McCrone P, Hall S, et al. Cognitive interviewing of bereaved relatives to improve the measurement of health outcomes and care utilisation at the end of life in a mortality followback survey. Supportive care in cancer : official journal of the Multinational Association of Supportive Care in Cancer 2013;**21**(10):2835-44.
45. Hoehn MM, Yahr MD. Parkinsonism: onset, progression and mortality. Neurology 1967;**17**(5):427-42.
46. Watson JM, Torgerson DJ. Increasing recruitment to randomised trials: a review of randomised controlled trials. BMC medical research methodology 2006;**6**:34.

47. Davies K, Collerton JC, Jagger C, et al. Engaging the oldest old in research: lessons from the Newcastle 85+ study. BMC geriatrics 2010;**10**:64.
48. Jones L, Harrington J, Scott S, et al. CoMPASs: IOn programme (Care Of Memory Problems in Advanced Stages of dementia: Improving Our Knowledge): protocol for a mixed methods study. BMJ open 2012;**2**(6).
49. Scott S, Jones L, Blanchard MR, et al. Study protocol: the behaviour and pain in dementia study (BePAID). BMC geriatrics 2011;**11**:61.
50. Department of Health. Mental Capacity Act. London: Department of Health, 2005.
51. Mathie E, Goodman C, Crang C, et al. An uncertain future: the unchanging views of care home residents about living and dying. Palliat Med 2012;**26**(5):734-43.
52. Rees E, Hardy J. Novel consent process for research in dying patients unable to give consent. Bmj 2003;**327**(7408):198.
53. Dobson C. Conducting research with people not having the capacity to consent to their participation: A practical guide for researchers. In: Party MCAW, ed. Leicester: The British Psychological Society, 2008:68.
54. Division DSDaB. Guidance on nominating a consultee for research involving adults who lack capacity to consent, 2008.
55. Farquhar M, Preston N, Evans CJ, et al. Mixed methods research in the development and evaluation of complex interventions in palliative and end-of-life care: report on the MORECare consensus exercise. J Palliat Med 2013;**16**(12):1550-60.
56. Kutner JS, Bryant LL, Beaty BL, et al. Symptom Distress and Quality-of-Life Assessment at the End of Life: The Role of Proxy Response. J Pain Symptom Manage 2006;**32**(4):300-10.
57. Gysels M, Evans CJ, Lewis P, et al. MORECare research methods guidance development: recommendations for ethical issues in palliative and end-of-life care research. Palliat Med 2013;**27**(10):908-17.
58. Gysels M, Shipman C, Higginson IJ. Is the qualitative research interview an acceptable medium for research with palliative care patients and carers? BMC medical ethics 2008;**9**:7.
59. Gysels M, Shipman C, Higginson IJ. "I will do it if it will help others:" motivations among patients taking part in qualitative studies in palliative care. Journal of pain and symptom management 2008;**35**(4):347-55.
60. Gysels MH, Evans C, Higginson IJ. Patient, caregiver, health professional and researcher views and experiences of participating in research at the end of life: a critical interpretive synthesis of the literature. BMC medical research methodology 2012;**12**:123.
61. Higginson IJ, Costantini M, Silber E, et al. Evaluation of a new model of short-term palliative care for people severely affected with multiple sclerosis: a randomised fast-track trial to test timing of referral and how long the effect is maintained. Postgraduate medical journal 2011;**87**(1033):769-75.
62. Peters M, Jenkinson C, Doll H, et al. Carer quality of life and experiences of health services: a cross-sectional survey across three neurological conditions. Health and quality of life outcomes 2013;**11**:103.
63. O'Brien MR, Whitehead B, Jack BA, et al. The need for support services for family carers of people with motor neurone disease (MND): views of current and former family caregivers a qualitative study. Disability and rehabilitation 2012;**34**(3):247-56.
64. Bradley F, Wiles R, Kinmonth AL, et al. Development and evaluation of complex interventions in health services research: case study of the Southampton heart integrated care project (SHIP). The SHIP Collaborative Group. BMJ 1999;**318**(7185):711-15.
65. Goodman C, Evans C. Focus Groups. In: Gerrish K, Lacey A, eds. The Research Process in Nursing, 2010.
66. Sleeman KE, Higginson IJ. A psychometric validation of two brief measures to assess palliative need in patients severely affected by multiple sclerosis. Journal of pain and symptom management 2013;**46**(3):406-12.
67. Bausewein C, Le Grice C, Simon S, et al. The use of two common palliative outcome measures in clinical care and research: a systematic review of POS and STAS. Palliative medicine 2011;**25**(4):304-13.
68. Harding R, Simon ST, Benalia H, et al. The PRISMA Symposium 1: outcome tool use. Disharmony in European outcomes research for palliative and advanced disease care: too many tools in practice. Journal of pain and symptom management 2011;**42**(4):493-500.

69. Higginson IJ, Simon ST, Benalia H, et al. Republished: which questions of two commonly used multidimensional palliative care patient reported outcome measures are most useful? Results from the European and African PRISMA survey. Postgraduate medical journal 2012;**88**(1042):451-7.
70. Saleem TZ, Higginson IJ, Chaudhuri KR, et al. Symptom prevalence, severity and palliative care needs assessment using the Palliative Outcome Scale: A cross-sectional study of patients with Parkinson's disease and related neurological conditions. Palliative medicine 2012:Dec 3. [Epub ahead of print].
71. Longworth L, Rowen D. Mapping to Obtain EQ-5D Utility Values for Use in NICE Health Technology Assessments. Value in health : the journal of the International Society for Pharmacoeconomics and Outcomes Research 2013;**16**(1):202-10.
72. Al-Janabi H, Flynn T, Coast J. Development of a self-report measure of capability wellbeing for adults: the ICECAP-A. Quality of Life Research 2012;**21**(1):167-76.
73. Beaumont C, Nekolaichuk C. FAMCARE and FAMCARE-2 Guidelines. Edmonton: Covenant Health, 2012:23.
74. Tuck KK, Brod L, Nutt J, et al. Preferences of Patients With Parkinson's Disease for Communication About Advanced Care Planning. The American journal of hospice & palliative care 2013.
75. Beecham J, Knapp M. Costing psychiatric interventions. In: G. T, ed. Measuring mental health needs 2nd edition. London Gaskell, 2001:200-24.
76. Higginson IJ, Gao W, Jackson D, et al. Short-form Zarit Caregiver Burden Interviews were valid in advanced conditions. Journal of clinical epidemiology 2010;**63**(5):535-42.
77. Salyers MP, Bosworth HB, Swanson JW, et al. Reliability and validity of the SF-12 health survey among people with severe mental illness. Medical care 2000;**38**(11):1141-50.
78. Gomes B, McCrone P, Hall S, et al. Variations in the quality and costs of end-of-life care, preferences and palliative outcomes for cancer patients by place of death: the QUALYCARE study. BMC cancer 2010;**10**:400.
79. Higginson IJ, C E, Grand G, et al. Evaluating complex interventions in End of Life Care: the MORECare Statement on good practice generated by a synthesis of transparent expert consultations and systematic reviews. BMC Medicine 2013:In Press.
80. Coffey A, Atkinson P. *Making sense of qualitative data: complementary research strategies*. California: Sage, 1996.
81. Bradley N, Sweeney K, Waterfield M. The health of their nation: how would citizens develop England's health strategy? BrJ GenPract 1999;**49**(447):801-05.

31. Financial Aspects

Funding to conduct the trial is provided by NIHR HS & DR (Ref No. 12/130/47).

32. Declaration of Interest

The PI, CO-PI and Principal Investigators for all sites declare that they have no financial or other competing interests for this study.

33. Signatures

Chief Investigator Date: 28/08/2018

IRENE HIGGINSON

Statistician (if applicable) Date: 28/08/2018

GAO WEI

34. Appendices

34.1 Management Structure for the Project

The management structure for the project is shown in the following diagram:

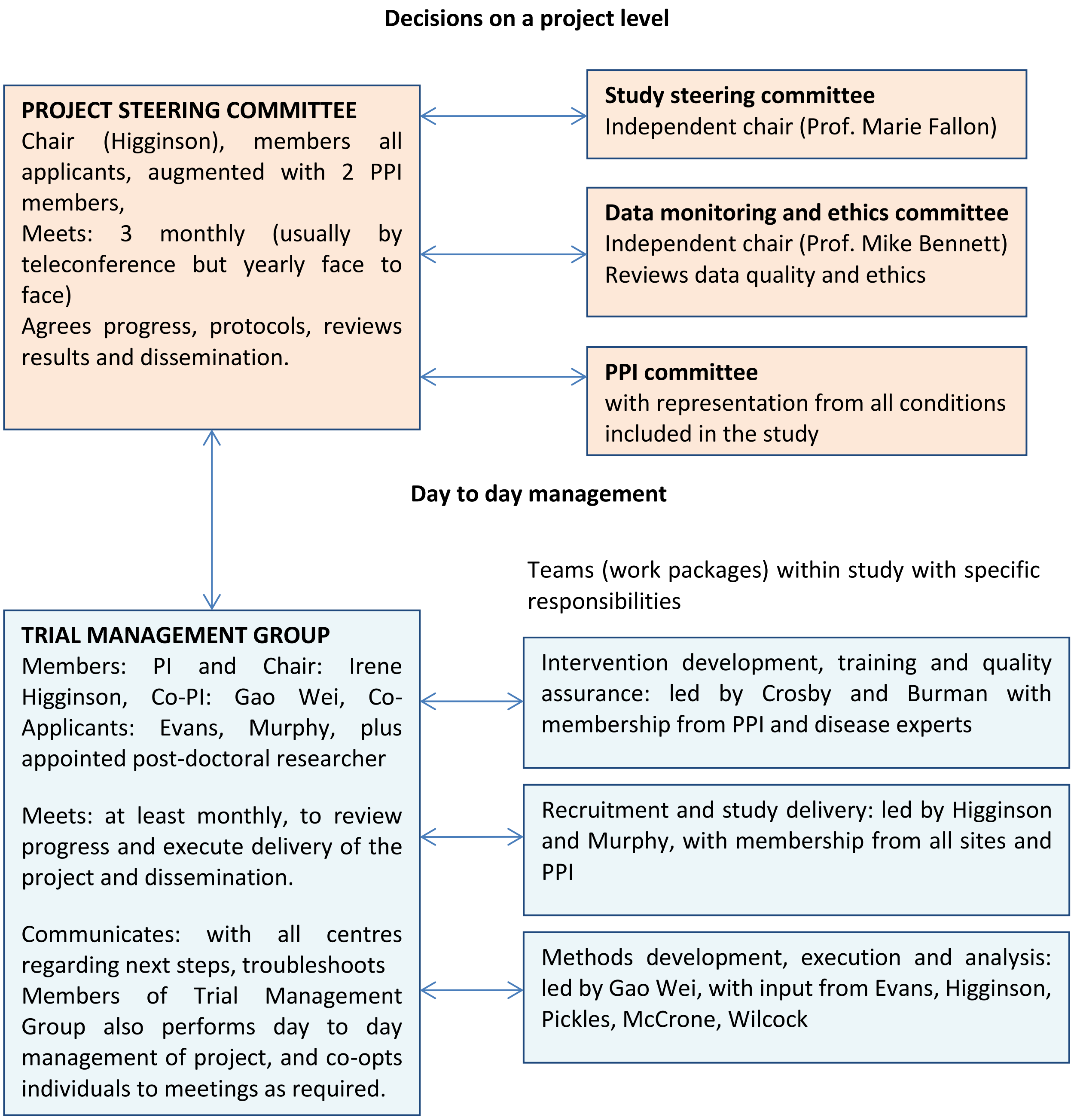

34.2 Publications from Phase II trial and related studies

1. Higginson IJ, Vivat B, Silber E, Saleem T, Burman R, Hart S, Edmonds P. Study protocol: delayed intervention randomised controlled trial within the Medical Research Council (MRC) Framework to assess the effectiveness of a new palliative care service. BMC Palliat Care. 2006 Oct 2;5:7.
2. Edmonds P, Hart S, Wei Gao, Vivat B, Burman R, Silber E, Higginson IJ. Palliative care for people severely affected by multiple sclerosis: evaluation of a novel palliative care service. Mult Scler. 2010 May;16(5):627-36. doi: 10.1177/1352458510364632. Epub 2010 Mar 19.
3. Higginson IJ, McCrone P, Hart SR, Burman R, Silber E, Edmonds PM. Is short-term palliative care cost-effective in multiple sclerosis? A randomized phase II trial. J Pain Symptom Manage. 2009 Dec;38(6):816-26. doi: 10.1016/j.jpainsymman.2009.07.002. Epub .
4. Higginson IJ, Hart S, Burman R, Silber E, Saleem T, Edmonds P. Randomised controlled trial of a new palliative care service: Compliance, recruitment and completeness of follow-up. BMC Palliat Care. 2008 May 28;7:7. doi: 10.1186/1472-684X-7-7.
5. Higginson IJ, Gao W, Saleem TZ, Chaudhuri KR, Burman R, McCrone P, Leigh PN. Symptoms and quality of life in late stage Parkinson syndromes: a longitudinal community study of predictive factors. PLoS One. 2012;7(11):e46327. doi: 10.1371/journal.pone.0046327. Epub 2012 Nov 7.
6. Higginson IJ, Booth S. The randomized fast-track trial in palliative care: role, utility and ethics in the evaluation of interventions in palliative care? Palliat Med. 2011 Dec;25(8):741-7. doi: 10.1177/0269216311421835. Epub 2011 Oct 12.
7. Higginson IJ, Costantini M, Silber E, Burman R, Edmonds P. Evaluation of a new model of short-term palliative care for people severely affected with multiple sclerosis: a randomised fast-track trial to test timing of referral and how long the effect is maintained. Postgrad Med J. 2011 Nov;87(1033):769-75. doi: 10.1136/postgradmedj-2011-130290. Epub 2011 Oct 6.
8. Edmonds P, Vivat B, Burman R, Silber E, Higginson IJ. 'Fighting for everything': service experiences of people severely affected by multiple sclerosis. Mult Scler. 2007 Jun;13(5):660-7. Epub 2007 Feb 9.
9. Edmonds P, Vivat B, Burman R, Silber E, Higginson IJ. Loss and change: experiences of people severely affected by multiple sclerosis. Palliat Med. 2007 Mar;21(2):101-7.
10. Smith W, McCrone P, Goddard C, Gao W, Burman R, Jackson D, Higginson I, Silber E, Koffman J. Comparisons of Costs between Black Caribbean and White British Patients with Advanced Multiple Sclerosis in the UK. Mult Scler Int. 2014;2014:613701. doi: 10.1155/2014/613701. Epub 2014 Feb 5.
11. Sleeman KE, Ho YK, Verne J, Glickman M, Silber E, Gao W, Higginson IJ; GUIDE_Care Project. Place of death, and its relation with underlying cause of death, in Parkinson's disease, motor neurone disease, and multiple sclerosis: a population-based study. Palliat Med. 2013 Oct;27(9):840-6. doi: 10.1177/0269216313490436. Epub 2013 Jun 4.
12. Sleeman KE, Higginson IJ. A psychometric validation of two brief measures to assess palliative need in patients severely affected by multiple sclerosis. J Pain Symptom Manage. 2013 Sep;46(3):406-12. doi: 10.1016/j.jpainsymman.2012.08.007. Epub 2012 Nov 27.
13. Koffman J, Gao W, Goddard C, Burman R, Jackson D, Shaw P, Barnes F, Silber E, Higginson IJ. Progression, symptoms and psychosocial concerns among those severely affected by multiple sclerosis: a mixed-methods cross-sectional study of Black Caribbean and White British people. PLoS One. 2013 Oct 2;8(10):e75431. doi: 10.1371/journal.pone.0075431. eCollection 2013.
14. Price A, Rayner L, Okon-Rocha E, Evans A, Valsraj K, Higginson IJ, Hotopf M. Antidepressants for the treatment of depression in neurological disorders: a systematic review and meta-analysis of randomised controlled trials. J Neurol Neurosurg Psychiatry. 2011 Aug;82(8):914-23. doi: 10.1136/jnnp.2010.230862. Epub 2011 May 10. Review.

Manual for provision of Short-term Integrated Palliative Care Services (SIPC) in the OPTCARE Neuro Trial

Evaluation of the clinical and cost-effectiveness of Short-term Integrated Palliative Care Services (SIPC) to OPTimise CARE for people with advanced long-term Neurological conditions

This trial involves centres collaborating to give a similar 'early' short term palliative care service to patients severely affected by neurological conditions. It has been designed by palliative care and neurology clinicians led by Professor Irene Higginson from King's College London, the centres and lead applicants are listed below,

London

Professor Irene J Higginson	Chief Investigator
Dr Gao Wei	Co-CI, Trial Statistician
Dr Rachel Burman	Co-applicant, Lead London component
Dr Catherine Evans	Co-applicant, Lead qualitative component
Dr Fliss Murtagh	Co-applicant
Dr Diana Jackson	Co-applicant
Professor Andrew Pickles	Co-applicant
Professor Paul McCrone	Co-applicant
Professor Ammar Al-Chalabi	Co-applicant
Professor Ray Chaudhuri	Co-applicant
Professor Matthew Hotopf	Co-applicant
Dr Eli Silber	Co-applicant
Dr Cynthia Benz	Co-applicant, Patient Representative

Brighton

Professor P Nigel Leigh	Co-applicant
Dr. Fiona Lindsay	Lead Brighton component

Liverpool

Professor Carolyn A Young	Co-applicant, Lead Liverpool component

Nottingham

Dr Vincent Crosby	Co-applicant Lead Nottingham component
Dr Andrew Wilcock	Co-applicant

Cardiff

Dr Anthony Byrne	Co-Applicant, Lead Welsh Centre

Contents

1. Introduction to this manual
2. Introduction to the trial
3. Timelines for the intervention and outline of response after referral and visits
4. Topics to be covered in the assessment
5. Symptom management and drug recommendations
6. Standard Recording of clinical information using the usual recording method for each Multiprofessional Palliative Care Team
7. Process data
8. Quality assurance

1. Introduction to this manual

This manual has been written to support the specialist palliative care clinicians who are delivering the intervention in the OPTCARE Neuro trial.

It is a reference guide for the clinicians to help provide a consistent approach to assessing patients and families, collecting information and delivering the short-term integrated palliative care (SIPC). It has been developed by the Trial Management Group with input from all of the applicants on the trial.

The manual gives an introduction to the trial and an overview of the key components of the trial. It sets out to clearly define the roles and responsibilities of the multiprofessional palliative care team (MPCT) in the trial. It covers the timescales for responding to a patient recruited into the trial.

The intervention is described in detail with the core elements that would be covered in an assessment of a patient being seen by a specialist palliative care health professional highlighted. The manual sets out the minimum standards for recording the clinical intervention with the paper based documentation from Nottingham shown as an example (Appendix 1). It is recognised that existing clinical teams will have their own paper based or electronic systems for recording clinical information. **Each clinical service will need to ensure that the method they use for recording clinical information will allow them to capture and report the minimum standards set out in this manual.** Guidelines for managing common symptoms and suggested drug treatments are also covered in this manual. The intervention, guidelines and suggested drug treatments are designed to provide a common framework to improve consistency across the recruiting sites. It is also important to note that palliative care is focussed on responding and adapting to individual patient and family needs and concerns. This approach to individual needs is also an important part of the intervention, however it must be documented. This will enable others to learn from our experience. The intervention is being delivered by highly skilled specialist palliative care health professionals who are expert in the assessment and management of the palliative care needs of patients. **As such the guidelines and suggested drug treatments are to support but not substitute the clinical expertise of the health professional.**

This manual complements the trial protocol document which is the definitive trial document. Please refer to the protocol document for further details related to trial procedure.

2. Introduction to the trial

Why do this trial?

Although palliative care has been recommended for patients severely affected by Long Term Neurological Conditions (LTNCs) there are many questions as to how it should be delivered. Much of the evaluation of palliative care services has occurred among cancer patients, with little research among those with neurological conditions. Research is therefore needed to test palliative care for patients with LTNCs. It is especially important to understand if palliative care is useful earlier than the last few days of life – as is recommended by some clinicians. It is also important to find out if palliative care services are effective or cost-effective, what components of care are most valued by patients and caregivers and when referrals to palliative care should occur. Here we are proposing a slightly amended way of offering specialist palliative care in severe neurological illness, with short-term palliative care – usually of only a few visits and opportunities for later referral. The hypothesis is that this may be more sustainable than providing specialist care in full throughout the whole course of illness. With shortages of professionals and demands to support more patients we need to know if this approach will offer benefits for patients and families, what these are, and importantly if there are any harms or downsides to offering short-term early palliative care.

Which patients will be eligible for the trial?
The inclusion criteria for the trial are as follows:

<u>Patients</u>

1) Adults (aged 18 years or over) severely affected by advanced or progressive stages of the long-term neurological conditions (LTNCs) of either*:

- Multiple Sclerosis (MS) - patients with either aggressive relapsing disease with rapid development of fixed disability or those with advanced primary or secondary progressive disease, often with limitation in a number of areas including gait and upper limb function. We do not define referral based on disability but would expect most patients to have an Expanded Disability Status Scale (EDSS) of at least 7.5.

- Parkinsonism & related disorders (PRDs) i.e.

  Idiopathic Parkinson's Disease(IPD), Hoehn and Yahr (H&Y) stages 4-5
  OR
  Progressive Supranuclear Palsy(PSP) Hoehn and Yahr (H&Y) stages 3-5
  OR
  Multiple System Atrophy(MSA) - Hoehn and Yahr (H&Y) stages 3-5

- Motor Neurone Disease (MND) all stages

AND

2) who are deemed (by referring/usual care clinicians) to have:

- an unresolved symptom (e.g. pain or another symptom) which has not responded to usual care
- AND at least one of the following: unresolved other symptom (e.g. breathlessness, nausea / vomiting, spasticity, fatigue); cognitive problems; complex psychological (depression, anxiety, loss, family concerns), communication/information problems and/or complex social needs.

AND

3) who are able to give informed consent^ OR where their capacity can be enhanced^ (e.g. with information) so they can give informed consent OR where a personal consultee^ can be identified and approached to give a opinion on whether or not the patient should be included in the study

AND

4) are living in the catchment area of the Short-term Integrated Palliative Care Service (SIPC)

We expect patients to be in the advanced or progressive stages of disease. They may be living at home (most common), in a nursing home or in hospital at the time of recruitment.

Caregivers

1) Adults (aged 18 years or over) identified by the patient as the person closest to them, usually a family member, close friend, informal caregiver or neighbour

AND

2) able to give informed consent and to complete the questionnaires

Notes:
* Diagnosis must have been established by a specialist neurological assessment

^ When a person is thought to be unable to give informed consent the procedures and code of the Mental Capacity Act (2005) should be followed

Exclusion Criteria
Patients

Patients who meet the inclusion criteria but:

1) are already receiving specialist palliative care or have received specialist palliative care in the last 6 months
2) lack capacity and have no family member, friend or informal caregiver who is willing and available to complete questionnaires about their own and the patient's symptoms and circumstances

How will patients be identified?
Patients will be identified by neurologists and neurology nurse specialists. We will ask the identifying clinicians to give written information to patients about the study and SIPC and if patients are interested and willing, the clinician will complete a standard referral form to send to the local research team. The local research team will then contact the patient to:

- explain the trial
- arrange to send further written information
- arrange to visit so that they can answer any questions
- obtain consent
- conduct the baseline interview and questionnaires

If patients consent to their caregiver being contacted, caregivers are approached to self-complete questionnaires.

Procedures
Once the patient has given consent they will be randomised by the Trial Manager at King's (Nilay Hepgul). The Trial Manager will inform the patient about the outcome of this randomisation and when they can expect to hear from the MPCT. The Trial Manager will contact you as the MPCT once a patient is supposed to receive the SIPC intervention, with the details of the patient. Patients should not be able to receive SIPC unless they have been referred through the study. We have completed mapping which shows that patients with LTNCs don't usually receive specialist palliative care, so we are not denying patients anything that they currently receive.

You are encouraged to have no direct contact with the patient until these details have been received nor to take referrals for the trial from any other source.

The patients will be assigned to receive either Short Term Integrated Palliative Care (SIPC) or Standard Care.

Short Term Integrated Palliative Care (SIPC)
This group of patients will be offered the short term integrated palliative care service delivered by a MPCT straight away.

Standard Care
This is the control group and for the next twelve weeks this group of patients will continue with their usual care from their neurologist, specialist nurse and community teams. At the end of the twelve week period you will be notified the details of these patients and they will be offered the short term integrated palliative care intervention. You should not offer specialist palliative care to this group before this time, and their details will not have been sent to you by the Trial Manager.

3. Timelines for the intervention and outline of response after referral and visits

The length of intervention is usually 6-8 weeks from referral. Specialist palliative care is always an individualised service, responding to patients needs and SIPC is intended to be the same. Therefore, there should be some flexibility to adjust to patient and families individual needs and requirements, and some patients will need quicker support after the first or second visit. Each visit is expected to last 60-90 minutes. A small number (based on previous experience, 10%) will need continuing palliative care support and this will be provided on a needs basis. It is anticipated that the teams will have usually put in place the systems for improved care and have resolved immediate issues. The majority of patients and caregivers will be able to be discharged with no formal follow up after the third visit. A small number of patients and caregivers may also need only 2 visits and phone contact.

First telephone contact (made within 2 working days of referral, sooner if urgent, if longer than this time reasons recorded).

Date contact made and the time it takes for this telephone contact to be recorded on the clinical contact form (Appendix 2). Initial introductions, explain role of team, review of symptoms and concerns of patient and if appropriate family, limited telephone palliative care assessment. Agree date and time for first visit. Location can be at patients home or in other location (e.g. hospital if patients is attending outpatients or elsewhere) as mutually agreed. Consultation with colleagues in palliative care team and contact with any other relevant professionals (e.g. neurology, primary care, rehabilitation) as required for background information

Visit 1
Visit from key worker, a specialist in palliative care (nurse, doctor or other) within 5 working days from first telephone contact. Record the time taken for the visit including travel on the usual clinical record form.

Comprehensive palliative care assessment (for more detail see section 4) recorded on the usual clinical record form covering:

- Completion of IPOS Neuro
- Symptom control and management
- Continuity and co-ordination of care, access to services
- Psychosocial needs
- Information / communication needs
- Advance care planning
- Assessment of caregiver
- Generate an agreed personalised problem list and action plan including agreement of plan for next visit/contact
- Patient/family given information about the SIPC and what is provided and given contact details
- Patient is discussed in Multi-disciplinary team meeting using the MDT proforma (Appendix 3). When this is completed, this is then sent to the patient and all relevant health professionals.

After 1st visit
Review problem list/plan from first visit with multi-professional team & liaise with relevant health, social and voluntary sector professions to ensure other services are provided or other needs met.

- Contact with patient/ family as agreed in plan and as required. All contacts made with the patient/carer or with health professionals outside the study visits need to be documented in the clinical contact form (Appendix 2).
- Contact to confirm second visit.

Visit 2
This will usually be within two weeks of visit 1
Visit from key worker or other relevant team member- Record the time taken for the visit including travel on the usual clinical record form.

Review outcomes from actions already taken.
Reassessment covering areas as for visit 1.
If immediate issues have resolved, explore needs and wishes for advance care planning. Revise personalised plan and actions.
Discuss plan for discharge.

After 2nd visit
- Liaise with relevant health professionals and provide information, education, advice and co-ordination to health professions as required. Re-review personalised problem list/plan with multi-professional team and complete MDT proforma. When this is completed, this is then sent to the patient and all relevant health professionals.
- Contact with patient/ family as agreed in plan and as required. All contacts made with the patient/carer or with health professionals in relation to the patient outside the study visits need to be documented on the clinical contact form (Appendix 2).
- Contact to confirm third visit

Visit 3
This will usually be 3-4 weeks after visit 2
Visit from key worker or other relevant team member- Record the time taken for the visit including travel on the usual clinical record form.

Review outcomes from actions already taken
Reassessment, revise plan
Reassessment repeated as required

Discharge
- Final Review with multi-professional team & liaison with relevant health professions to ensure services in place. Re-review personalised problem list/plan with multi-professional team and complete MDT proforma. When this is completed, this is then sent to the patient and all relevant health professionals.

4. Topics to be covered in the Assessment

We would like you to use the IPOS Neuro staff questionnaire shown below to ascertain the problems that the patient identifies as important to them.

The research nurses will be using the patient version of IPOS Neuro during the research visits this differs very slightly from the staff version.

We are asking the clinical teams to use the staff version (as described in the protocol) as a way of making sure everything is covered during the clinical review

Patient name: …………………………..
Patient DOB: …………………………
Date completed: ……………………..

IPOS Neuro Staff Version

www.pos-pal.org

Q1. What have been the patient's main problems or concerns over the past 3 days?

1...

2...

3. ....

Q2. Please tick one box that best describes how the patient has been affected by each of the following symptoms over the past 3 days?

	Not at all	Slightly	Moderately	Severely	Over-whelmingly	*Cannot assess (e.g. unconscious)*
**Pain**	0 ☐	1 ☐	2 ☐	3 ☐	4 ☐	☐
**Shortness of breath**	0 ☐	1 ☐	2 ☐	3 ☐	4 ☐	☐
**Nausea (feeling like you are going to be sick)**	0 ☐	1 ☐	2 ☐	3 ☐	4 ☐	☐
**Vomiting (being sick)**	0 ☐	1 ☐	2 ☐	3 ☐	4 ☐	☐
**Poor appetite**	0 ☐	1 ☐	2 ☐	3 ☐	4 ☐	☐
**Constipation**	0 ☐	1 ☐	2 ☐	3 ☐	4 ☐	☐
**Sore or dry mouth**	0 ☐	1 ☐	2 ☐	3 ☐	4 ☐	☐
**Drowsiness**	0 ☐	1 ☐	2 ☐	3 ☐	4 ☐	☐
**Poor mobility**	0 ☐	1 ☐	2 ☐	3 ☐	4 ☐	☐
**Spasms**	0 ☐	1 ☐	2 ☐	3 ☐	4 ☐	☐

	Not at all	Slightly	Moderately	Severely	Over-whelmingly	*Cannot assess (e.g. unconscious)*
**Fatigue or lack of energy**	0 ☐	1 ☐	2 ☐	3 ☐	4 ☐	☐
**Problems swallowing**	0 ☐	1 ☐	2 ☐	3 ☐	4 ☐	☐
**Feeling sleepy**	0 ☐	1 ☐	2 ☐	3 ☐	4 ☐	☐
**Difficulty in sleeping**	0 ☐	1 ☐	2 ☐	3 ☐	4 ☐	☐
**Difficulty with bowel control**	0 ☐	1 ☐	2 ☐	3 ☐	4 ☐	☐
**Difficulty controlling urine**	0 ☐	1 ☐	2 ☐	3 ☐	4 ☐	☐
**Pressure Sores**	0 ☐	1 ☐	2 ☐	3 ☐	4 ☐	☐
**Problems using your arms**	0 ☐	1 ☐	2 ☐	3 ☐	4 ☐	☐
**Problems using your legs**	0 ☐	1 ☐	2 ☐	3 ☐	4 ☐	☐
**Difficulty communicating**	0 ☐	1 ☐	2 ☐	3 ☐	4 ☐	☐
**Dribbling of Saliva**	0 ☐	1 ☐	2 ☐	3 ☐	4 ☐	☐
**Falls**	0 ☐	1 ☐	2 ☐	3 ☐	4 ☐	☐
**Hallucinations**	0 ☐	1 ☐	2 ☐	3 ☐	4 ☐	☐
**Mouth Problems**	0 ☐	1 ☐	2 ☐	3 ☐	4 ☐	☐
**Loss or change in your ability to taste or smell**	0 ☐	1 ☐	2 ☐	3 ☐	4 ☐	☐
**Unexplained change in weight (not due to change in diet).**	0 ☐	1 ☐	2 ☐	3 ☐	4 ☐	☐

	Not at all	Slightly	Moderately	Severely	Over-whelmingly	*Cannot assess (e.g. unconscious)*
**Problems remembering things that have happened recently or forgetting to do things.**	0☐	1☐	2☐	3☐	4☐	☐
**Loss of interest in what is happening around you or in doing things.**	0☐	1☐	2☐	3☐	4☐	☐
**Difficulty concentrating or staying focused.**	0☐	1☐	2☐	3☐	4☐	☐
**Feeling less interested in sex or more interested in sex.**	0☐	1☐	2☐	3☐	4☐	☐
**Finding it difficult to have sex when you try.**	0☐	1☐	2☐	3☐	4☐	☐
**Feeling light-headed, dizzy or weak standing from sitting or lying.**	0☐	1☐	2☐	3☐	4☐	☐
**Excessive sweating**	0☐	1☐	2☐	3☐	4☐	☐
**Double vision**	0☐	1☐	2☐	3☐	4☐	☐

**Please list any other symptoms and tick one box to show how you feel each of these symptoms has affected the patient over the past 3 days.**						
**1.**	0☐	1☐	2☐	3☐	4☐	☐
**2.**	0☐	1☐	2☐	3☐	4☐	☐
**3.**	0☐	1☐	2☐	3☐	4☐	☐

Over the past 3 days:

	Not at all	*Occasionally*	*Sometimes*	*Most of the time*	*Always*	*Cannot assess (e.g. unconscious)*
**Q3. Has s/he been feeling anxious or worried about his/her illness or treatment?**	0☐	1☐	2☐	3☐	4☐	☐
**Q4. Have any of his/her family or friends been anxious or worried about the patient?**	0☐	1☐	2☐	3☐	4☐	☐
**Q5. Do you think s/he felt depressed?**	0☐	1☐	2☐	3☐	4☐	☐
	Always	*Most of the time*	*Sometimes*	*Occasionally*	*Not at all*	*Cannot assess (e.g. unconscious)*
**Q6. Do you think s/he felt at peace?**	0☐	1☐	2☐	3☐	4☐	☐
**Q7. Has the patient been able to share how s/he is feeling with his/her family or friends as much as s/he wanted?**	0☐	1☐	2☐	3☐	4☐	☐
**Q8. Has the patient had as much information as s/he wanted?**	0☐	1☐	2☐	3☐	4☐	☐
	Problems addressed/ No problems	*Problems mostly addressed*	*Problems partly addressed*	*Problems hardly addressed*	*Problems not addressed*	*Cannot assess (e.g. unconscious)*
**Q9. Have any practical problems resulting from his/her illness been addressed? (such as financial or personal)**	0☐	1☐	2☐	3☐	4☐	☐

In addition to using the questionnaire to identify problems, systematically cover the following:

A. History to include illness understanding/education

B. Decision making

Inquire about mode and preferences for decision making
Assist with treatment decision making if necessary
Planning for future care (ACP) preferences
Wishes for place of care and death

C. Information

Ascertain patients understanding of illness and prognosis.
Clarify information needs.
Provide patients/caregivers with information related to:

- their specific disease
- palliative care and SIPC (including goals)
- planning your future care if appropriate

D. Coping with life threatening illness and uncertainty

Patient
Family/family caregivers
Dealing with loss

E. Social/Family

How are family managing?

F. Rehabilitation

Practical needs at home- ADLs, rehabilitation input

G. Medication

Medication review
Difficulties with adherence/self-administration/timing
Note new medications prescribed

H. Referrals

Identify care plan for future appointments
Indicate referrals to other care providers

5. Symptom management and drug recommendations

On the following pages we have reproduced one set of evidence-based guidelines for clinical management titled:

‘Long-term neurological conditions: management at the interface between neurology, rehabilitation and palliative care’.

These are a set of National guidelines from 2008 and we have included them to help palliative care teams who may be less familiar with managing symptoms in long term neurological conditions.

We are aware that guidelines will sometimes make recommendations and suggestions that lack robust evidence to support them and as such guidelines and suggested drug treatments are to support but not substitute the clinical expertise of the health professional.

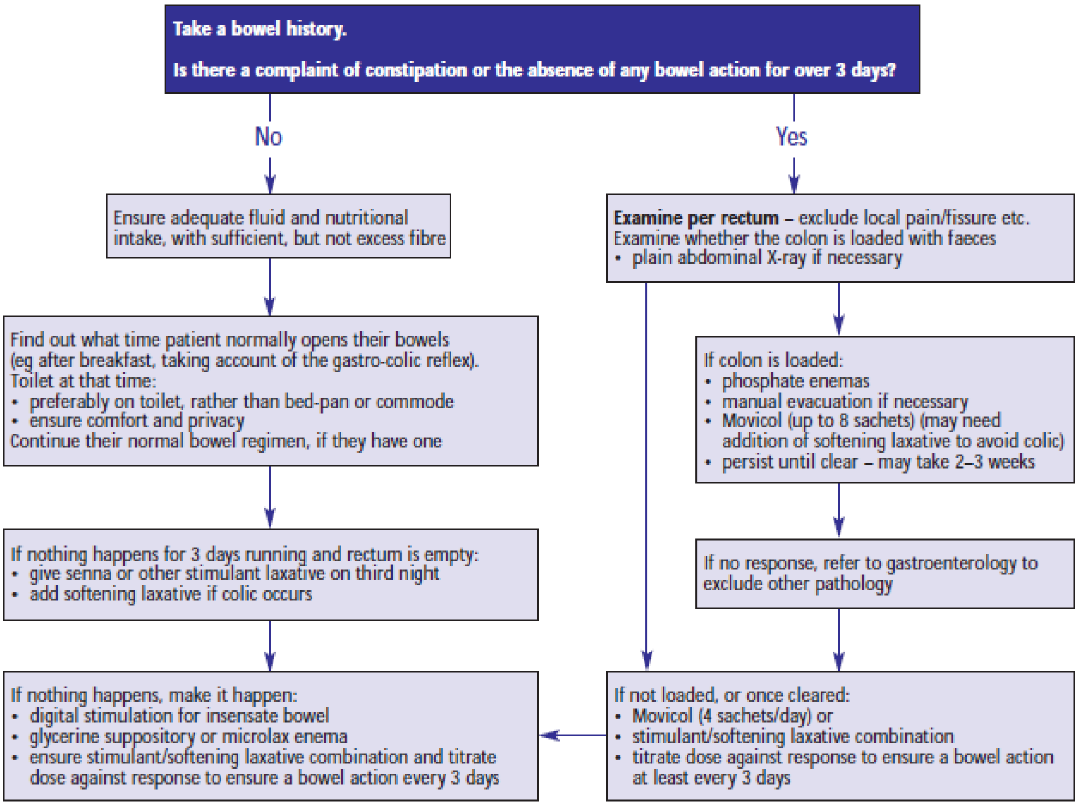

Fig. A1. Bowel management in patients with long-term neurological conditions.
Movicol = polyethlene glycol 3350.

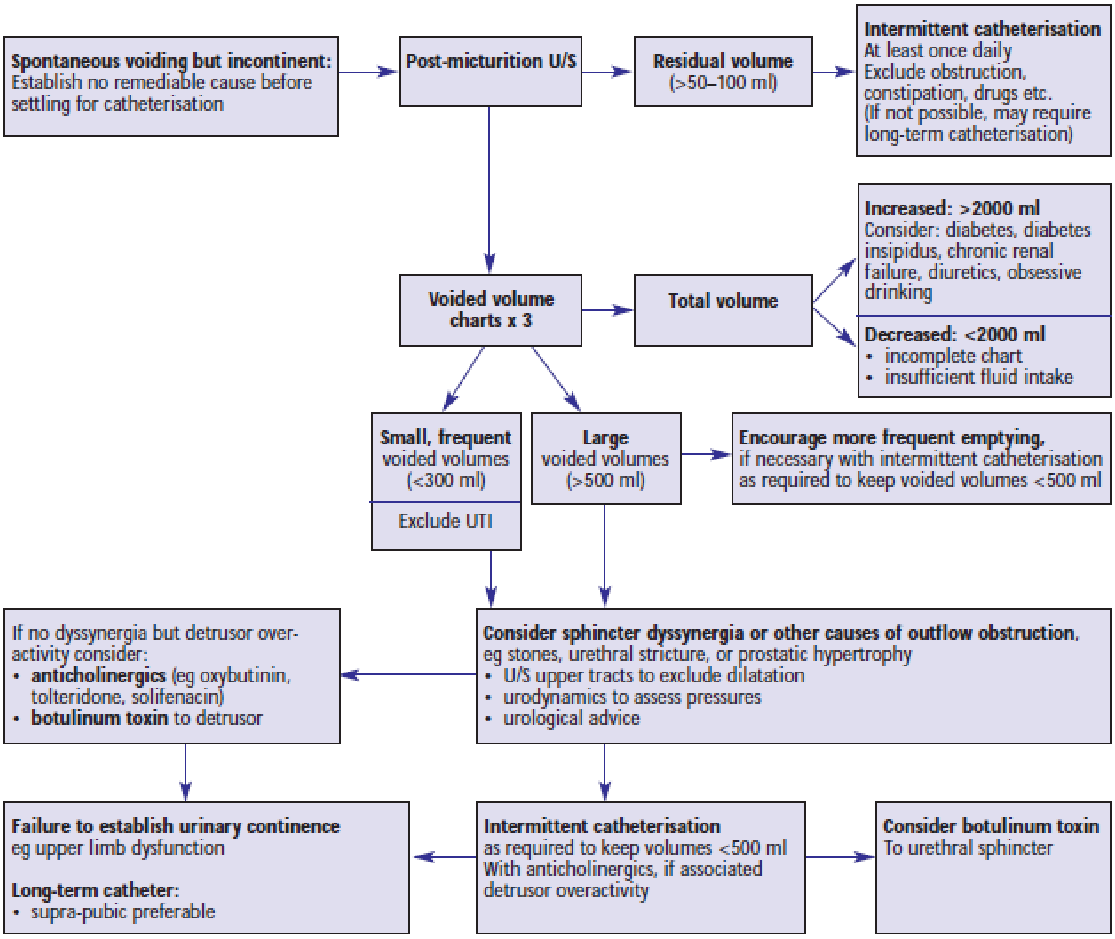

Fig. A2. Managing urinary incontinence in patients with long-term neurological conditions.
U/S = ultrasound; UTI = urinary tract infection.

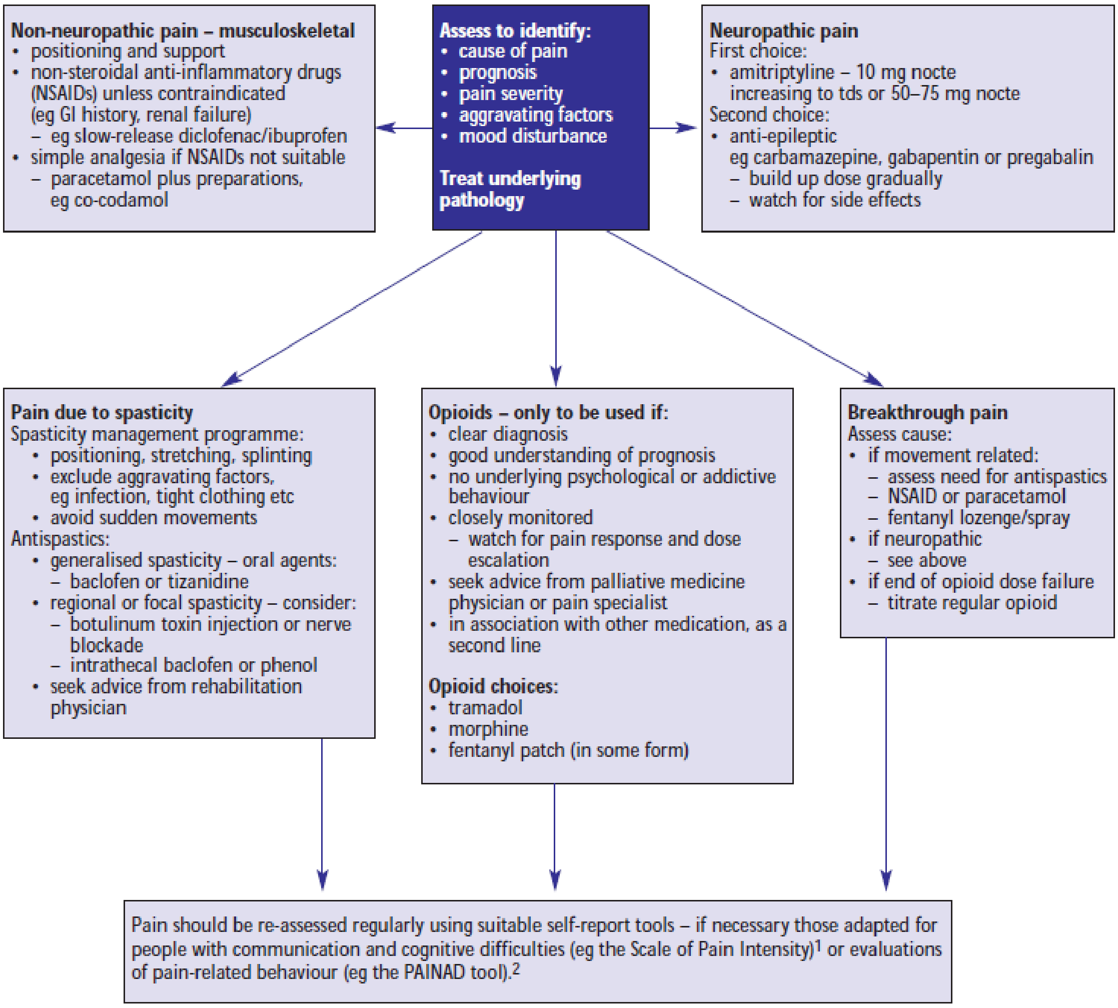

Fig. A3. Managing pain in patients with long-term neurological conditions.

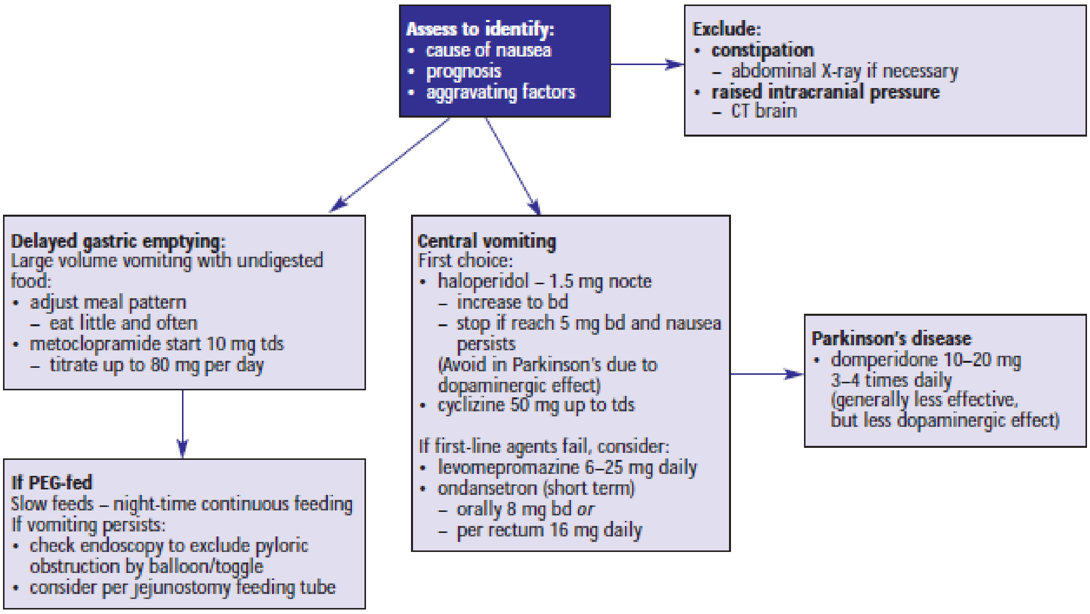

Fig. A4. Managing nausea and vomiting in patients with long-term neurological conditions.
CT = computed tomography; PEG = percutaneous endoscopic gastronomy.

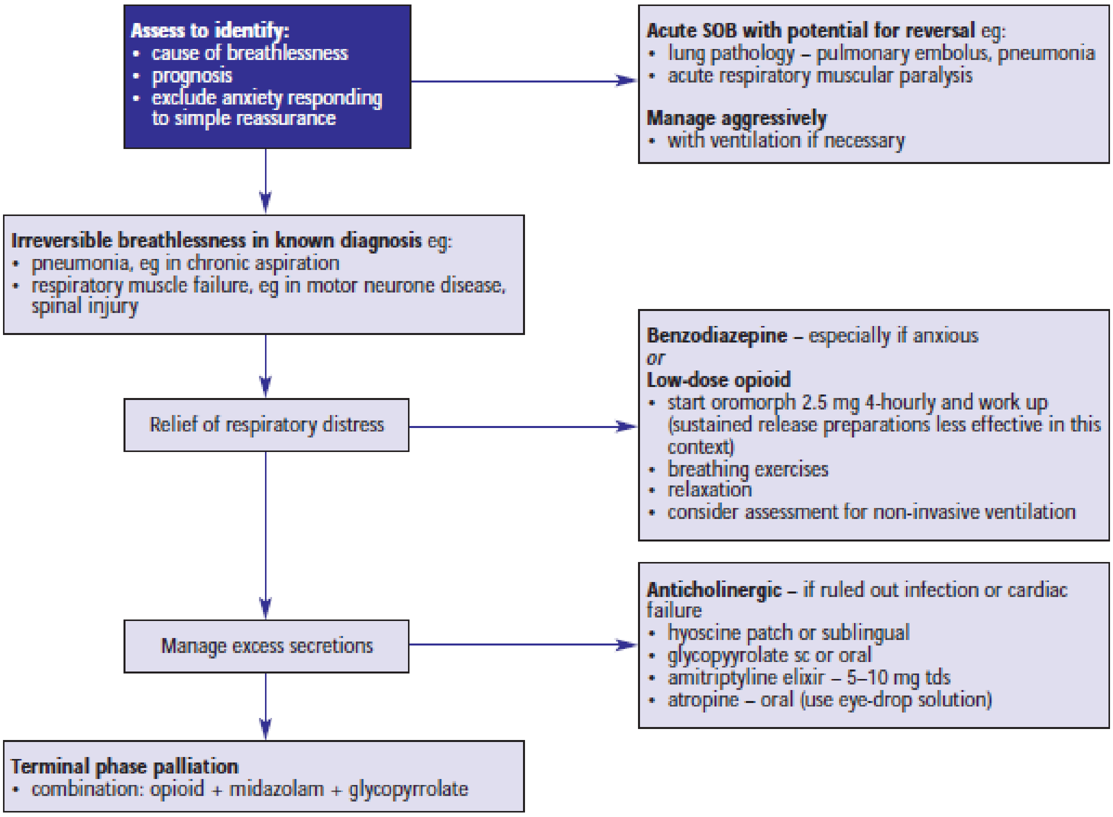

Fig. A5. Managing shortness of breath (SOB) in patients with long-term neurological conditions.

6. Standard Recording of clinical information using the usual recording method for each Multiprofessional Palliative Care Team

A Palliative Care Clinical Record will be documented and maintained for each patient. This will be important to capture and record in a standardised way the delivery of the intervention. The MPCT will record this information using their usual Clinical Record Form, it is clear that each team will have their own paper based or electronic clinical records and we do not wish to duplicate work for busy clinical teams. Having said that, we would require each clinical team to review their usual documentation to ensure it will as a minimum record and report the following.

Phase of illness

Phase	This is the current phase if…	This phase ends when…
**Stable**	Patient's problems and symptoms are adequately controlled by established plan of care **and** further interventions to maintain symptom control and quality of life have been planned **and** family/carer situation is relatively stable and no new issues are apparent.	The needs of the patient and or family/carer increase, requiring changes to the existing plan of care.
**Unstable**	An urgent change in the plan of care or emergency treatment is required **because** the patient experiences a new problem that was not anticipated in the existing plan of care **and/or** the patient experiences a rapid increase in the severity of a current problem **and/or** family's/carer's circumstances change suddenly impacting on patient care.	The new plan of care is in place, it has been reviewed and no further changes to the care plan are required. This does not necessarily mean that the symptom/crisis has fully resolved but there is a clear diagnosis and plan of care (i.e. patient is stable or deteriorating) **and/or** death is likely within days (i.e. patient is now dying).
**Deteriorating**	The care plan is addressing anticipated needs, but requires periodic review, **because** the patient's overall functional status is declining **and** the patient experiences a gradual worsening of existing problem(s) **and/or** the patient experiences a new, but anticipated, problem **and/or** the family/carer experience gradual worsening distress that impacts on the patient care.	Patient condition plateaus (i.e. patient is now stable) **or** an urgent change in the care plan or emergency treatment **and/or** family/ carers experience a sudden change in their situation that impacts on patient care, and urgent intervention is required (i.e. patient is now unstable) **or** death is likely within days (i.e. patient is now dying).
**Dying**	Dying: death is likely within days.	Patient dies **or** patient condition changes and death is no longer likely within days (i.e. patient is now stable or deteriorating).
**Deceased**	The patient has died; bereavement support provided to family/carers is documented in the deceased patient's clinical record.	Case is closed.

Performance status (Australian Modified Karnofsky Scale)

Symptom control and management

Continuity and co-ordination of care, access to services

Psychosocial needs

Information / communication needs

Advance care planning

Assessment of caregiver

Agreed personalised problem list and action plan

IPOS Neuro questionnaires

7. Process data (Appendix 2)

Process evaluations within trials explore the implementation, receipt, and setting of an intervention and help in the interpretation of the outcome results. In this instance, they will be used to study how the intervention is implemented and may provide information on contextual factors that affect the intervention. It will also provide us with information in this multi-site trial about the uniformity of delivery of the intervention, where the "same" intervention may be implemented and received in different ways. Process information is recorded in the MPCT Contact Record Form. The contact record form should be used every time there is a contact with a patient or carer or health professional outside the study visits. This will record the nature and length of the conduct.

8. Quality assurance

As part of the quality assurance procedures, each unit will receive training on delivering the intervention. In addition, random patients will be audited (by the units) and the units will be audited (by members of the trial management group). This will be conducted at random intervals by both site visits and telephone calls. In addition, site visits will be conducted to help to ensure uniformity in delivery of the intervention. The aim is to identify if delivery of the intervention is problematic and find ways to improve this to ensure that:

a) there is early alert of when there are problems with delivery of the intervention which will allow us to resolve this with clinicians and services
b) to improve the extent to which the 'intervention' is offered in a standard way across services, settings, diagnoses and individual practitioners
c) to understand when (b) varies and why
d) to give us data on the clinician's view on compliance of patients with the intervention and the extent to which they received the intervention.

Standard Operating Procedures for care of patients receiving SIPC

1. The MPCT receive the referral from the trial manager with the details of the patient.
2. The MPCT allocate a key worker who telephones the patient referred to introduce themselves, explain about the service, confirm the referral and make first contact. This occurs within 2 working days of receipt of the information. On the telephone call the key worker assesses the severity of problems and determines the urgency required for visit. They arrange the first contact.
3. The first face to face contact occurs within 5 working days of the telephone call (sooner in urgent cases), in the place of a patients' preference. The first contact comprises a specialist palliative care assessment lasting 1-1.5 hours. It includes: assessment of symptom control and management, continuity and coordination of care and access to services, psychosocial needs including responses to loss and change, information needs, in particular, gaps in information, wishes to participate in care, need for advanced care planning. There is assessment of the caregiver when possible. Standard clinical tools are used.
4. As a result of this assessment the key worker generates a problem list with the patient and outlines a proposed action plan, agreed with the patient. This might involve a change in symptom management (e.g. drug change), contact with other services, and/or psychosocial support or counselling during the visit.
5. The team member reviews and revises, at a multiprofessional team meeting, the problem list, their assessment and the action plan to optimise the management of the patient and caregiver and plan future visits. Input from other members of the multiprofessional team occurs at this point.
6. At the same time there is liaison with other relevant clinicians and professionals to arrange, for example, changes in medication (for example, if the patient is at home, changes in medication agreed with the GP).
7. The key worker and other members of the team, if appropriate, implement the plan from the multidisciplinary team meeting.
8. The key worker arranges the next face to face contact with the patient and the caregiver. During the phone contact they make an initial assessment and may make further recommendations. In some instances another relevant member of the team becomes directly involved, as agreed at the multidisciplinary team meeting.
9. The 2nd face to face contact normally occurs within 2 weeks of the first face to face contact.
10. At the 2nd contact, the key worker reviews outcomes from the actions already taken (for example, changes in drugs to control symptoms), reassesses using the same standardised assessment tools, reviews the problem list. If immediate issues are resolved, s/he explores the potential to move to more hidden issues, such as, advanced care planning, future planning or dealing with other information needs. At this visit there is development of a plan for discharge.
11. Following the 2nd contact there is re-discussion of the problem list and action plan at the multidisciplinary team meeting and a repeat of the liaison as appropriate with other relevant professionals.
12. There is contact with the caregiver as required throughout.
13. Following these actions the key worker organises a 3rd visit to the patient normally within 3 to 4 weeks of the 2nd face to face contact – this is determined by patient need and other actions being undertaken.
14. At the 3rd visit there is a review of the outcomes from the actions taken and a reassessment using the standardised assessment tools, a review of the problem list future action plan.
15. Following the 3rd visit there is further discussion with the multiprofessional team and liaison with relevant professionals with a focus on education and plans for future management of problems. Most patients / families will be discharged at this point, for a few 4th or 5th assessments will be needed. It is possible that some patients will be discharged earlier than the 3rd face to face visit, if all the issues which the MPCT is able to resolve have been resolved.
16. In around 10% of cases there is a plan for continuing palliative care. This is based on need and occurs especially if patients are deteriorating or close to dying (estimate is based on phase II trial results(4)).

Need for the intervention

Symptom burden
- Loss of function
- Pain
- Nausea and vomiting
- Feeling sleepy
- Mouth problems
- Constipation
- Poor appetite
- Spasticity
- Fatigue
- Depression
- Bedsores
- Incontinence
- Communication difficulties
- Cognitive problems

Lack of continuity & co-ordination in care

Lack of information about services

End of Life issues

Complex treatment choices

Informal caregiver burden

High health care costs

Repeated hospital admissions with concerns regarding care while in hospital

Short-term Integrated Palliative Care - to support personalised care planning, to empower patients with information and to support in management of symptoms and future decisions regarding care

Palliative Care
- Multi-professional teams
- Trained to deal with complex problems, including pain and symptoms
- Holistic assessment
- Patients and their families/close friends
- *Short-term* - Usually 3 visits, over 6-8 weeks

1.Visit from key worker
Comprehensive palliative care assessment
- Symptom control and management
- Continuity and co-ordination of care, access to services
- Psychosocial needs. Information / communication needs
- Need for advance care planning supported and improved
- Assessment of caregiver
- Agreed personalised problem list and action plan
- Review problem list/plan with multi-professional team & liaison with relevant health, social and voluntary sector professions

2. Visit from key worker or other relevant team member
Review outcomes from actions already taken
Reassessment. If immediate issues resolved, advance care planning. Revise personalised plan and actions. Plan for discharge.
Re-review personalised problem list/plan with multi-professional team & liaison with relevant health professions

3. Visit from key worker or other relevant team member
Review outcomes from actions already taken
Reassessment, revise plan. Reassessment repeated as required.

Discharge. Final Review with multi-professional team & liaison with relevant health professions.

Primary outcome

5 symptoms assessed at 12 weeks
- Pain
- Nausea
- Vomiting
- Sleeping difficulty
- Mouth problems

Secondary outcomes

Hospital admissions

Hospital length of stay

Anxiety & depression

Quality of life

Other symptoms

Palliative needs

Caregiver burden

Caregiver distress

Costs

APPENDIX 1 EXAMPLE PALLIATIVE CARE CLINICAL RECORD FORM (NOTTINGHAM) FOR OPTCARE NEURO

Title<br>Surname<br>Forename<br>Address<br><br><br>Preferred name<br>DOB<br>Age<br>Sex M☐ F☐<br>Marital status S☐ M☐ D☐ Sep☐ W☐<br>☎ Home<br>☎ Mobile	Study number<br><br>NHS number<br><br>Ethnicity<br><br>Religion<br><br>Occupation (current or previous)<br><br>Preferred language English ☐<br>Other

Name<br>Address<br><br><br>Relationship(R)<br>☎ Home (H)<br>Work (W)<br>Mobile (M)	Next of kin

GP<br>Address<br><br><br><br>☎ Fax<br>District Nurse<br>Address<br><br><br>☎ Fax	Consultant (Palliative)<br>Consultant (Neurology)<br>Consultant (Other)<br><br>Community Matron<br>Neurology Specialist Nurse<br><br>Social Worker<br>Other
Diagnosis<br><br><br>Month and year of diagnosis /<br>Relevant medical condition(s)	Home:<br>House ☐ Flat ☐ (floor….warden aided Y☐N☐)<br>Bungalow ☐<br>Toilet: upstairs ☐ downstairs ☐<br>Residential home ☐ Nursing home ☐<br><br>Personal Independence Payment ☐<br>Disability Living Allowance ☐<br>Attendance Allowance ☐<br>DS1500 ☐

Name **Study number**

Family Tree

Family Dynamics

Personal Welfare Lasting Power of Attorney ☐ **Name ……………………………..**

Advance Decision to Refuse Treatment ☐

Any additional preferences or wishes

Patient name: **Study number:** **Date:**

**Summary of History** (*date and underline each episode)*	**Investigations / Results**

MPCT Record
Patient name: **Study number:**

Date & Time	Visits One, Two and Three	Initials
	**Length of visit with patient (also record travel time)**	
	**Phase of illness : Stable, unstable, deteriorating or dying**	
	**Performance status: (as measured by AKPS)**	
	**Symptom control and management**	
	**Continuity and co-ordination of care, access to services**	
	**Psychosocial needs. Information / communication needs**	
	**Advance care planning**	
	**Assessment of caregiver**	
	**Agreed personalised problem list and action plan**	
	**IPOS Neuro completed YES / NO**	
	**Summarise the main points after each visit**	
	**Please also complete the visit and contact recording form for each of the three study visits and all points of contact outside of the three study visits**	

APPENDIX 2

Patient name: ………………………..
Patient DOB: …………………………

MPCT Contact recording form

Please detail all points of contact with the patient/family or carer outside routine visits

***ALL** points of contact with patient/carer outside study visits:*	*Professional type e.g. CNS*	*Contact with whom e.g. patient, carer*	*Date of contact*	*Approximate length of consultation*	*Type of contact (e.g. telephone, home visit or clinical/hospice contact)*	*Administration time related to contact (mins)*	*Brief summary of content and action plan e.g. areas reviewed and plan of care*
1							
2							
3							
4							
	Please continue on a separate sheet if necessary						

Please detail all points of contact you make with other health or social care practitioners regarding the patient/family

Contact	Professional type e.g. GP, Community Matron, Specialist Neurology Nurse	Date of contact	Type of contact (e.g. telephone, face to face, MDM, GSF)	Approximate length of contact (mins)	Administration time related to contact (mins)	Brief summary of content (e.g.referral, update, request medication change, advice, plan of care)
1						
2						
3						
4						
	Please continue on a separate sheet if necessary					

Advance Care Planning

Discussion	Discussion with whom (e.g. patient, carer)	Date of contact	Summary of content			
			Identified preferred place of care- please state	Identified preferred place of	DNACPR? (y/n)	Detail any additional decisions or discussion points e.g. not appropriate to discuss and reasons

				death- please state		
1						
2						
	Please continue on a separate sheet if necessary					

Discharge after 3rd visit

Date of Discharge	***Request ongoing monitoring/ interventions?***	***Continuation on MPCT case load after 12 weeks?***	***Please detail any additional information regarding discharge e.g reasons for continuing on MPCT caseload***

Compliance of patient with intervention

At the end of the 12 week trial period, please rate level of compliance with the intervention by circling one of the following:

Complier	***Partial complier/erratic user***	***Overuser***	***Dropout***

**Receives full intervention as planned**	**Receives some of the intervention but not all, or recommendations not followed**	**Frequent contact with the service**	**Patient dropped out of trial**

APPENDIX 3

MDT PROFORMA

(To be completed after each visit on discussion in MPCT MDT, this is to then be sent to all relevant health professionals and the patient)

BACKGROUND

PHYSICAL

PSYCHOLOGICAL

SOCIAL/FAMILY

SPIRITUAL

INFORMATION NEEDS

PLAN
