# Supplementary material for: Effect of Short-term Integrated Palliative Care on Patient-Reported Outcomes Among Patients Severely Affected With Long-term Neurological Conditions: A Randomized Clinical Trial
Source: JAMA Netw Open. 2020 Aug 28;3(8):e2015061. doi: 10.1001/jamanetworkopen.2020.15061 (PMC7455856; doi:10.1001/jamanetworkopen.2020.15061)
Supplement: Supplement 2. — eTable 1. Outcome measures used in OPTCARE Neuro trial eTable 2. Means and Change Scores of Primary (95% CIs) and Secondary Outcomes (99.55% CIs) at Baseline and 12-Weeks Post Randomisation by Trial Arm, Using Multiply Imputed Data From All Recruited Patients eTable 3. Means and Change Scores of Primary (95% CIs) and Secondary Outcomes (99.55% CIs) at Baseline and 12-Weeks Post Randomisation, by Trial Arm, Using Multiply Imputed Patient Data Excluding Two Ineligible Patients eTable 4. Means and Change Scores of Primary (95% CIs) and Secondary Outcomes (99·55% CI) at Baseline and 12-Weeks Post Randomisation, by Trial Arm, Using Complete Patient Data at Both Baseline and 12-Weeks eTable 5. Means and Change Scores of Primary (95% CIs) and Secondary Outcomes (99·55% CI) at Baseline and 12-Weeks post Randomisation, by Trial Arm, Using Carers With Complete Data at Both Baseline and 12-Weeks eTable 6. Means and Change Scores of Primary (95% CIs) and Secondary Outcomes (99·55% CI) at Baseline and 12-Weeks Post Randomisation, by Trial Arm, Using Patients With Complete Data at Both Baseline and 12-Weeks Plus Imputed Proxy Data if Available at Both Baseline and 12-Weeks eTable 7. Means and Change Scores of Primary (95% CIs) and Secondary Outcomes (99·55% CI) at Baseline and 12-Weeks Post Randomisation by Trial Arm, Using Multiply Imputed Data From Patients With Multiple Sclerosis Only eTable 8. Participant Characteristics by Study Site eTable 9. Unit Costs of Health and Social Care Services in the Analysis eTable 10. Average Care Costs per Person (95% Confidence Intervals) at Baseline and 12 Weeks Post Randomisation, and Changes in Health and Social Care Costs by Trial eTable 11. Patient Demographics for Qualitative Interview Participants eTable 12. Carer Demographics for Qualitative Interview Participants eTable 13. Analytic Framework for the Qualitative Data on the Value and Impact of SIPC eTable 14. Analysis of Fidelity Data [file jamanetwopen-3-e2015061-s002.pdf]

# Supplementary Online Content

Gao W, Wilson R, Hepgul N, et al; the OPTCARE Neuro Trial Investigators. Effect of short-term integrated palliative care on patient-reported outcomes among patients with advanced long-term neurological conditions: a randomized clinical trial. *JAMA Netw Open*. 2020;3(8):e2015061. doi:10.1001/jamanetworkopen.2020.15061


**eTable 1.** Outcome measures used in OPTCARE Neuro trial
**eTable 2.** Means and Change Scores of Primary (95% CIs) and Secondary Outcomes (99.55% CIs) at Baseline and 12-Weeks Post Randomisation by Trial Arm, Using Multiply Imputed Data From All Recruited Patients
**eTable 3.** Means and Change Scores of Primary (95% CIs) and Secondary Outcomes (99.55% CIs) at Baseline and 12-Weeks Post Randomisation, by Trial Arm, Using Multiply Imputed Patient Data Excluding Two Ineligible Patients
**eTable 4.** Means and Change Scores of Primary (95% CIs) and Secondary Outcomes (99·55% CI) at Baseline and 12-Weeks Post Randomisation, by Trial Arm, Using Complete Patient Data at Both Baseline and 12-Weeks
**eTable 5.** Means and Change Scores of Primary (95% CIs) and Secondary Outcomes (99·55% CI) at Baseline and 12-Weeks post Randomisation, by Trial Arm, Using Carers With Complete Data at Both Baseline and 12-Weeks
**eTable 6.** Means and Change Scores of Primary (95% CIs) and Secondary Outcomes (99·55% CI) at Baseline and 12-Weeks Post Randomisation, by Trial Arm, Using Patients With Complete Data at Both Baseline and 12-Weeks Plus Imputed Proxy Data if Available at Both Baseline and 12-Weeks
**eTable 7.** Means and Change Scores of Primary (95% CIs) and Secondary Outcomes (99·55% CI) at Baseline and 12-Weeks Post Randomisation by Trial Arm, Using Multiply Imputed Data From Patients With Multiple Sclerosis Only
**eTable 8.** Participant Characteristics by Study Site
**eTable 9.** Unit Costs of Health and Social Care Services in the Analysis
**eTable 10.** Average Care Costs per Person (95% Confidence Intervals) at Baseline and 12 Weeks Post Randomisation, and Changes in Health and Social Care Costs by Trial
**eTable 11.** Patient Demographics for Qualitative Interview Participants
**eTable 12.** Carer Demographics for Qualitative Interview Participants
**eTable 13.** Analytic Framework for the Qualitative Data on the Value and Impact of SIPC
**eTable 14.** Analysis of Fidelity Data


This supplementary material has been provided by the authors to give readers additional information about their work.

© 2020 Gao W et al. *JAMA Network Open.*

## Outcome measures used in OPTCARE Neuro trial – eTable 1

**eTable 1  Outcome measures used in OPTCARE Neuro trial**

| Measures | Dimension | Details including score range |
|---|---|---|
| IPOS Neuro | Symptoms & Palliative care Outcome | Integrated Palliative Care Outcome Scale (IPOS) for neurological conditions. 42 items (score range: 0 to 168). |
| IPOS Neuro-S8 | Symptoms | IPOS Neuro physical symptom sub-scale. 8 items (score range: 0 to 32). |
| IPOS Neuro-S24 | Symptoms | IPOS Neuro physical symptom subscale. 24 items (score range: 0 to 96). |
| IPOS Neuro-8 | Psychological and spiritual well-being, information needs and practical issues. | IPOS Neuro non-physical subscale. 8 items (score range: 0 to 32). |
| HADS | Psychological distress | Hospital Anxiety and Depression Scale. Measure of anxiety and depression in people with physical health problems. 2 subscales with 7 items each (score range: 0 to 21) |
| EQ-5D | Quality of life & Well-being | EuroQol-5D. Measure of health-related quality of life. Five dimensions plus a visual analogue scale (VAS score range: 0 to 100) |
| FAMCARE-P16 | Patient satisfaction & other aspects | Modified FAMCARE scale P16. Measure of patients' satisfaction with the care they have received in the previous 12-weeks. 16 items (score range: 16 to 80) |
| ZBI-12 | Caregiver burden | 12-item Zarit Burden Inventory. Measure of carer burden. 12 items (score range: 0 to 48) |
| ZBI-positivity | Carer positivity | Zarit Burden Inventory-positivity. Measure of carer positivity. Eight items (score range: 0 to 32) |
| FAMCARE-2 | Carer satisfaction | Modified FAMCARE17-item. Measure of carer satisfaction. 17 items (score range: 17 to 85) |
| CSRI | Healthcare service use & costs | Client Service Receipt Inventory. Service utilization in the previous 12-weeks for use in economic evaluation. Includes use of inpatient, outpatient and home-based services as well as help received from friends and family. |

© 2020 Gao W et al. *JAMA Network Open.*

## Results of sensitivity analyses – eTable 2-7

**eTable 2 Means and change scores of primary (95% CIs) and secondary outcomes (99.55% CIs) at baseline and 12-weeks post randomisation by trial arm, using multiply imputed data from all recruited patients**

| Measure | Time point | SIPC | Standard care | Effect size | p** |
|---|---|---|---|---|---|
| | | N=176 | N=174 | | |
| **Primary outcome** | | | | | |
| **IPOS Neuro-S8, $\bar{x}$ (CI)** | Baseline | 6·89(6·24 to 7·54) | 6·96(6·34 to 7·58) | | |
| | 12-weeks | 6·11(5·46 to 6·77) | 6·68(6·02 to 7·34) | | |
| | Change score | -0·78(-1·29 to -0·26) | -0·28(-0·82 to 0·26) | -0·18(-0·39 to 0·03) | 0·13 |
| **Secondary patient outcomes*** | | | | | |
| **IPOS Neuro-S24, $\bar{x}$ (CI)** | Baseline | 26·69 (24·99 to 28·38) | 27·16 (25·38 to 28·94) | | |
| | 12-weeks | 24·74 (22·92 to 26·55) | 26·27 (24·42 to 28·12) | | |
| | Change score | -1·95 (-3·60 to -0·30) | -0·89 (-2·45 to 0·66) | -0·14(-0·45 to 0·16) | 0·25 |
| **IPOS Neuro 8, $\bar{x}$ (CI)** | Baseline | 11·43 (10·49 to 12·37) | 11·58 (10·55 to 12·62) | | |
| | 12-weeks | 10·59 (9·55 to 11·62) | 11·80 (10·79 to 12·80) | | |
| | Change score | -0·84 (-1·70 to 0·01) | 0·21 (-0·79 to 1·22) | -0·19(-0·50 to 0·11) | 0·07 |
| **IPOS Neuro, $\bar{x}$ (CI)** | Baseline | 47·36 (43·66 to 51·06) | 46·72 (42·78 to 50·65) | | |
| | 12-weeks | 43·14 (37·85 to 48·43) | 44·22 (39·69 to 48·75) | | |
| | Change score | -4·22 (-8·68 to 0·24) | -2·50 (-6·48 to 1·48) | -0·07(-0·39 to 0·24) | 0·56 |
| **HADS anxiety, $\bar{x}$ (CI)** | Baseline | 7·78 (7·09 to 8·46) | 7·51 (6·83 to 8·19) | | |
| | 12-weeks | 7·43 (6·64 to 8·22) | 7·59 (6·86 to 8·33) | | |
| | Change score | -0·35 (-0·88 to 0·19) | 0·08 (-0·42 to 0·59) | -0·11(-0·42 to 0·19) | 0·28 |
| **HADS depression, $\bar{x}$ (CI)** | Baseline | 8·13 (7·55 to 8·71) | 8·31 (7·73 to 8·90) | | |
| | 12-weeks | 7·96 (7·32 to 8·59) | 8·22 (7·62 to 8·82) | | |
| | Change score | -0·17 (-0·60 to 0·25) | -0·09 (-0·56 to 0·38) | -0·05(-0·36 to 0·25) | 0·70 |
| **EQ-5D VAS, $\bar{x}$ (CI)** | Baseline | 52·72 (49·40 to 56·04) | 52·25 (48·64 to 55·86) | | |
| | 12-weeks | 53·69 (49·79 to 57·58) | 50·75 (47·04 to 54·46) | | |
| | Change score | 0·97 (-3·15 to 5·08) | -1·50 (-6·00 to 3·00) | 0·12(-0·18 to 0·43) | 0·31 |
| **SEMCD, $\bar{x}$ (CI)** | Baseline | 5·39 (5·04 to 5·73) | 5·13 (4·78 to 5·48) | | |
| | 12-weeks | 5·28 (4·91 to 5·66) | 4·94 (4·58 to 5·30) | | |
| | Change score | -0·10 (-0·45 to 0·25) | -0·19 (-0·54 to 0·15) | 0·10(-0·21 to 0·40) | 0·38 |
| **FAMCARE P16, $\bar{x}$ (CI)** | Baseline | 50·33 (47·80 to 52·86) | 50·30 (48·08 to 52·52) | | |
| | 12-weeks | 48·08 (45·10 to 51·06) | 47·41 (44·73 to 50·10) | | |
| | Change score | -2·26 (-4·87 to 0·36) | -2·89 (-5·19 to -0·59) | 0·04(-0·27 to 0·34) | 0·78 |
| **Secondary carer outcomes*** | | | | | |
| **ZBI 12, $\bar{x}$ (CI)** | Baseline | 18·25 (16·43 to 20·06) | 18·68 (17·03 to 20·32) | | |
| | 12-weeks | 18·60 (16·77 to 20·43) | 18·92 (17·11 to 20·72) | | |
| | Change score | 0·35 (-0·56 to 1·26) | 0·24 (-0·72 to 1·20) | 0·02(-0·28 to 0·33) | 0·87 |
| **ZBI Positivity, $\bar{x}$ (CI)** | Baseline | 18·97 (17·87 to 20·08) | 18·72 (17·57 to 19·86) | | |
| | 12-weeks | 18·87 (17·64 to 20·10) | 18·12 (16·77 to 19·47) | | |
| | Change score | -0·10 (-1·01 to 0·81) | -0·59 (-1·54 to 0·35) | 0·10(-0·21 to 0·41) | 0·38 |
| **FAMCARE 2, $\bar{x}$ (CI)** | Baseline | 53·81 (50·95 to 56·67) | 53·98 (51·21 to 56·74) | | |
| | 12-weeks | 53·99 (50·52 to 57·46) | 53·23 (49·92 to 56·53) | | |
| | Change score | 0·19 (-3·25 to 3·63) | -0·75 (-3·43 to 1·92) | 0·04(-0·27 to 0·34) | 0·67 |

* 99·55% confidence intervals, Bonferroni correction to control for multiple testing (adjusted alpha=0·0045,0·05/11).
**P value for two group comparisons using generalised linear mixed model, adjusting for baseline score and ethnicity with centre modelled as a random effect.

© 2020 Gao W et al. *JAMA Network Open.*

**eTable 3: Means and change scores of primary (95% CIs) and secondary outcomes (99.55% CIs) at baseline and 12-weeks post randomisation, by trial arm, using multiply imputed patient data excluding two ineligible patients**

| Measure | Time point | SIPC | Standard care | Effect size | P** |
|---|---|---|---|---|---|
| | | N=175 | N=173 | | |
| **Primary outcome** | | | | | |
| **IPOS Neuro-S8** $\bar{x}$ **(CI)** | Baseline | 6·83(6·16 to 7·50) | 7·02(6·39 to 7·65) | | |
| | 12-weeks | 6·07(5·42 to 6·71) | 6·76(6·07 to 7·45) | | |
| | Change score | -0·77(-1·37 to -0·16) | -0·26(-0·81 to 0·29) | -0·16(-0·37 to 0·05) | 0·12 |
| **Secondary patient outcomes*** | | | | | |
| **IPOS Neuro-S24** $\bar{x}$ **(CI)** | Baseline | 26·61 (24·90 to 28·32) | 27·17 (25·48 to 28·86) | | |
| | 12-weeks | 24·64 (23·00 to 26·28) | 26·27 (24·38 to 28·17) | | |
| | Change score | -1·97 (-3·31 to -0·63) | -0·90 (-2·46 to 0·66) | -0·13(-0·44 to 0·18) | 0·25 |
| **IPOS Neuro 8** $\bar{x}$ **(CI)** | Baseline | 11·52 (10·55 to 12·49) | 11·44 (10·43 to 12·45) | | |
| | 12-weeks | 10·72 (9·62 to 11·83) | 11·65 (10·68 to 12·61) | | |
| | Change score | -0·80 (-1·72 to 0·12) | 0·21 (-0·66 to 1·08) | -0·18(-0·49 to 0·12) | 0·11 |
| **IPOS Neuro** $\bar{x}$ **(CI)** | Baseline | 47·47 (43·09 to 51·86) | 47·55 (43·62 to 51·48) | | |
| | 12-weeks | 42·53 (38·25 to 46·81) | 44·07 (39·70 to 48·44) | | |
| | Change score | -4·94 (-9·39 to -0·49) | -3·48 (-7·95 to 0·99) | -0·06(-0·38 to 0·25) | 0·59 |
| **HADS anxiety** $\bar{x}$ **(CI)** | Baseline | 7·81 (7·11 to 8·50) | 7·61 (6·93 to 8·30) | | |
| | 12-weeks | 7·50 (6·70 to 8·30) | 7·75 (7·03 to 8·46) | | |
| | Change score | -0·31 (-0·82 to 0·21) | 0·13 (-0·37 to 0·64) | -0·13(-0·43 to 0·18) | 0·25 |
| **HADS depression** $\bar{x}$ **(CI)** | Baseline | 8·14 (7·55 to 8·72) | 8·33 (7·76 to 8·89) | | |
| | 12-weeks | 7·91 (7·24 to 8·58) | 8·23 (7·60 to 8·86) | | |
| | Change score | -0·23 (-0·71 to 0·25) | -0·09 (-0·58 to 0·39) | -0·06(-0·37 to 0·25) | 0·57 |
| **EQ-5D VAS** $\bar{x}$ **(CI)** | Baseline | 53·14 (49·73 to 56·55) | 52·37 (48·76 to 55·98) | | |
| | 12-weeks | 53·52 (49·57 to 57·46) | 50·42 (46·56 to 54·28) | | |
| | Change score | 0·37 (-4·17 to 4·92) | -1·95 (-6·11 to 2·20) | 0·12(-0·19 to 0·42) | 0·33 |
| **SEMCD** $\bar{x}$ **(CI)** | Baseline | 5·37 (5·01 to 5·72) | 5·14 (4·77 to 5·50) | | |
| | 12-weeks | 5·31 (4·95 to 5·66) | 4·95 (4·57 to 5·32) | | |
| | Change score | -0·06 (-0·41 to 0·29) | -0·19 (-0·55 to 0·17) | 0·11(-0·20 to 0·41) | 0·35 |
| **FAMCARE P16** $\bar{x}$ **(CI)** | Baseline | 50·20 (47·74 to 52·67) | 50·18 (47·78 to 52·59) | | |
| | 12-weeks | 48·00 (45·14 to 50·85) | 47·45 (44·57 to 50·33) | | |
| | Change score | -2·21 (-4·74 to 0·32) | -2·73 (-5·19 to -0·27) | 0·03(-0·27 to 0·34) | 0·83 |
| **Secondary carer outcomes*** | | | | | |
| **ZBI 12** $\bar{x}$ **(CI)** | Baseline | 18·30 (16·69 to 19·90) | 18·66 (16·83 to 20·49) | | |
| | 12-weeks | 18·58 (16·77 to 20·40) | 18·91 (16·96 to 20·86) | | |
| | Change score | 0·28 (-0·60 to 1·17) | 0·25 (-0·67 to 1·16) | 0·00(-0·30 to 0·31) | 0·95 |
| **ZBI Positivity** $\bar{x}$ **(CI)** | Baseline | 18·98 (17·85 to 20·11) | 18·77 (17·50 to 20·05) | | |
| | 12-weeks | 18·83 (17·59 to 20·08) | 18·13 (16·85 to 19·41) | | |
| | Change score | -0·15 (-1·01 to 0·71) | -0·64 (-1·49 to 0·20) | 0·10(-0·21 to 0·40) | 0·34 |
| **FAMCARE 2** $\bar{x}$ **(CI)** | Baseline | 54·05 (51·32 to 56·78) | 53·53 (50·83 to 56·23) | | |
| | 12-weeks | 53·95 (50·68 to 57·23) | 52·68 (49·01 to 56·36) | | |
| | Change score | -0·09 (-3·18 to 2·99) | -0·84 (-3·94 to 2·25) | 0·05(-0·25 to 0·36) | 0·62 |

* 99·55% confidence intervals, Bonferroni correction to control for multiple testing (adjusted alpha=0·0045,0·05/11).
**P value for two group comparisons using generalised linear mixed model, adjusting for baseline score with centre modelled as a random effect

© 2020 Gao W et al. *JAMA Network Open.*

**eTable 4: Means and change scores of primary (95% CIs) and secondary outcomes (99·55% CI) at baseline and 12-weeks post randomisation, by trial arm, using complete patient data at both baseline and 12-weeks**

| Measure | Time point | SIPC | Standard care | ES | p** |
|---|---|---|---|---|---|
| **Primary outcome** | | | | | |
| **IPOS Neuro-S8** | Baseline | 6·68 (6·04 to 7·33) | 7·01 (6·34 to 7·69) | | |
| $\bar{x}$ (CI) | 12-weeks | 5·90 (5·28 to 6·52) | 6·72 (6·01 to 7·42) | | |
| **N=270** | Change score | -0·78 (-1·30 to -0·27) | -0·30 (-0·84 to 0·24) | -0·16 (-0·39 to 0·08) | 0·09 |
| **Secondary patient outcomes*** | | | | | |
| **IPOS Neuro-S24** | Baseline | 25·57 (22·94 to 28·20) | 27·57 (24·80 to 30·35) | | |
| $\bar{x}$ (CI) | 12-weeks | 23·22 (20·70 to 25·74) | 26·73 (23·75 to 29·72) | | |
| **N=235** | Change score | -2·35 (-4·00 to -0·70) | -0·84 (-3·28 to 1·60) | -0·19 (-0·56 to 0·17) | 0·032 |
| **IPOS Neuro 8** | Baseline | 10·78 (9·36 to 12·20) | 11·34 (9·66 to 13·01) | | |
| $\bar{x}$ (CI) | 12-weeks | 9·56 (8·11 to 11·01) | 11·74 (10·17 to 13·31) | | |
| **N=246** | Change score | -1·22 (-2·47 to 0·03) | 0·41 (-0·95 to 1·77) | -0·32 (-0·68 to 0·03) | 0·001 |
| **IPOS Neuro** | Baseline | 42·22 (34·53 to 49·92) | 51·58 (42·70 to 60·46) | | |
| $\bar{x}$ (CI) | 12-weeks | 37·86 (28·88 to 46·85) | 46·86 (37·31 to 56·41) | | |
| **N=79** | Change score | -4·36 (-8·45 to -0·27) | -4·72 (-11·21 to 1·76) | 0·03 (-0·60 to 0·66) | 0·80 |
| **HADS anxiety** | Baseline | 7·39 (6·35 to 8·44) | 7·51 (6·49 to 8·56) | | |
| $\bar{x}$ (CI) | 12-weeks | 6·95 (5·82 to 8·09) | 7·67 (6·64 to 8·70) | | |
| **N=275** | Change score | -0·44 (-1·18 to 0·30) | 0·16 (-0·56 to 0·88) | -0·20 (-0·54 to 0·13) | 0·07 |
| **HADS depression** | Baseline | 7·89 (6·99to 8·79) | 8·35 (7·49 to 9·21) | | |
| $\bar{x}$ (CI) | 12-weeks | 7·62 (6·65 to 8·60) | 8·27 (7·42 to 9·13) | | |
| **N=275** | Change score | -0·27 (-0·92 to 0·39) | -0·08 (-0·78 to 0·63) | -0·07 (-0·40 to 0·27) | 0·33 |
| **EQ-5D VAS** | Baseline | 54·13 (49·03 to 59·23) | 52·18 (46·68 to 57·68) | | |
| $\bar{x}$ (CI) | 12-weeks | 54·72 (49·34 to 60·10) | 50·20 (44·99 to 55·41) | | |
| **N=281** | Change score | 0·59 (-5·63 to 6·81) | -1·98 (-8·15 to 4·20) | 0·10 (-0·23 to 0·43) | 0·11 |
| **SEMCD** | Baseline | 5·56 (5·03 to 6·10) | 5·12 (4·55 to 5·69) | | |
| $\bar{x}$ (CI) | 12-weeks | 5·50 (4·99 to 6·01) | 4·93 (4·39 to 5·47) | | |
| **N=274** | Change score | -0·06 (-0·54 to 0·42) | -0·19 (-0·69 to 0·31) | 0·06 (-0·27 to 0·40) | 0·12 |
| **FAMCARE P16** | Baseline | 54·05 (50·01 to 58·09) | 52·18 (48·40 to 55·95) | | |
| $\bar{x}$ (CI) | 12-weeks | 54·82 (50·70 to 58·95) | 50·77 (46·66 to 54·88) | | |
| **N=193** | Change score | 0·77 (-3·66 to 5·20) | -1·40 (-5·05 to 2·25) | 0·16 (-0·24 to 0·56) | 0·08 |

* 99·55% confidence intervals, Bonferroni correction to control for multiple testing (adjusted alpha=0·0045,0·05/11)·
**P value for two group comparisons using generalised linear mixed model, adjusting for baseline score with centre modelled as a random effect

© 2020 Gao W et al. *JAMA Network Open.*

**eTable 5: Means and change scores of primary (95% CIs) and secondary outcomes (99·55% CI) at baseline and 12-weeks post randomisation, by trial arm, using carers with complete data at both baseline and 12-weeks**

| Measure | Time point | SIPC | Standard care | ES | p** |
|---|---|---|---|---|---|
| **Primary outcome** | | | | | |
| **IPOS Neuro-S8** | Baseline | 7·37 (6·58 to 8·15) | 7·01 (6·22 to 7·80) | | |
| $\bar{x}$ (CI) | 12-weeks | 6·14 (5·29 to 7·00) | 6·26 (5·45 to 7·07) | | |
| **N=175** | Change score | -1·22 (-1·89 to -0·55) | -0·75 (-1·39 to -0·11) | -0·15 (-0·45 to 0·15) | 0·40 |
| **Secondary patient outcomes*** | | | | | |
| **IPOS Neuro-S24** | Baseline | 30·29 (27·18 to 33·40) | 31·75 (28·16 to 35·33) | | |
| $\bar{x}$ (CI) | 12-weeks | 26·30 (22·30 to 30·30) | 28·73 (25·16 to 32·31) | | |
| **N=146** | Change score | -3·99 (-6·80 to -1·18) | -3·01 (-5·85 to -0·18) | -0·12 (-0·58 to 0·34) | 0·34 |
| **IPOS Neuro 8** | Baseline | 12·88 (11·26 to 14·50) | 12·61 (11·00 to 14·22) | | |
| $\bar{x}$ (CI) | 12-weeks | 10·73 (9·01 to 12·44) | 12·11 (10·32 to 13·91) | | |
| **N=178** | Change score | -2·15 (-3·51 to -0·80) | -0·49 (-1·86 to 0·87) | -0·38 (-0·80 to 0·04) | 0·011 |
| **IPOS Neuro** | Baseline | 55·98 (47·06 to 64·89) | 54·06 (44·19 to 63·94) | | |
| $\bar{x}$ (CI) | 12-weeks | 49·34 (39·70 to 58·98) | 48·09 (38·12 to 58·07) | | |
| **N=73** | Change score | -6·63 (-12·63 to -0·64) | -5·97 (-10·99 to -0·95) | -0·06 (-0·71 to 0·60) | 0·87 |
| **Secondary carer outcomes*** | | | | | |
| **ZBI 12** | Baseline | 18·62 (15·87 to 21·38) | 19·29 (16·56 to 22·03) | | |
| $\bar{x}$ (CI) | 12-weeks | 19·11 (16·15 to 22·08) | 19·52 (16·67 to 22·36) | | |
| **N=193** | Change score | 0·49 (-0·87 to 1·85) | 0·22 (-1·33 to 1·77) | 0·05 (-0·35 to 0·45) | 0·76 |
| **ZBI Positivity** | Baseline | 19·34 (17·46 to 21·22) | 18·70 (16·76 to 20·64) | | |
| $\bar{x}$ (CI) | 12-weeks | 19·37 (17·34 to 21·40) | 17·83 (15·94 to 19·72) | | |
| **N=193** | Change score | 0·03 (-1·43 to 1·49) | -0·87 (-2·32 to 0·58) | 0·18 (-0·22 to 0·58) | 0·11 |
| **FAMCARE 2** | Baseline | 60·09 (55·81 to 64·37) | 56·22 (52·06 to 60·37) | | |
| $\bar{x}$ (CI) | 12-weeks | 60·21 (55·97 to 64·45) | 56·62 (52·22 to 61·01) | | |
| **N=140** | Change score | 0·12 (-4·50 to 4·74) | 0·40 (-3·70 to 4·50) | -0·02 (-0·49 to 0·45) | 0·34 |

* 99·55% confidence intervals, Bonferroni correction to control for multiple testing (adjusted alpha=0·0045,0·05/11).
**P value for two group comparisons using generalised linear mixed model, adjusting for baseline score with centre modelled as a random effect.

© 2020 Gao W et al. *JAMA Network Open.*

**eTable 6: Means and change scores of primary (95% CIs) and secondary outcomes (99·55% CI) at baseline and 12-weeks post randomisation, by trial arm, using patients with complete data at both baseline and 12-weeks plus imputed proxy data if available at both baseline and 12-weeks**

| Measure | Time point | All | SIPC | Standard care | p** |
|---|---|---|---|---|---|
| **Primary outcome** | | | | | |
| **IPOS Neuro-S8** $\bar{x}$ (CI) **N=308** | Baseline | 6·81 (6·38 to 7·25) | 6·79 (6·18 to 7·40) | 6·83 (6·20 to 7·46) | |
| | 12 weeks | 6·17 (5·74 to 6·61) | 5·84 (5·26 to 6·43) | 6·50 (5·85 to 7·15) | |
| | Change score | -0·64 (-0·99 to -0·29) | -0·95 (-1·44 to -0·46) | -0·33 (-0·83 to 0·17) | 0·05 |
| **Secondary patient outcomes*** | | | | | |
| **IPOS Neuro-S24** $\bar{x}$ (CI) **N=278** | Baseline | 27·74 (25·97 to 29·51) | 27·11 (24·57 to 29·64) | 28·37 (25·85 to 30·89) | |
| | 12 weeks | 25·85 (24·03 to 27·68) | 24·53 (22·02 to 27·04) | 27·17 (24·50 to 29·84) | |
| | Change score | -1·88 (-3·22 to -0·55) | -2·58 (-4·14 to -1·01) | -1·19 (-3·39 to 1·00) | 0·050 |
| **IPOS Neuro S42** $\bar{x}$ (CI) **N=120** | Baseline | 51·19 (46·28 to 56·10) | 51·11 (43·86 to 58·37) | 51·27 (44·32 to 58·22) | |
| | 12 weeks | 46·66 (41·37 to 51·95) | 46·33 (38·51 to 54·15) | 47·00 (39·51 to 54·49) | |
| | Change score | -4·53 (-7·67 to -1·40) | -4·79 (-8·85 to -0·72) | -4·27 (-9·26 to 0·72) | 0·80 |
| **IPOS Neuro 8** $\bar{x}$ (CI) **N=290** | Baseline | 11·50 (10·50 to 12·51) | 11·51 (10·15 to 12·87) | 11·50 (9·99 to 13·00) | |
| | 12 weeks | 10·91 (9·91 to 11·91) | 10·12 (8·71 to 11·52) | 11·68 (10·27 to 13·09) | |
| | Change score | -0·59 (-1·44 to 0·26) | -1·39 (-2·57 to -0·21) | 0·18 (-1·03 to 1·40) | 0·003 |

*99·55% confidence intervals, Bonferroni correction to control for multiple testing (adjusted alpha=0·0045,0·05/11).
**P value for two group comparisons using generalised linear mixed model, adjusting for baseline score with centre modelled as a random effect·

© 2020 Gao W et al. *JAMA Network Open.*

**eTable 7 Means and change scores of primary (95% CIs) and secondary outcomes (99·55% CI) at baseline and 12-weeks post randomisation by trial arm, using multiply imputed data from patients with multiple sclerosis only**

| Measure | Time point | SIPC | Standard care | ES | *P*** |
|---|---|---|---|---|---|
| | | N=74 | N=74 | | |
| **Primary outcome** | | | | | |
| **IPOS Neuro-S8, $\bar{x}$ (CI)** | Baseline | 7·08(6·07 to 8·09) | 7·10(6·24 to 7·96) | | |
| | 12-weeks | 6·25(5·19 to 7·31) | 6·35(5·32 to 7·38) | | |
| | Change score | -0·83(-1·66 to 0·00) | -0·75(-1·49 to 0·00) | -0·03(-0·35 to 0·30) | 0·88 |
| **Secondary patient outcomes*** | | | | | |
| **IPOS Neuro-S24, $\bar{x}$ (CI)** | Baseline | 27·61(23·51 to 31·71) | 27·74(24·18 to 31·31) | | |
| | 12-weeks | 24·70(20·63 to 28·76) | 24·98(20·53 to 29·43) | | |
| | Change score | -2·91(-6·41 to 0·59) | -2·76(-6·63 to 1·11) | -0·02(-0·49 to 0·45) | 0·90 |
| **IPOS Neuro 8, $\bar{x}$ (CI)** | Baseline | 10·75(8·25 to 13·26) | 11·38(8·97 to 13·80) | | |
| | 12-weeks | 9·69(7·32 to 12·06) | 10·91(8·54 to 13·29) | | |
| | Change score | -1·06(-3·20 to 1·07) | -0·47(-2·35 to 1·41) | -0·15(-0·62 to 0·32) | 0·37 |
| **IPOS Neuro, $\bar{x}$ (CI)** | Baseline | 48·13(36·51 to 59·75) | 46·90(36·76 to 57·04) | | |
| | 12-weeks | 43·56(31·56 to 55·56) | 43·83(34·14 to 53·51) | | |
| | Change score | -4·58(-14·59 to 5·44) | -3·07(-14·09 to 7·95) | -0·04(-0·52 to 0·43) | 0·79 |
| **HADS anxiety, $\bar{x}$ (CI)** | Baseline | 7·48(5·88 to 9·07) | 7·04(5·43 to 8·66) | | |
| | 12-weeks | 6·78(5·07 to 8·49) | 6·98(5·41 to 8·54) | | |
| | Change score | -0·70(-1·79 to 0·40) | -0·07(-1·15 to 1·02) | -0·19(-0·66 to 0·28) | 0·25 |
| **HADS depression, $\bar{x}$ (CI)** | Baseline | 8·38(6·95 to 9·81) | 8·01(6·70 to 9·32) | | |
| | 12-weeks | 7·77(6·27 to 9·26) | 8·01(6·74 to 9·29) | | |
| | Change score | -0·61(-1·51 to 0·29) | 0·00(-1·17 to 1·17) | -0·18(-0·65 to 0·29) | 0·28 |
| **EQ-5D VAS, $\bar{x}$ (CI)** | Baseline | 53·32(45·42 to 61·23) | 54·68(46·44 to 62·92) | | |
| | 12-weeks | 55·25(45·87 to 64·63) | 50·36(41·04 to 59·69) | | |
| | Change score | 1·93(-8·58 to 12·44) | -4·32(-14·54 to 5·91) | 0·21(-0·26 to 0·68) | 0·21 |
| **SEMCD, $\bar{x}$ (CI)** | Baseline | 5·50(4·65 to 6·34) | 5·58(4·79 to 6·37) | | |
| | 12-weeks | 5·65(4·82 to 6·49) | 5·42(4·61 to 6·23) | | |
| | Change score | 0·16(-0·55 to 0·86) | -0·16(-0·89 to 0·57) | 0·14(-0·32 to 0·61) | 0·38 |
| **FAMCARE P16, $\bar{x}$ (CI)** | Baseline | 45·59(39·43 to 51·75) | 46·27(41·05 to 51·50) | | |
| | 12-weeks | 45·82(39·30 to 52·34) | 44·11(37·83 to 50·39) | | |
| | Change score | 0·23(-5·27 to 5·73) | -2·16(-7·43 to 3·11) | 0·15(-0·32 to 0·61) | 0·37 |
| **Secondary carer outcomes*** | | | | | |
| **ZBI 12, $\bar{x}$ (CI)** | Baseline | 18·47(14·09 to 22·86) | 18·69(14·95 to 22·44) | | |
| | 12-weeks | 18·62(13·94 to 23·30) | 18·28(14·24 to 22·32) | | |
| | Change score | 0·15(-1·71 to 2·00) | -0·42(-2·75 to 1·91) | 0·09(-0·39 to 0·57) | 0·58 |
| **ZBI Positivity, $\bar{x}$ (CI)** | Baseline | 19·26(16·49 to 22·04) | 18·77(16·18 to 21·36) | | |
| | 12-weeks | 18·37(15·37 to 21·38) | 17·52(14·85 to 20·19) | | |
| | Change score | -0·89(-2·66 to 0·87) | -1·25(-3·58 to 1·08) | 0·08(-0·39 to 0·56) | 0·61 |
| **FAMCARE 2, $\bar{x}$ (CI)** | Baseline | 52·94(46·34 to 59·54) | 52·83(46·40 to 59·26) | | |
| | 12-weeks | 48·98(41·22 to 56·75) | 49·45(40·61 to 58·30) | | |
| | Change score | -3·96(-11·06 to 3·14) | -3·38(-11·50 to 4·74) | -0·03(-0·50 to 0·45) | 0·87 |

* 99·55% confidence intervals, Bonferroni correction to control for multiple testing (adjusted alpha=0·0045,0·05/11).
**P value for two group comparisons using generalised linear mixed model, adjusting for baseline score with centre modelled as a random effect

© 2020 Gao W et al. *JAMA Network Open.*

**Participant characteristics by study site – eTable 8**

**eTable 8 Participant characteristics by study site**

| Value | Center 1 | Center 2 | Center 3 | Center 4 | Center 5 | Center 6 | Center 7 | P value*** |
|---|---|---|---|---|---|---|---|---|
| N | 37 | 50 | 36 | 100 | 76 | 3 | 48 | - |
| Age | 73.8(10.5) | 64.4(11.3) | 68.8(11.9) | 65.6(13.2) | 64.4(10.3) | 73.7(7.5) | 68.5(10.0) | 0.0006 |
| Male (%) | 18(48.6) | 25(50.0) | 20(55.6) | 52(52.0) | 44(57.9) | 1(33.3) | 19(39.6) | 0.50 |
| With capacity (%) | 32(86.5) | 47(94.0) | 28(77.8) | 83(83.0) | 70(92.1) | 3(100.0) | 48(100.0) | 0.0023 |
| Diagnosis* | | | | | | | | <0.0001 |
| MS | 8(21.6) | 24(48.0) | 6(16.7) | 59(59.0) | 30(39.5) | 3(100.0) | 18(37.5) | |
| IPD | 28(75.7) | 22(44.0) | 24(66.7) | 30(30.0) | 20(26.3) | 0(0.0) | 16(33.3) | |
| MSA | 0(0.0) | 2(4.0) | 2(5.6) | 5(5.0) | 2(2.6) | 0(0.0) | 1(2.1) | |
| PSP | 0(0.0) | 2(4.0) | 2(5.6) | 5(5.0) | 11(14.5) | 0(0.0) | 5(10.4) | |
| MND | 0(0.0) | 0(0.0) | 2(5.6) | 0(0.0) | 13(17.1) | 0(0.0) | 8(16.7) | |
| Other | 1(2.7) | 0(0.0) | 0(0.0) | 1(1.0) | 0(0.0) | 0(0.0) | 0(0.0) | |
| Australia-modified Karnofsky performance scale** | | | | | | | | <0.0001 |
| 20 | 0(0.0) | 0(0.0) | 2(5.6) | 4(4.1) | 5(6.7) | 0(0.0) | 1(2.1) | |
| 30 | 0(0.0) | 0(0.0) | 2(5.6) | 2(2.0) | 6(8.0) | 0(0.0) | 0(0.0) | |
| 40 | 0(0.0) | 1(2.0) | 4(11.1) | 21(21.4) | 4(5.3) | 1(33.3) | 1(2.1) | |
| 50 | 12(32.4) | 22(44.0) | 16(44.4) | 41(41.8) | 45(60.0) | 1(33.3) | 33(68.8) | |
| 60 | 21(56.8) | 23(46.0) | 11(30.6) | 24(24.5) | 14(18.7) | 1(33.3) | 8(16.7) | |
| 70 | 4(10.8) | 4(8.0) | 0(0.0) | 4(4.1) | 1(1.3) | 0(0.0) | 4(8.3) | |
| 80 | 0(0.0) | 0(0.0) | 1(2.8) | 1(1.0) | 0(0.0) | 0(0.0) | 1(2.1) | |
| 90 | 0(0.0) | 0(0.0) | 0(0.0) | 1(1.0) | 0(0.0) | 0(0.0) | 0(0.0) | |

*MS: Multiple sclerosis; IPD: Idiopathic Parkinson's Disease; MSA: Multiple system atrophy; PSP: Progressive supranuclear palsy; MND: Motor Neurone Disease. **20: Totally bedfast and requiring extensive nursing care by professionals and/or family; 30: Almost completely bedfast; 40: In bed more than 50% of the time; 50: Considerable assistance and frequent medical care required; 60: Able to care for most needs; but requires occasional assistance; 70: Cares for self; unable to carry on normal activity or to do active work; 80: Normal activity with effort; some signs or symptoms of disease; 90: Able to carry on normal activity; minor sign of symptoms of disease; 100: Normal; no complaints; no evidence of disease. ***: all comparisons excluded center 6. Age was compared using ANOVA, other variables were compared using Chi square test

© 2020 Gao W et al. *JAMA Network Open.*

## Results of health economic analysis – eTable 9-10

### eTable 9. Unit costs* of health and social care services in the analysis

| Service item | Unit | Unit cost (£) | Note |
|---|---|---|---|
| Intensive care unit | Overnight stay | 329.538 | |
| Neurological ward | | 299/444 | |
| Medical ward | | 3058.14, 298.42 daily >6 | |
| Specialist rehabilitation ward | | 378.25 | |
| Care home | | 104.43 | |
| Respite care | Per diem | 1059, 2327, 1847 | |
| Day hospital | | 355 | |
| NHS day care | | 59 | |
| Palliative day care | | 194.8 | |
| Rehabilitation day unit | | | |
| Neurology day care | | | |
| Social services day centre | | 72 | |
| Voluntary organisation day/resource centre | | 194.8 | |
| Support groups or societies | | 72 | |
| A&E | Per visit | 137.74 | |
| Ambulance | | 96.25 | |
| GP at GP surgery | Per consultation | 28 | |
| Neurologist | | 58 | |
| Palliative care doctor/consultant | | 59 | |
| General practice nurse | Per hour | 47 | prorated |
| Community mental health nurse | | 67 | |
| Palliative care nurse | | 65 | |
| Specialist Parkinson's nurse | Per visit | 22 | |
| Specialist MS nurse | | | |
| Specialist MND nurse | | | |
| Physiotherapist | Per session | 34 | |
| Occupational therapist | | 44 | |
| Speech therapist | | 34 | |
| Social worker | | 57 | |
| Psychologist | | 32 | |
| Counsellor | | 32 | |
| Priest/Clergy/Chaplain/Imam/Rabbi | | 39 | |
| Mental health worker | | 22 | |
| Dentist | | 102 | |
| Dietician | | 17 | |

*Sources:

1. NHS reference costs 2015 to 2016. 2016 [Available from: https://www.gov.uk/government/publications/nhs-reference-costs-2015-to-2016.
2. Curtis L, Burns A. Unit Costs of Health and Social Care 2016. In: Unit PSSR, editor. Canterbury: University of Kent; 2016.

© 2020 Gao W et al. *JAMA Network Open.*

**eTable 10: Average care costs per person(95% confidence intervals) at baseline and 12 weeks post randomisation, and changes in health and social care costs by trial arm**

| Measure | Time point | SIPC | | Standard care | |
|---|---|---|---|---|---|
| | | N | $\bar{x}$ (95%CI) | N | $\bar{x}$ (95%CI) |
| Inpatient care | Baseline | 176 | 1,667(1,111 to 2,223) | 174 | 1,738(1,090 to 2,387) |
| | 12 weeks | 158 | 899(445 to 1,353) | 161 | 1,169(677 to 1,662) |
| Outpatient care | Baseline | 176 | 109(88 to 131) | 174 | 101(82 to 120) |
| | 12 weeks | 158 | 95(67 to 123) | 161 | 90(60 to 119) |
| Day or Community care | Baseline | 176 | 119(57 to 182) | 174 | 95(38 to 153) |
| | 12 weeks | 158 | 124(53 to 194) | 161 | 132(57 to 207) |
| Home care | Baseline | 176 | 80(46 to 114) | 174 | 67(48 to 86) |
| | 12 weeks | 158 | 72(39 to 106) | 161 | 68(44 to 92) |
| Palliative care | Baseline | 176 | 1(0 to 2) | 174 | 14(-5 to 32) |
| | 12 weeks | 158 | 68(33 to 103) | 161 | 25(0 to 50) |
| Rehabilitation | Baseline | 176 | 384(47 to 721) | 174 | 100(-14 to 214) |
| | 12 weeks | 158 | 71(25 to 117) | 161 | 300(-60 to 661) |
| Primary care | Baseline | 176 | 51(40 to 62) | 174 | 49(37 to 61) |
| | 12 weeks | 158 | 43(32 to 54) | 161 | 41(31 to 51) |
| Social care | Baseline | 176 | 293(185 to 401) | 174 | 453(200 to 706) |
| | 12 weeks | 158 | 294(200 to 388) | 161 | 313(210 to 416) |
| Test & diagnostic | Baseline | 176 | 54(34 to 73) | 174 | 62(29 to 95) |
| | 12 weeks | 158 | 22(10 to 33) | 161 | 39(23 to 55) |
| Health & Social care | Baseline | 176 | 2,759(1,991 to 3,527) | 174 | 2,680(1,942 to 3,417) |
| | 12 weeks | 158 | 1,687(1,198 to 2,176) | 161 | 2,177(1,456 to 2,899) |
| Informal care | Baseline | 176 | 730(562 to 898) | 174 | 970(782 to 1,158) |
| | 12 weeks | 158 | 564(421 to 708) | 161 | 761(601 to 921) |
| Change in health & social care | Imputed | 176 | -1,076(-1,929 to -222) | 174 | -514(-1,448 to 419) |

[*] P value=0.12, it is for two group comparisons using generalised linear mixed model, adjusting for baseline values with centre modelled as a random effect.

© 2020 Gao W et al. *JAMA Network Open.*

## Results of qualitative data analysis – eTable 11-13

### eTable 11. Patient demographics for qualitative interview participants

| Variable | Value | Interviewed Patients N=26 |
|---|---|---|
| **Age,** mean (SD) | | 63.5 (13.5) |
| **Gender,** N (%) | Male | 14 (53.8) |
| | Female | 12 (46.2) |
| **Diagnosis,** N (%) | Multiple Sclerosis | 18 (69.2) |
| | Idiopathic Parkinson's Disease | 6 (23.1) |
| | Progressive Supranuclear Palsy | 2 (7.8) |
| **Years since diagnosis,** mean (SD)<br>Range | | 13.7 (10.5)<br>0-38 |
| **Comorbidities,** N (%) | No | 5 (19.2) |
| | Yes | 21 (80.8) |
| **Patient capacity,** N (%) | Consent | 24 (92.3) |
| | Personal consultee assent | 2 (7.7) |
| **Baseline IPOS Neuro-S8,** mean (SD)<br>Range | | 8.2 (4.1)<br>2-17 |
| **Living status,** N (%) | Alone | 6 (23.1) |
| | With spouse/partner and/or children | 16 (61.5) |
| | With friend(s)/with others | 4 (15.4) |
| **Ethnicity,** N (%) | White | 23 (88.5) |
| | Other ethnic group | 3 (11.5) |
| **Employment,** N (%) | No | 25 (96.2) |
| | Yes | 1 (3.8) |

### eTable 12. Carer demographics for qualitative interview participants

| Variable | Value | Interviewed Caregivers N=16 |
|---|---|---|
| **Age, mean** (SD) | | 58.9 (14.7) |
| **Gender,** N (%) | Male | 6 (37.5) |
| | Female | 10 (62.5) |
| **Relationship to patient,** N (%) | Spouse/partner | 11 (68.8) |
| | Son/daughter | 3 (18.8) |
| | Other | 2 (12.5) |
| **Ethnicity,** N (%) | White | 15 (93.7) |
| | Other ethnic group | 1 (6.3) |
| **Employment,** N (%) | No | 10 (62.5) |
| | Yes | 6 (37.5) |
| **Illness,** N (%) | No | 7 (43.8) |
| | Yes | 9 (56.2) |
| **Baseline ZBI-12,** mean (SD)<br>Range | | 20.9 (9.5)<br>(0-41) |

© 2020 Gao W et al. *JAMA Network Open.*

**eTable 13. Analytic framework for the qualitative data on the value and impact of SIPC**

| Theme/*Definition* | Sub-categories | Definition | Illustrative quote |
|---|---|---|---|
| **Adapting to losses and building resilience**<br>Key strategies for adjusting to increasing disability, declining function and nearness to end of life. | Care beyond medicines | Psychosocial interventions of skilled support, valuing and appreciating what life is like, and practical support.<br>Psychosocial interventions were valued to support resilience and adaptation and counter feelings of loneliness.<br>But with increasing complexity of psychosocial needs, the short-term nature of the intervention limited opportunity to build sufficient trust and rapport to enable patients and caregivers to engage in difficult conversations. | *We* [the palliative care nurse and I] *did talk about it* [feeling lonely and down sometimes]. *MS can be very frightening and lonely, and you know I'm grieving for my body and the life I had. I know this is still my house, but it doesn't feel like my house anymore. I do get quite down sometimes and then I think who I can talk to but then I use meditation to help me with that and to stay positive. I did see a counsellor many years ago and that was helpful so maybe that is something I should have asked for* [from the palliative care nurse].<br>[Patient P01348-F] |
| | Asked about everything | Planning future care for end of life experiences, expectations and impact.<br>Engagement ranged from 'not all' to 'I've been writing stuff down for years'.<br>Engagement was marked by uncertainty in 'not knowing what's going to take hold' and fear of increasing disability and loss of capacity.<br>The SIPC intervention seemed to be the start of a conversation. | …*she* [the palliative care nurse] *did say "Have you thought about the future?" and what your plans are for the future and stuff like that. I mean, I know that a lot people do recommend that you make plans and you think about what's gonna happen when she becomes more dependent. Errm you know, how you're gonna cope as a family, what kind of errm, what you're gonna do really. I'm really of the thought that you can't really plan too much when it comes to something like MS, dementia because you just don't know when things are gonna take hold. I mean things have taken hold and we're still coping.*<br>[Caregiver C01280-F] |
| **Attend to function, deficits and maintaining stability**<br>Optimising function and independence, and managing physical deficits and concerns | *Little things that make a big difference*<br>[P0139-M] | Optimal management of unstable symptoms to reduce distress (e.g. breathlessness, pain).<br>Key components for impact:<br>• Understanding complexity of symptom distress, 'quick fixes' were rare.<br>• Symptoms complex or refractory or change in medication worsening other symptoms.<br>• Availability of skilled practitioners 'to know people are available and willing to try and improve symptom management'<br>• Working with the person and caregivers to support management approaches honed overtime and prevent duplicating previously unsuccessful interventions e.g. medication for depression.<br>• Integrated working with neurology services to manage optimally medication.<br>• Timely response to unstable symptoms and concerns to prevent 'domino effect of decline'.<br><br>Maintaining function and independence by supporting adaptation and problem solving (e.g. continence, mobility, falls prevention, eating and drinking).<br>Key components were: involvement of the MDT (OT to support function, | *Well it's* [emotional concerns] *sort of linked to my physical feelings really so I didn't feel like I was ever gonna improve but I have begun to improve. I'm feeling a bit ill and fluey and that sort of winter feeling at the moment, but the massage* [SIPC therapist] *was the start I think of me feeling better and getting more sleep and feeling more generally well in myself. So it's a subtle change in some ways but in some ways it's quite quite a large thing cos just going to bed was a nightmare, I just couldn't sleep at all and it was making me more tired and it seemed to make the symptoms worse in the morning and during the day and so I think it's a good thing that I can actually go to bed and just sleep again since being on this course* [of massage and change to medication for spasm].<br>[Patient P05182-M]<br><br>*Well when I discussed my incontinence* [with palliative care CNS], *which is difficult to discuss with a guy* [my husband], *it's nice to talk to a lady about that sort of thing, I find that very helpful. She gave me some nice pointers as to what to do and how to overcome certain things which was very good you know because in the end though he does a lot for me, I love him [husband] but you can't talk about* |

| | | | |
|---|---|---|---|
| | | physiotherapy and complimentary therapies to provide non-pharmacological interventions). Lack of attention to function an area of frustration for patients and carers. | *some certain things, it's too personal you know…*<br>[Patient P01007-F] |
| | Maintain stability | Although living with extensive losses and deficits, individuals considered themselves 'stable' and as working continuously to maintain stability.<br>SIPC about skilled support by increasing awareness and understanding of management of symptoms and concerns.<br>For some no impact from involvement of SIPC considered stable no difference from symptoms and concerns used to living with; and equipment in place to maintain independence. | *Errm wouldn't say make a difference but she* [palliative care nurse], *she you know explained errm certain things how things would be helped in certain ways and that. Errm…. well just really just sort of (PAUSE) like with his errm bowel movement and that to give you know, if it's really bad, giving him Imodium every now and again which could possible help.*<br>[Caregiver C05275-F] |
| **Enabling carers to care**<br>Empowering carers | Enabling carers to care | Recognising the role of caregivers; valuing and acknowledging their work.<br>Complexity of caring with a tendency to put the person before themselves.<br>Not asking for help, but also frustrated when their needs are not considered.<br>Supporting caregivers from simple intervention of acknowledging and valuing, through to complex ongoing process requiring continuity of care. | *Whilst I don't think errm, there's not really a lot that she can do for me errm, that I can think of because I tend to sort of manage, you know. I manage as best I know how, you know errm, but I think it's the fact that she's, her intervention, you know, irrespective of what she was or wasn't able to do for us, I think meant a lot.*<br>[Caregiver C01319-F] |

© 2020 Gao W et al. *JAMA Network Open.*

## Analysis of fidelity data – eTable 14

To understand the delivery of the intervention, all multi-professional palliative care teams completed standardised documentation for each patient recording the main activities and services provided. Each team was advised to use their own existing paper based or electronic clinical records in order not to duplicate work for busy clinical teams. However, they were asked to review their usual documentation to ensure that as a minimum they record and report:

- Mode of contact and duration for each contact;
- Clinical details and severity of main problems;
- Activities performed during contact, plan of care and referrals to other services;
- Phase of illness (stable, unstable, etc.);
- Performance status using the Australian Modified Karnofsky Scale;
- Level of compliance using the following classifications: Complier (received full intervention as planned); Partial complier/erratic user (received some but not all of the intervention, or recommendations not followed); Overuser (in frequent contact with the service); and Dropout.

Of the 176 patients allocated to receive the SIPC, 173 patients received the intervention. Three patients did not receive the intervention as 2 withdrew from the trial and 1 could not be contacted by the respective palliative care team following referral. Of the 173 patients who received the intervention, all had an initial face-to-face visit for a comprehensive palliative care assessment. Following this, 152 patients had a second key worker contact (100 face-to-face and 52 by telephone) and 153 patients had a third key worker contact (91 face-to-face, 37 by telephone and 48 type of contact was not recorded). The intervention manual described the core elements to be covered when assessing patients as part of the SIPC as well as the abovementioned minimum standards for capturing and reporting delivery of the SIPC intervention. The completion rates of these core intervention elements have been analysed and will be published separately.

© 2020 Gao W et al. *JAMA Network Open.*
