# Supplementary material for: Effect of Short-term Integrated Palliative Care on Patient-Reported Outcomes Among Patients Severely Affected With Long-term Neurological Conditions: A Randomized Clinical Trial
Source: JAMA Netw Open. 2020 Aug 28;3(8):e2015061. doi: 10.1001/jamanetworkopen.2020.15061 (PMC7455856; doi:10.1001/jamanetworkopen.2020.15061)
Supplement: Supplement 3. — Data Sharing Statement [file jamanetwopen-3-e2015061-s003.pdf]

Data Sharing Statement

Gao. Effect of Short-term Integrated Palliative Care on Patient-Reported Outcomes Among Patients Severely Affected With Long-term Neurological Conditions. *JAMA Netw Open*. Published August 28, 2020. 10.1001/jamanetworkopen.2020.15061

Data
Data available: Yes
Data types: Deidentified participant data, Data dictionary
How to access data: wei.gao@kcl.ac.uk
When available: beginning date: 01-01-2022

Supporting Documents
Document types: None

Additional Information
Who can access the data: Anyone whose proposed use of the data has been approved
Types of analyses: for any purpose or for a specified purpose
Mechanisms of data availability: with investigator support, after approval of a proposal and with a signed data access agreement
